# REM sleep favors the restructuring of problem-related semantic associations

**DOI:** 10.1038/s42003-026-10354-1

**Published:** 2026-06-03

**Authors:** Théophile Bieth, Nicolas Decat, Yoed N. Kenett, Marie Scuccimarra, Marcela Ovando-Tellez, Alizée Lopez-Persem, Célia Lacaux, Saoussen Ben Younes, Arthur Le Coz, Sarah Moreno-Rodriguez, Isabelle Arnulf, Emmanuelle Volle, Delphine Oudiette

**Affiliations:** 1 Sorbonne University, Institut du Cerveau—Paris Brain Institute -ICM-, Inserm, CNRS, AP-HP Hôpital de la Pitié-Salpêtrière, Paris, France; 2 Neurology department, Pitié-Salpêtrière Hospital, AP-HP, Paris, France; 3 AP-HP, Pitié-Salpêtrière Hospital, Service des Pathologies du Sommeil, National Reference Center for Narcolepsy, Paris, France; 4 Faculty of Data and Decision Sciences, Technion—Israel Institute of Technology, Haifa, Israel

**Keywords:** Problem solving, REM sleep

## Abstract

REM sleep is considered a ‘hyperassociative’ state enhancing the connections between weakly related concepts, thereby facilitating memory restructuring — a critical process proposed in creative problem-solving. We empirically explore this hypothesis by testing participants on a riddle before and after a 90-minute incubation period including rapid eye movement (REM) sleep, only non-REM sleep, or wakefulness. We quantified memory restructuring by computing differences in problem-related semantic memory network (SemNets) properties between pre- and post-incubation. For each sleep stage, we extracted fine-grained electroencephalogram (EEG) measures linked to cognitive richness. REM sleep, compared to wake, reshaped the dominant problem representation by combining semantically remote concepts in memory and reducing strong but solution-irrelevant associations of ideas. Such REM sleep-related restructuring was associated with EEG measures indexing a richer cognitive state. While REM drove memory restructuring, it alone did not boost problem-solving success, suggesting that more extensive restructuring or additional processes are necessary for creative breakthroughs.

## Abstract (web summary)

Network science methodology sheds light on the role of sleep in memory restructuring and creative problem-solving. It empirically confirms the hyperassociative properties of rapid eye movement sleep at both behavioral and neural levels.

## Introduction

How are great ideas born? Famous inventors, scientists, artists^1^, and regular folks alike^2^ have long reckoned that the genesis of creative sparks can be traced back to our sleeping selves. Surprisingly, few studies have attempted to empirically test this popular belief.

Most research efforts in understanding the impact of sleep on cognition have instead focused on sleep function in memory. Robust experimental and electrophysiological evidence has shown the crucial role of sleep in transforming labile memory traces into stable and enduring ones^3–9^. This memory consolidation process is thought to occur in non-rapid eye movement (NREM) sleep preferentially and relies on an intricate coupling of neural activity across the neocortex, thalamus, and hippocampus^3,6,10–13^. In addition to directly strengthening/stabilizing individual memories, several studies have hypothesized that sleep also actively transforms memories^7,14–16^. For example, the BiOtA model^15^ proposed that memory reactivation during NREM sleep favors the formation of schema, whereas overlapping reactivations during subsequent rapid eye movement (REM) sleep would recombine memories and promote novel associations (i.e., memory restructuring). Because forming new connections between concepts in memory is at the core of the creative^17–22^ and insight problem-solving processes^23–31^, such sleep-related restructuring mechanism could foster creative problem-solving. This oft-cited hypothesis is found with slight variations in several recent theoretical accounts^7,14–16,32–35^. However, only a handful of studies provided experimental evidence in favor of a link between sleep and memory restructuring in general and between sleep-related memory restructuring and creative problem-solving in particular.

While all sleep stages may contribute to memory restructuring, REM sleep is often viewed as the most favorable period for this process to occur. Due to its unique neurochemical properties, REM sleep is considered a “hyperassociative” state^36,37^. First, both the high level of acetylcholine^38,39^ and GABA^40–43^ during REM sleep supports neural plasticity and synapse reshaping. This neurochemical environment facilitates dynamic changes in synaptic connectivity, which may manifest as either synaptic strengthening or pruning depending on the brain regions and specific neural circuits involved^40,44,45^. Second, endogenous activations by the ponto-geniculo-occipital waves^46^ may trigger spreading activations from the occipital cortex to random cortical areas, in turn facilitating novel combinations of memory representations^15^. Third, REM sleep presents a broader pattern of inter-regional connectivity than NREM sleep and wakefulness^47^. Fourth, compared to other stages, REM sleep is associated with a shift toward excitation evidenced by an increase of spectral power in high frequency bands, greater EEG complexity and a flattened aperiodic component of the EEG signal^48^. Such EEG measures have been linked to enhanced cognitive processing, allowing to discriminate between different states of consciousness (e.g., wake vs. anesthesia)^49^, but also between varying levels of engagement in a cognitive task during wakefulness^50,51^ and responsiveness to auditory stimuli during sleep^52^. These observations suggest that the complexity and nature of a neurophysiological state constrain the complexity of the cognitive processes it can support and might thus be particularly relevant for memory restructuring—a “complex” process requiring the formation of novel associations in memory. Although satisfying, this hypothesis remains to be tested. Finally, REM sleep-related mental imagery (i.e., dreams) may reflect or influence memory reprocessing by combining remote and recent experiences into novel scenarios^36,53,54^. Indeed, REM sleep dreams are typically longer^55–57^, more vivid, hallucinatory^58,59^, and metaphorical^36^ than NREM sleep dreams (even if this is debated, see ref. ^60^). REM sleep dreams also often include bizarre incongruities and discontinuities^58,61^ that are not random but seem to follow associative rules^62^.

Two experimental studies have provided indirect evidence in favor of the hypothesis that REM sleep is a hyperassociative state. These studies used a semantic priming task after awakenings from NREM and REM sleep, with the idea that sleep stages’ peculiar cognition persists during a short period of carry-over after awakening^37,63^. After awakening from REM sleep, participants were faster at identifying words that were weakly associated with the prime word (e.g., thief-wrong) than those that were strongly associated with the prime (e.g., hot-cold). It suggests that REM sleep not only favors distant semantic associations but also inhibits obvious ones. This idea is congruent with a recent theory proposing that REM sleep normalizes memory strength, so that weak and strong memory representations align in recallability^35^.

The relationship between sleep or sleep-related memory restructuring and creative problem-solving was investigated in several experimental studies, leading to disparate findings. Some studies used creativity tasks that were supposed to necessitate the formation of novel/distant semantic associations or a change in the representation of the problem (i.e., restructuring)^24^. For example, a study found that REM sleep facilitates solving of anagram word puzzles compared to NREM sleep^64^ and a recent study showed that greater REM sleep proportion during an afternoon nap was linked to successful analogical transfer in creative problem-solving, suggesting that REM sleep supports the formation of novel connections between source and target problems^65^. Other studies used the classical Remote Associates Task (or a variation of this task) in which three unrelated words must be combined to find a fourth one^21^. Performance was improved in participants who reached REM sleep^66^ or showed higher autonomic activity during REM sleep^67^, but only if solutions were first primed before sleeping. Other authors found a global positive impact of sleep on the same task performance, but either did not show a specific involvement of REM sleep in this effect^68^ or did not investigate it^69–71^. Finally, another study did not find a benefit of sleep on previously unsolved items when the task was repeated^72^. In parallel, other authors investigated the link between sleep and creative problem-solving using fundamentally different tasks, such as the number reduction task (i.e., involving extracting hidden regularities), a video-game, puzzles, or magic tricks. Some found a positive effect of sleep, either mediated by a full night of sleep^73^ or NREM sleep^74–77^, while others did not find any statistically significant impact of sleep on problem-solving^78–80^.

Hence, the literature linking sleep, memory restructuring, and creative problem-solving is inconsistent. A major methodological limitation of these studies is that successful problem-solving is often assumed to reflect the specific process under investigation (i.e., insight, cognitive schemata, memory restructuring)^81^, but this process itself is not empirically measured or operationalized. In the current study, we tackled this limitation by isolating and quantifying memory restructuring independently of solving success. We thus addressed the challenge of disentangling solving per se from one of the cognitive mechanisms that presumably lead to it. While capturing the memory restructuring process remains difficult^31^, we propose that network science methods, which provide quantitative tools to represent complex systems such as semantic memory^82,83^, constitute a powerful approach to overcome this challenge.

Network science applied to memory research allows building semantic memory networks (SemNet) in which nodes represent concepts in memory and are more or less connected depending on their semantic similarity^84–91^. Extensive past research has shown how SemNet properties relate to variation in creativity across groups and individuals^85,87,90,92^. This research has demonstrated how higher creative individuals exhibit a richer, more connected SemNet structure, that facilitates memory exploration and combination of remote ideas in creative ways^17,19,89,93,94^. We recently adapted this approach to objectively quantify memory restructuring in creative problem-solving^23,28^. We built problem-related SemNets aimed at approximating the mental representation of a specific problem. By computing network-based metrics that reflect the connective properties of a network^95^, we estimated the organization of the problem-related SemNet and quantified its reorganization after solving the problem at the individual level. We demonstrated that local changes in parts of the SemNets were instrumental in actively solving the problem^23^. Specifically, we described a remoteness-based restructuring corresponding to SemNet changes that made semantically remote concepts more related in solvers compared to non-solvers.

In the present study, we leveraged this approach to directly investigate the links between sleep (specifically REM sleep), memory restructuring, and creative problem-solving. We aimed to test whether REM sleep: (i) is a hyperassociative state, and (ii) favors creative problem-solving. Participants attempted to solve a riddle before and after an incubation period during which they were either encouraged to stay awake or to sleep. To quantify memory restructuring, we built problem-related SemNets before and after the incubation period and measured changes in these networks using several metrics that reflect: the strength of associations between concepts (*weight*), the interconnectedness of a node’s neighbors (*clustering coefficient*), the number of steps needed to connect concepts (*efficiency*), and the centrality/influence of nodes within the network (*eigenvector centrality*). Each of these metrics captures slightly different aspects of the SemNet connectivity, that is, how easily and efficiently information flows between concepts. In line with the hyperassociative hypothesis, we expected REM sleep to make SemNets more connected compared to wakefulness. We anticipated an increase in some or all SemNet metricsparticularly between semantically remote concepts, following REM sleep compared to before. We hypothesized that such REM sleep-related restructuring would facilitate problem-solving after sleep. Finally, in an exploratory analysis, we examined whether REM sleep-related memory restructuring and/or creative benefit would be reflected by specific EEG markers-such as faster cortical activity, higher complexity, or flatter spectral slope, all thought to reflect a neurophysiological mode that is rich, with a wide repertoire of possible neural configurations, and allowing for complex cognitive processes to take place. We hypothesized that the cognitive complexity of memory restructuring should likewise be reflected in a richer cortical state.

## Results

One hundred and eleven participants (mean age = 21.6 years, standard error of the mean, SEM = 2.22 years, 77 women and 34 men) were administered the same riddle as in our previous study^23^. The riddle states: “Zoe throws a stone that lands in the sky. How is it possible and in which context?”. To solve this puzzle problem, the initial dominant representation of the problem (based on the usual representation of the sky) needs to be restructured to produce a solution-relevant representation (e.g., the sky is a drawing on the floor). The solution is that Zoe plays hopscotch, a classical backyard children game in which players must throw a stone into parts of a diagram drawn on the ground and then hop on them. In France, the last part is traditionally represented by a half-circle named “sky”.

**Fig. 1:**
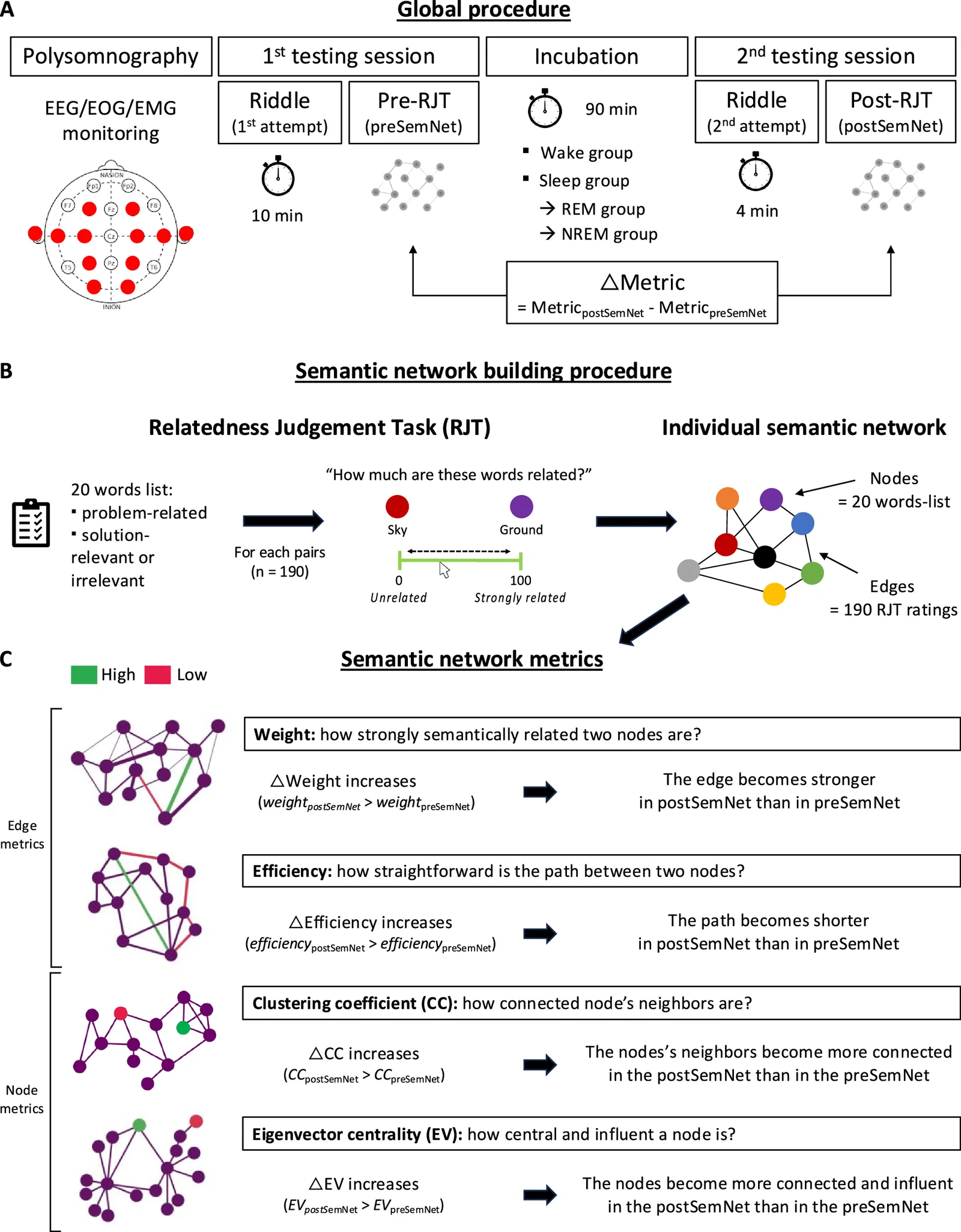
Experimental procedure. **A** Global design. Participants were first equipped with EEG, EMG, and EOG sensors to monitor sleep/wake states during the whole experiment (polysomnography). EEG layout in the 10–20 system is provided, with red circles indicating the position of electrodes on the scalp. Then, participants had a first testing session, during which they attempted to solve the riddle and completed the first RJT (preRJT). The incubation period corresponded to a 90-min break, in which participants were divided into two groups: a group that stayed awake (Wake group) and a group that took a nap (Sleep group, further divided into NREM and REM groups, depending on whether participants reached REM sleep during the nap based on the polysomnography recording). After the incubation period, participants underwent a second testing session, which included a new attempt to solve the riddle and the second RJT (postRJT). Changes in semantic network (SemNet) organization were assessed by computing the difference of network-based metrics between the postSemNet and the preSemNet (ΔMetric). **B** Semantic network building procedure. During the relatedness judgment task (RJT), participants were asked to rate the strength of semantic relatedness between two words on a visual scale from 0 (“unrelated”) to 100 (“strongly related”). Word pairs composing RJT trials were all possible combinations (*n* = 190) of a pre-established word list consisting of 20 words that were related to the problem and could be solution-relevant or irrelevant (see Supplementary Table 1 for the list of words). Then, individual SemNets were built, where nodes were the 20 words of the word list, and edges were the individual RJT ratings. Finally, four mathematical metrics were computed, representing different connectivity properties of the SemNet. **C** Semantic network metrics. We illustrated on artificial SemNets the four metrics we used (*weight*, *efficiency*, *eigenvector centrality*, and *clustering coefficient*) to characterize the SemNet connectivity properties, indicating their meaning at the network level. Explicit examples were shown in the SemNets highlighting edge or node with high (in green) or low (in red) metric values. Finally, we clarified for each metric what the ΔMetric means in terms of SemNet changes (preSemNet and postSemNet represent SemNet before and after the incubation period, respectively).

To directly test the impact of sleep on solving success, participants were asked to solve the riddle in a limited time before and after a 90-min incubation period, during which they were instructed to either nap (Sleep group, *n* = 83) or stay awake (Wake group, *n* = 28; mean age = 22.9 years, SEM = 0.59 years, 20 women and 8 men). Participants’ sleep/wake states were monitored through polysomnography (Fig. 1A). During the resting period, the Wake group worked on a 1000-pieces jigsaw puzzle, a task designed to be cognitively engaging and typically requiring more than 6 h to complete. Participants in the Wake group were explicitly instructed not to rest. The Sleep group was further divided into two sub-groups, depending on whether participants reached REM sleep (REM group, *n* = 52; mean age = 21.1 years, SEM = 0.23 years, 33 women and 19 men) or not (NREM group, *n* = 31; mean age = 21.3 years, SEM = 0.23 years, 24 women and 7 men) during the nap based on the international sleep scoring criteria^96^.

To quantify memory restructuring, we used the network science methodology that we developed in our previous study^23^. We built individual-based SemNets before and after the incubation period based on each participant’s relatedness judgments of riddle-specific word pairs (Relatedness Judgment Task, RJT; Fig. 1B)^85,86,89,90^. These SemNets represent elements of problem-related concepts as nodes, with edges reflecting the relationship between these concepts in each participant’s memory, according to their individual RJT ratings (Fig. 1B). Then, we calculated established network-based SemNet metrics before and after the incubation period. These metrics quantified how much edges were strong (*weight*) or efficient (*efficiency*), but also how interconnected the nodes were (*clustering coefficient*), and how central nodes were within the network (*eigenvector centrality*) (Fig. 1C). We selected these metrics because they quantify different facets of SemNets connectivity properties and have been associated with creative problem-solving success^23^. We measured incubation-related changes in SemNets organization by calculating the difference in these metrics between the pre- and post-incubation SemNets (Fig. 1A)^23^. We explored global as well as local SemNet changes targeting the most semantically distant edges and nodes as determined by a normative SemNet composed of the same nodes (see “Methods”).

Several participants (*n* = 46) were excluded, 25 because they solved the riddle in the first solving phase, two because they fall asleep during the resting period while in the wake group, and 19 for various task-specific reasons (see “Methods”). The final sample included 84 participants in the behavioral analyses, and 65 participants in the SemNet analyses (Supplementary Fig. 1). For EEG analyses, we included only participants in the REM and NREM groups (*n* = 48, see “Methods”). Table 1 summarizes the amount of sleep (in total and by sleep stages) achieved by these participants.

**Table 1:** Summary of sleep measures in the NREM and REM groups

		NREM group		REM group	
NREM	N1	5.62 (1.22)	5.08 (0.68)		W(46) = 265.9, *p* = 0.91
	N2	30.79 (4.15)	44.48 (2.12)		W(46) = 126.5, *p* = 0.003
	N3	3.97 (2.38)	10.29 (2.16)		W(46) = 126, *p* = 0.002
REM		0 (0)	15.58 (1.83)		W(46) = 0, *p* = 5.56 × 10^−9^
WASO		26.65 (4.44)	7.10 (1.17)		W(46) = 451, *p* = 5.40 × 10^−5^
TST		40.38 (5.55)	75.44 (2.22)		W(46) = 45.4, *p* = 2.74 × 10^−6^

The amount of sleep (in min) of participants in REM and NREM groups is detailed for each sleep stage (mean and standard error of the mean—SEM). We specified the total sleep time (TST), and the amount of wake after sleep onset (WASO). We indicated statistical differences between REM and NREM groups according to a Wilcoxon test.

### Sleep incubation did not enhance problem-solving abilities

During the first solving phase (i.e., before the incubation period), the overall solving rate of the riddle was 22.5% (*n* = 25/111 solvers, 13 with insight report). After the incubation period, ten additional participants found the solution (11.9% of the 84 initial non-solvers). Correct solutions to the riddle were associated with a higher incidence of insight and greater confidence than incorrect responses, in both the first and second solving phases (see Supplementary Note 1, Supplementary Fig. 2).

We found no significant Group effect on the percentage of participants who solved the riddle after the incubation period (Wake: 20%, *n* = 4 solvers/20, 2 with insight report; NREM: 5%, *n* = 1 solver/20 with insight report; REM: 11.4%, *n* = 5 solvers/44, 4 with insight report; Fisher test, *p* = 0.36). Of note, we also tested whether the type of incubation period (Wake, NREM, REM) impacted the proportion of participants who got closer to the solution when they did not find it. While there was a trend for the REM group to get closer to the solution, this result did not reach significance (see Supplementary Note 2). The low solving rate did not allow us to explore whether REM sleep-related restructuring can be associated with successful problem-solving.

### REM sleep promotes restructuring of problem-related associations

Using mixed-effects analyses of variances (ANOVA), we investigated whether SemNet changes (ΔMetric) differed between groups (Group: Wake [*n* = 17], NREM [*n* = 17], and REM [*n* = 31]) and whether these changes targeted semantically remote edges or nodes. Semantic remoteness of problem-related word pairs was measured in a normative SemNet built independently of the context of the problem^23^ to obtain a *semantic distance* variable for each pair of nodes (see “Methods”). Based on hyperassociativity theories of REM sleep, we expected an overall increase of SemNet changes (i.e., higher *weight*—more semantically linked nodes, higher *efficiency*—closer nodes in the SemNet, higher *clustering coefficient*—nodes with more interconnected neighbors, and higher *eigenvector centrality*—more central nodes) in the REM group compared to Wake and NREM groups (i.e., a main effect of Group). In addition, we expected to specifically observe these SemNet changes in semantically remote word pairs (Group × semantic distance interaction effects).

**Fig. 2:**
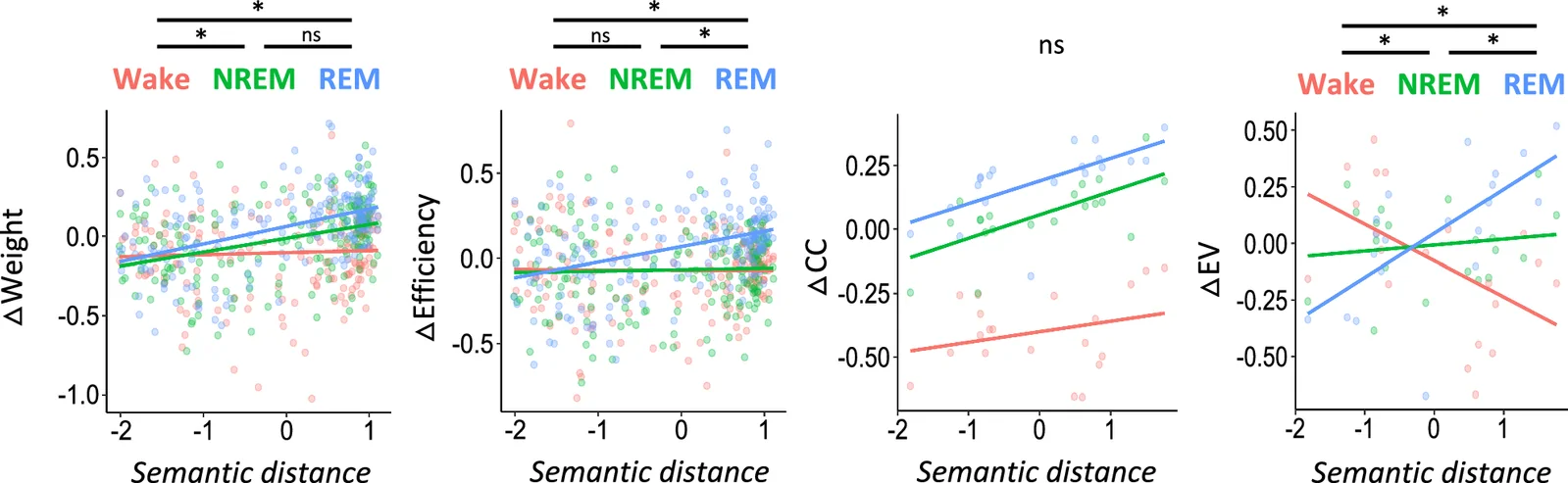
REM sleep was associated with remoteness-based SemNet changes. △Metric as a function of *semantic distance*. Each dot represents an edge or a node with its corresponding *semantic distance* value and averaged △Metric across participants. The lines depict the regression line between the two variables for each group (Wake, *n* = 17; NREM, *n* = 17; and REM, *n* = 31). For all figures, the three groups (Wake in red, NREM in green, and REM in blue) and the four metrics (*weight*, *efficiency*, *clustering coefficient*—CC, and *eigenvector centrality*—EV) are displayed. *: *p* < 0.05 (after correction for multiple comparisons), ns: non-statistically significant differences.

We found a statistically significant interaction effect between *semantic distance* and Group on ΔMetric for *weight* (F(2,11576) = 8.92, *p* = 1.35 × 10^−4^, *η*^2^ = 0.001), *efficiency* (*F*(2,11355) = 11.89, *p* = 6.93 × 10^−6^, *η*^2^ = 0.002), and *eigenvector centrality* (*F*(2,1232) = 14, *p* = 9.74 × 10^−7^, *η*^2^ = 0.02) (Fig. 2). Post-hoc analyses revealed that the slope (representing the relationship between semantic distance and ΔMetric) was significantly steeper in the REM group compared to the Wake group (*weight*: F(1,8159) = 17.17, *p* = 3.45 × 10^−5^, *η*^2^ = 0.002; *efficiency*: *F*(1,9070) = 17.59, *p* = 2.77 × 10^−5^, *η*^2^ = .002; *eigenvector centrality*: *F*(1,910) = 26.71, *p* = 2.91 × 10^−7^, *η*^2^ = 0.03). In addition, post hoc tests showed significant differences between the REM and NREM groups for *efficiency* (*F*(1,9070) = 13.68, *p* = 2.18 × 10^−4^, *η*^2^ = 0.001) and *eigenvector centrality* (*F*(1,910) = 5.82, *p* = 0.016, *η*^2^ = 0.006), but not for *weight* (*F*(1,8521) = 0.68, *p* = 0.41). Finally, the NREM group had a steeper slope compared to the Wake group for *eigenvector centrality* (*F*(1,644) = 6.79, *p* = 9.38 × 10^−3^, *η*^2^ = 0.01) and *weight* (*F*(1,6160) = 9.40, *p* = 2.18 × 10^−3^, *η*^2^ = 0.001), but not for *efficiency* (*F*(1,6424) = 0.18, *p* = 0.68). These results indicate that REM sleep enhanced SemNet changes by increasing the centrality (*eigenvector centrality* increased) and the connection (*weight* and *efficiency* increased) of nodes and edges that were semantically remote. The semantic distance × Group interaction effect was not statistically significant for *clustering coefficient* (*F*(2,1232) = 0.77, *p* = 0.47).

In addition, the main effects of Group on ΔMetric did not survive the correction for multiple comparisons (see Supplementary Note 3, Supplementary Fig. 3), suggesting that the impact of sleep on SemNet restructuring is mainly local rather than global.

Since participants in the REM group slept significantly longer than participants in the NREM group (Table 1), we conducted additional analyses to test whether the SemNet changes (ΔMetric) differed as a function of total sleep duration and *semantic distance*. No significant main or interaction effects on ΔMetric were found across the four metrics (see Supplementary Note 4), suggesting that the REM sleep-related SemNet changes were not driven by differences in the total sleep duration between the REM and NREM groups.

Finally, because our sample size was not equivalent due to methodological constrains (see “Method”), we provided control analyses by randomly and iteratively subsampling the REM group to make the three groups equivalent in terms of sample size (see Supplementary Note 5) and run the same behavioral analyses. We confirmed that our main results (significant interaction effect for *weight*, *efficiency* and *eigenvector centrality*) remained significant for more than 95% of the iterations (Supplementary Fig. 4).

To further interpret these results, we ran additional exploratory analyses detailed in Supplementary Note 2. Briefly, we assessed whether REM sleep would reduce the strength of the semantic associations related to the dominant, but solution-irrelevant, problem representation. This dominant representation was determined based on the most common but irrelevant responses to the riddle in an independent dataset of 160 participants. These results suggest that REM sleep decreases the centrality of concepts related to the dominant representation of the problem or increases the centrality of the non-dominant one (Supplementary Fig. 5).

Overall, our results support our hypothesis regarding the role of REM sleep on SemNet changes. REM sleep was mainly associated with local remoteness-based SemNet changes. The findings are consistent with the hyperassociative hypothesis of REM sleep and with the role of this stage in a mental restructuring of the problem, away from the dominant representation.

### SemNet restructuring is associated with EEG markers indexing a richer cognitive state during REM sleep

We investigated whether specific EEG features computed during the nap could be related to SemNet changes observed at the behavioral level. We excluded participants from the Wake group because they did a jigsaw puzzle during EEG recordings, leading to substantial noise in the EEG signal and low interpretability. In the Sleep group, using mixed-effects ANOVA, we investigated whether SemNet changes (ΔMetric) differed as a function of *semantic distance*, and EEG measures. We extracted several EEG measures that have been shown to track cognitive and consciousness state modifications in previous studies, revealing the level of complexity and richness of these states^48–50,52,97,98^. These EEG markers, computed independently for each sleep stage (N1, N2, N3, REM sleep), included a measure of the signal complexity (permutation entropy in theta frequency band—PE_θ_), the oscillatory component of the spectral power in the classical frequency bands (delta, theta, alpha, and beta power), as well as an aperiodic component (1/f spectral slope—Slope_1/f_). We ran a statistical model for each EEG measure (*n* = 6) computed during REM and NREM (that combined N1, N2, and N3 sleep stages) using the four SemNet metrics (*weight*, *efficiency*, *eigenvector centrality*, and *clustering coefficient*). Results were corrected for multiple comparisons (FDR method). Based on our behavioral results associating restructuring processes with REM sleep, we speculated that remoteness-based restructuring would occur in a richer cognitive state characterized by a higher PE_θ_ and EEG power in high-frequency bands (theta, alpha, beta), a flattening of the Slope_1/f_, and a lower power in low-frequency bands (delta) during REM.

**Fig. 3:**
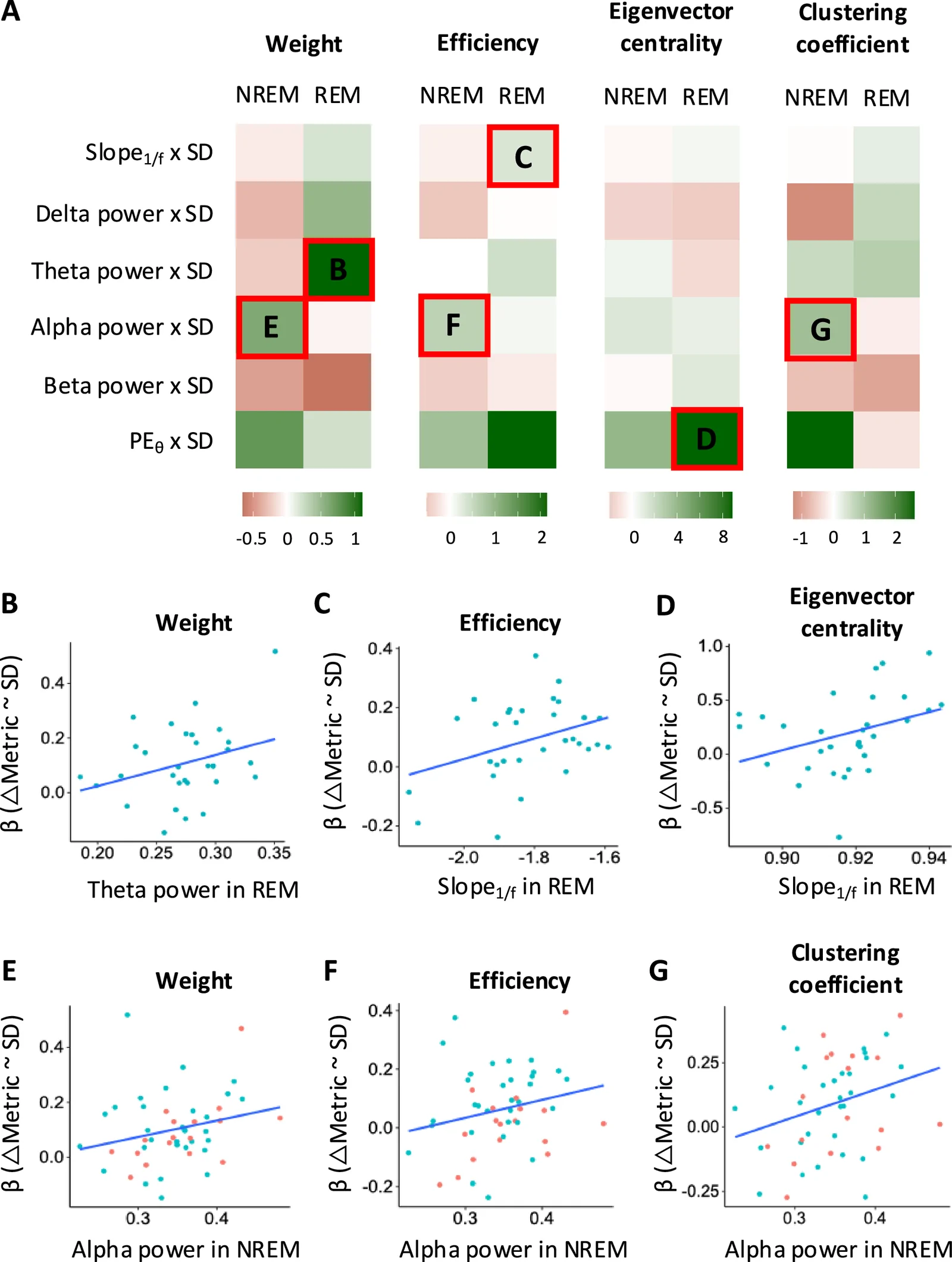
Semantic-based SemNet changes are associated with EEG markers indexing a richer cognitive state during REM and NREM sleep. Heatmaps (**A**) represent the statistical results for each EEG markers (rows) computed during REM (*n* = 31) and NREM (*n* = 48) sleep (columns) for the four metrics. The color code indicates the regression coefficients of the EEG × *semantic distance* (SD) interaction factor, ranging from red (negative values) to green (positive values). Squares framed in red indicate a significant *p* value (i.e., <0.05 after FDR correction). Letters inside the red-framed squares correspond to the figure panels that provide additional graphical representations of these results (from **B** to **G**), showing the regression coefficient between ΔMetric and SD as a function of an EEG marker with each dot representing an individual participant using a color code indicating their group (REM group in blue and NREM group in red).

Remoteness-based SemNet changes were associated with several EEG markers during REM and NREM sleep stages indicating different levels of cognitive richness (Fig. 3A). In line with our hypothesis, we found that larger remoteness-based SemNet changes during REM sleep were associated with a higher power in theta frequency band (*weight*: *F*(1,5415) = 9.62, *p* = 1.93 × 10^−3^, *η*^2^ = 0.002, Fig. 3B), a flattening of the Slope_1/f_ (*efficiency*: *F*(1,5857) = 14.13, *p* = 1.73 × 10^−4^, *η*^2^ = 0.002, Fig. 3C), and a higher PE_θ_ (*eigenvector centrality*: *F*(1,587) = 8.78, *p* = 3.17 × 10^−3^, *η*^2^ = 0.01, Fig. 3D). In addition, we found a higher remoteness-based SemNet changes associated with a higher power in alpha frequency band (*weight*: *F*(1,8521) = 9.06, *p* = 2.62 × 10^−3^, *η*^2^ = 0.001, Fig. 3E; *efficiency*: *F*(1,9070) = 9.90, *p* = 1.66 × 10^−3^, *η*^2^ = 0.001, Fig. 3F; *clustering coefficient*: *F*(1,910) = 7.61, *p* = 5.93 × 10^−3^, *η*^2^ = 0.008, Fig. 3G) during NREM sleep.

Overall, these results indicated that remoteness-based SemNet changes were associated with a more activated brain state during REM sleep, extending our behavioral results and supporting our hypothesis. We also found an association between alpha power during NREM sleep and SemNet changes, suggesting that semantic restructuring processes may be first initiated in NREM sleep. However, this result is tricky to interpret as we combined several sleep stages (N1, N2, N3), each characterized by distinct levels of consciousness. To provide a broader vision of EEG activity during NREM sleep, we ran an exploratory additional analysis separating N1, N2, and N3. Several of our EEG measures were associated with remoteness-based SemNet changes during N1, N2, and N3 sleep stages (Supplementary Fig. 6, see Supplementary Note 6). As these EEG changes do not all go in the same direction, they should be considered with caution.

## Discussion

Using network science tools assessing changes in individual SemNets organization, we explored whether and how sleep contributes to reorganizing individuals’ semantic associations related to a problem (i.e., restructuring) and how this process is related to solving success. Confirming our hypotheses, we found that REM sleep yields a restructuring of semantic associations mainly targeting the semantically remote associations of ideas in problem-related concepts. Specifically, we showed that REM sleep weakened the importance of problem-related concepts associated with the dominant problem representation and reinforced those associated with the alternative, non-dominant ones. Exploratory EEG analyses showed that higher remoteness-based SemNet changes were associated with a richer cognitive state during REM sleep. Hence, our study provided empirical evidence that REM sleep qualitatively transforms one’s memories by restructuring semantic associations related to a problem and that this relates to measurable EEG patterns. Of note, the absence of difference between the NREM and REM groups in some of our behavioral analyses and the NREM-related EEG results also suggest that the contribution of sleep in restructuring processes may be more complex, possibly involving the different NREM sleep stages that precede REM sleep. Finally, this sleep-related restructuring of semantic memories did not seem sufficient to promote successful solving, as participants who slept (both in the REM and NREM groups) did not show a higher solving rate than those who did not.

Our main behavioral findings, showing that REM sleep promotes memory restructuring, are in line with several theoretical accounts^7,15,16,32,72^ and empirical studies suggesting that REM sleep facilitates the combination of remote associations^66,67,99^ or strengthens the link between weakly associated memories^37,63^. In those studies, REM sleep-related restructuring was suggested as a potential mechanism explaining performance in cognitive tasks, but was not operationalized as a measurable variable. Our study goes a step further with two main additional findings. First, by quantifying restructuring using science network tools, we described how problem-related associations were restructured after sleep, and identified which parts of the SemNets specifically changed (i.e., semantically remote nodes). Second, we found that REM sleep-related changes in SemNets were smaller for associations that were dominant in the initial representation of the problem than for non-dominant ones. This result is consistent with previous studies suggesting that the inhibition of obvious associations is a key process for creative problem-solving^20,100^. Our findings also are in line with the forgetting fixation hypothesis^101^, stating that incubation may be helpful only when fixations were present beforehand, as it helps individuals overcome these fixations. In line with this view, a recent study^102^ showed that targeted reactivation of previously unsolved problems during NREM sleep preferentially facilitated solving of fixating puzzles over less-fixating ones, but the specific role of REM sleep was not examined. Our results suggest that REM sleep may play a key role in overcoming mental impasses triggered by initial, obvious interpretations of the problem, facilitating the exploration of alternative problem representations through restructuring processes. Of note, the effect of REM sleep on memory restructuring cannot be attributed to the second solving attempt after the incubation period, as this opportunity was provided to all groups equally.

Substantiating our behavioral results, we found that remoteness-based restructuring during REM sleep was associated with a higher level of brain activation as indexed by a higher EEG complexity, faster EEG activity, and a flatter spectral slope. This result suggests that such EEG measures can not only capture differences between various states of consciousness^49,97^, but may also reflect fine-grained differences in the level of cognitive processing happening within a same vigilance state. This is in line with a few recent studies which showed variations in the values of these EEG markers during simple cognitive tasks performed during wakefulness^50,51^, the sleep onset period^103,104^ and bona-fide sleep^52^. In addition, to demonstrate that restructuring occurs during sleep, we extend these previous accounts to a more complex cognitive process, showing that the quality of brain activity during REM sleep (measured by EEG markers) is a key determinant of the extent of restructuring. This provides an interesting mechanistic link between the neurophysiological properties of the sleeping brain and the dynamic of offline semantic memory organization. Overall, our study provided empirical evidence supporting the oft-cited theory that REM sleep fosters associative changes in individuals’ mental representations.

In contrast with our initial hypothesis, REM sleep did not promote solving success in our study. This negative result is at odds with previous studies showing that this sleep stage increases creative performance^64,66,67^. However, such a REM sleep-related boost in creativity has not been consistently replicated^68,72^. In all those studies, whether they reported positive or negative results, memory restructuring was solely inferred based on the measure of creative performance. By disentangling restructuring from the creative performance itself, our approach might be useful in reconciling discrepancies in the literature. As discussed before, REM sleep-related restructuring may help overcome a potential mental impasse by generating alternative representations. However, overcoming an impasse may not be enough for successful problem-solving, and additional mechanisms during subsequent wakefulness may be required, such as efficient navigation within the newly restructured SemNet. One potential interpretation is that restructuring (or associative) processes might be necessary but not sufficient for solving the problem^22,23,105^. Overall, by measuring what is restructured after sleep, our approach allows to get closer to the cognitive mechanisms related to the way individuals tackle a creative problem.

Other, non-mutually exclusive hypotheses could explain why REM sleep did not enhance solving success in our study. First, the riddle might have been too difficult (22.5% and 11.9% of solvers in the first and second solving phases, respectively). This idea is consistent with previous studies showing no benefit of sleep on difficult problems including magic tricks^80^ or an ambiguous crime riddle^79^.

Second, studies showing a beneficial impact of sleep on creative problem-solving often had in common that the solution was embedded in the material presented before sleep, either as a hidden regularity^64,73,103^ or as a response in a seemingly irrelevant task^66,67^. In our case, the RJT implicitly provided participants with words related to the solution before sleep, but not the solution itself. Furthermore, these solution-relevant associations may not have been salient enough because of their low proportion compared to the total number of word pairs rated during the RJT, which included solution-irrelevant or even misleading ones. In line with this idea, participants almost never found the solution during or immediately after the RJT (less than 0.5% of participants in our experience with this material^23^).

Third, participants were not informed that they would be tested again on the riddle after the incubation period. As a result, it is possible that other, more personally relevant material was preferentially processed during REM sleep instead of our riddle. This interpretation aligns with previous research demonstrating that sleep tends to prioritize the consolidation of memories deemed important for future use^106–108^.

Fourth, the REM sleep-related restructuring effect may not be sufficient to induce successful problem-solving on its own. For instance, additional restructuring, potentially during longer REM sleep periods, might be necessary to reach the optimal problem representation required for solving success. This interpretation is consistent with insight theories^24,109^, proposing that restructuring needs to exceed a threshold in order to be consciously integrated and lead to an insight solving.

Finally, a more integrative hypothesis, supported by theoretical models^14,15,32,35,110^, is that NREM and REM stages work in tandem across the sleep cycle: NREM supports the initial stabilization and offline consolidation of memory traces, which then serve as the substrate for REM-dependent associative restructuring. In our study, participants had a 90-min nap, a duration that might have restricted both the depth of NREM-mediated consolidation and the subsequent opportunity for REM sleep to act upon a sufficiently stabilized memory trace. Future studies using longer sleep periods or multiple sleep cycles could help disentangle the distinct and interactive contributions of NREM and REM stages to creative problem-solving.

We acknowledge that our study has some limitations. First, the low solving rate of the riddle prevented us from testing all announced hypotheses. However, using an easier riddle might not be ideal either as it would increase the risk of too many solvers before incubation, leaving little room to observe changes between pre- and post- incubation. We cannot exclude that the limited number of solvers may be partially attributable to participants’ unfamiliarity with the hopscotch game, as we did not control for this factor. Second, due to practical constraints associated with building problem-related SemNets (including a long-time commitment and the need for riddle-specific semantic material), we chose to use only one riddle in this study (although our method was validated using four different riddles^23^), with a limited number of words that may restrict the identification of less common associative pathways to the solution. In addition, according to Schilling’s theory^30^, insight leads to the creation of new edges or “shortcuts” in SemNets. Thus, while individuals vary in the associative paths they take when trying to solve the riddle, the end result should be similar in its effect on their SemNet, i.e., local memory restructuring as measured with our SemNet metrics. Our approach provides tools and methods to generalize the results using multiple riddles and larger SemNets across different sessions in future studies. Third, generalizability could also be improved by recruiting a larger sample with a balanced gender distribution and an equal proportion of participants in each group, as well as by better controlling for circadian and individual homeostatic factors that may influence sleep and cognition, such as menstrual cycle, lifestyle, and the amount of sleep the night before the experiment. Indeed, although our findings support a role of REM sleep in memory restructuring, it remains unclear what factors or individual differences may have influenced participants’ specific sleep patterns in the first place. More research is needed to identify the optimal conditions for enhancing REM-sleep related cognitive processes and to determine if these processes are influenced by individual sleep dynamics.

Another limitation is that we used different metrics to assess sleep-related restructuring effects, but not all of them were statistically significant. We used metrics commonly used in cognitive research that were previously associated with creativity traits and creative problem-solving in previous studies^23,83,86–88,90,94^. Interestingly, *weight*, *efficiency*, and *eigenvector centrality* were the metrics that captured statistically significant SemNet changes depending on the incubation period, as they did in our previous study^23^. However, direct replication of our previous findings is not possible here due to key differences in the experimental designs (Supplementary Fig. 7). Specifically, the current study lacks a pre-problem baseline assessment and includes an intervening incubation period with experimental manipulation between the two SemNet measurement time points, whereas our previous study computed networks before and after the solving phase without interruption. Despite these methodological differences that limit direct comparability, we nevertheless found several results that are consistent with our primary findings (see Supplementary Note 7).

Finally, because REM sleep occurs after other sleep stages, we were unable to isolate the effect of ‘pure’ REM sleep independently from NREM sleep. A few studies have highlighted NREM sleep as a state favorable to problem-solving^74–76^. Promising studies also put forward the potential role of the sleep onset stage (N1) for creative thinking and insight problem-solving^103,111^. In our study, we found a higher remoteness-based SemNet changes in the NREM group than in the Wake group for one of our local metrics (*eigenvector centrality*). The neural mechanisms underlying the restructuring of semantic associations and when exactly such a reorganization occurs during the nap remain unknown. In an exploratory analysis, we found several EEG features associated with restructuring processes that differed according to the sleep stage. Even though we applied a rigorous correction for multiple comparisons, the results must be interpreted with caution and need to be replicated in further studies. In particular, further researchs are needed to clarify the role of each NREM sleep stage and whether NREM sleep is necessary to initiate the restructuring processes that would unfold during subsequent REM sleep. Moreover, our groups also differed in sleep fragmentation and time spent in different NREM stages, which may represent additional factors that could contribute to semantic restructuring either independently or in interaction with REM sleep processes.

In sum, we provided evidence that REM sleep promotes reorganization of semantic memory, reflected in changes in problem-related SemNets properties (behavioral results) and that this restructuring is linked to richer brain activity patterns during REM sleep (EEG results). In particular, these SemNet changes bring remote associations closer together, potentially allowing to drift away from dominant, irrelevant ideas for solving the problem. Hence, our findings support previous theoretical models that highlighted the link between REM sleep and the recombination of semantic associations. However, REM sleep did not enhance the solving rate at the behavioral level, suggesting that additional processes are necessary for successful creative problem-solving.

Overall, our study confirmed that evaluating individual SemNets changes is a promising approach for exploring external factors that may influence restructuring processes and creativity. Even though restructuring did not convert into successful solving, our findings provide scientific support that ‘sleeping on a problem’ could help overcome an impasse. Our study also has broader implications. For instance, our SemNet approach could be applied to test the implication of memory restructuring (e.g., for learning) in early development, when REM sleep is particularly prominent, but also to explore how the restructuring of emotional memories is affected in neurological and psychiatric disorders where REM sleep disturbances are frequent (e.g., in Parkinson’s disease, depression, post-traumatic stress disorder).

## Methods

### Participants

For this study, we predefined a target sample size of 120 participants, aiming for approximately 30 participants per group. This calculation factored in potential no-shows, cancellations, and the possibility that some individuals in the sleep groups might not fall asleep. One hundred and eleven native French speakers aged between 18 and 34 years old (mean age = 21.6 years, SEM = 2.22 years, 77 women and 34 men) participated in this study. Participants were recruited through the Relais d’Information sur les Sciences de la Cognition (RISC), a platform dedicated to facilitating volunteer recruitment for cognitive experiments. Due to the platform’s predominantly female volunteer base, achieving a fully gender-balanced participant pool was not feasible. All participants were healthy adults with no history of neurological and/or psychiatric illness and no psychoactive substance abuse. In addition, they did not have any sleep disorder or environmental factors that could impact their sleep schedules (e.g., working at night). A local ethics committee approved the study (CPP Ile-de-France III, 2019-A00562-55). All participants gave their written informed consent and received 50€ as a financial compensation for their participation. All ethical regulations relevant to human research participants were followed.

Several participants were excluded for various task-specific reasons that are detailed in the dedicated methodological section. The final sample included 84 participants for the behavioral analyses and 65 participants for the SemNet analyses (Supplementary Fig. 1).

### Experimental procedure

Participants underwent a 4h-experiment during which they attempted to solve a riddle twice, separated by an incubation period of 90 min (Fig. 1A). The whole experiment took place in a dedicated room at the Sleep Department of the Pitié-Salpêtrière hospital. During the incubation period, participants were asked to either have a 90-min nap (Sleep group, *n* = 83, 75%; mean age = 21.2 years, SEM = 0.18 years, 57 women and 26 men) or to stay awake (Wake group, *n* = 28, 25%; mean age = 22.9 years, SEM = 0.59 years, 20 women and 8 men). In the Sleep group, participants were lying on a bed in a quiet room with the light off. They were instructed to relax and not resist sleep if it came. Conversely, in the Wake group, participants did not sleep at all and served as a control group. To keep them awake without actively thinking of the problem, they were instructed to complete a jigsaw puzzle (a game in which you need to combine pieces to obtain a picture). The jigsaw puzzle was composed of enough pieces (*n* = 1000) to keep participants engaged during the whole incubation period (no participants finished it at the end of the 90-min incubation period). There were two jigsaw puzzle version that had no link with the experiment content. All participants’ sleep/wake states were continuously monitored using standard polysomnography, which includes a monitoring of brain activity (EEG), chin muscle tone (EMG), and eye movements (EOG). Thus, we could confirm whether participants in the Wake group remained awake. Two participants were excluded because they fell asleep during the incubation period (Wake group: *n* = 26; Supplementary Fig. 1). In addition, based on their sleep stages during the nap, the Sleep group was naturally divided into two groups: the NREM group (*n* = 31; mean age = 21.3 years, SEM = 0.23 years, 24 women and 7 men) that included participants who only had NREM sleep during the nap, and the REM group (*n* = 52; mean age = 21.1 years, SEM = 0.23 years, 33 women and 19 men), which included those who also reached REM sleep. As our main objective concerns REM sleep, we increased the chance that participants will reach REM sleep during the 90-min nap, by asking all of them (even those in the Wake group) to reduce their usual sleeping time by 30% the night before the experiment (but no less than 5 h of sleep in total) and to come early in the morning (7:00 am) when REM sleep pressure is higher. Surprisingly, we observed a proportion of REM sleep in the Sleep group larger than expected (about 2 participants with REM sleep for 1 with NREM sleep only) leading to an unbalanced sample size across groups. However, increasing the sample size would likely not alter this distribution significantly, and recruiting participants during an afternoon nap (to maximize NREM sleep over REM sleep) would introduce potential circadian confounds. To account for this factor, which may influence our statistical analyses, we proposed additional control analyses, as detailed in Supplementary Note 5.

Before the incubation period, participants were presented with the riddle and attempted to solve it within 10 min. Then, participants performed a Relatedness Judgment Task (RJT) that allowed us to build their individual SemNets^85^. Participants were then informed of their assignment to either the Sleep or Wake group but were not informed that they would undergo another session of solving and the RJT after incubation. The same procedure (riddle followed by RJT) was applied after the incubation period, but with a shorter solving time.

All tasks were programmed using the Psychopy software^112^, running on a dedicated computer. Task-related instructions were initially explained and repeated before the beginning of each task. Before and after performing the tasks previously described, participants performed an additional, independent task that are out of the scope of this study and not detailed here. Specifically, this task involves non-semantic and figural material that does not interact with the current experiment and was performed by all participants regardless of their group.

### Problem-solving phase

Participants were asked to solve the same riddle during two different testing sessions within a limited time (10 and 4 min for the first and second testing session, respectively). The riddle, which has been used in a previous study^23^, states: “Zoe throws a stone that lands in the sky. How is it possible and in which context?”. The solution is that Zoe plays hopscotch, a well-known children game in which players must throw a stone into spaces drawn on the ground and then hop on them. The last winning space is traditionally represented by a half-circle named “sky” in France.

During the allotted time, the riddle remained displayed centrally on the screen. Participants were instructed to report all the ideas that came to mind, even if they judged them bizarre or irrelevant. They pressed the space button before expressing their response. Pressing the space bar stopped the timer, leaving the participants up to 30 s to type their response using the keyboard. The number of ideas that a participant could propose was not limited, and no feedback on the response accuracy was provided. For each response they gave, we asked them to assess the confidence they had in this response on a visual scale from 0 (“not sure at all”) to 100 (“completely sure”). Then, they indicated whether this response came to mind with a Eureka feeling by pressing the “yes” or “no” button^113^. The Eureka feeling was described during the instructions as “the subjective experience you may have when you solve a problem, and the solution comes to mind suddenly, is not the direct result of a cognitive effort, and you are not able to report the mental steps leading to this solution”. It was opposed to analytic solving where individuals can explain the internal steps and mental operations leading to the solution. In addition, we explained that these two solving methods were not exclusive and instructed participants to only consider the few seconds before the idea came to their mind. The riddle was displayed again once the participants replied to the Eureka question, and the timer started again. Throughout the sessions, we regularly probed (once every 2 min in the first session, and once every minute in the second session) participants to report their attentional focus. They answered the probes by choosing between four options (“Focused on the riddle”, “Distracted by the environment”, “Thoughts unrelated to the riddle”, “No thoughts”).

In the first solving phase, all participants were included in the analyses (*n* = 111, no participants were already familiar with the riddle). In the second solving phase, we did not consider participants who had already found the solution to the riddle during the first solving phase (*n* = 25), and 84 participants were included in the second solving phase analyses (Wake: *n* = 20; NREM: *n* = 20; REM: *n* = 44) (Supplementary Fig. 1).

### Relatedness judgment task (RJT)

During the experiment, participants performed the RJT before and after the incubation period. Participants were presented with all possible combinations of word pairs using a pre-established word list. This word list was composed of 20 problem-related words that were associated with the solution or were neutral. This material was developed and validated in a previous study^23^ and is available in Supplementary Table 1. On each trial, one of the 190 potential word pairs was displayed on the screen above a visual scale (Fig. 1B). Participants were asked to rate the strength of association between the two words on a visual scale from 0 (“unrelated”) to 100 (“strongly related”), using a slider. Participants had up to 4.5 s to respond using the computer mouse and validate their rating with a left-click. The order of trials was initially pseudo-randomized and then fixed across subjects and testing sessions. We used the Mix software^114^ to generate a pseudo-random order where each word appeared equally on the right and left sides of the screen and did not repeat in two consecutive trials. Before starting the task, the participants were instructed to answer as quickly and spontaneously as possible and completed 25 practice trials. Before the first RJT, all the words that composed the pairs (*n* = 20) were successively displayed on the screen. After they performed the RJT, we asked the participants whether they thought of a new response to the riddle during the RJT to ensure that participants did not solve the riddle during the RJT.

In addition to removing participants who solved the riddle in the first solving phase (*n* = 25) and those who fall asleep in the Wake group (*n* = 2), we excluded 19 additional participants for several reasons related to the RJT (Supplementary Fig. 1). First, participants who found the solution during the second RJT (*n* = 3) were excluded. We chose to exclude them because finding the solution during the RJT could have influenced subsequent ratings. Second, two additional participants were excluded due to technical issues during the RJT. Finally, we excluded participants based on their RJT completion quality. Individuals with more than 15% of non-responses (i.e., no response within the allotted time) and/or ratings of zero in the first and/or second RJT were excluded (*n* = 14, 11 for inflated proportion of zeros, two for inflated proportion of non-responses, and one for both). This threshold was adopted as a pragmatic quality-control criterion rather than as a formal statistical outlier cutoff. This exclusion was motivated by three considerations: (1) zero ratings and non-responses are inherently ambiguous and may reflect disengagement rather than true absence of associations, (2) in a longitudinal design, inflated number of zero ratings or non-responses at either time point can systematically distort the detection of true within-subject changes in semantic structure, and (3) zero ratings have a disproportionate impact on SemNet metrics relative to any other response values (see Supplementary Note 8). As a sensitivity analysis, we additionally examined unusually high proportions of zero ratings using a robust outlier approach based on the median absolute deviation (MAD), computed separately for pre-test and post-test. Specifically, modified z-scores were calculated as *M*_i_ = 0.6745(x_i_−median)/MAD, with the conventional threshold of 3.5 used to flag extreme cases. The constant 0.6745 scales the MAD-based score to be comparable to a standard z-score under normality. Zero-rating proportions were strongly right-skewed and concentrated near zero; the median was 1.32% at pre-test and 0.79% at post-test, with corresponding adjusted MAD values of 1.95 and 1.17. These robust dispersion estimates implied substantially lower data-driven thresholds than the prespecified 15% criterion, indicating that a MAD-based rule would be overly stringent for these bounded and zero-inflated data. We therefore retained the 15% rule as the primary exclusion criterion. Although arbitrary, this threshold effectively identifies participants whose response patterns deviate substantially from the sample distribution (see data distribution on Supplementary Fig. 8) and whose inclusion would risk compromising network stability and interpretability while avoiding overly aggressive participant removal. The final sample used for SemNet analyses included 65 participants (wake: *n* = 17; NREM: *n* = 17; REM: *n* = 31).

### Semantic memory network procedure

#### Semantic memory network estimation

For each participant, we built an individual SemNet before (preSemNet) and after (postSemNet) the incubation period, based on the corresponding preRJT and postRJT, respectively (Fig. 1A). These SemNets represent the organization of problem-related terms, (i.e., the 20 words of the RJT list, that could be solution-relevant or irrelevant) as nodes, with edges representing the relatedness between them (i.e., the 190 RJT ratings) (Fig. 1B). Hence, SemNets are represented as a 20 × 20 adjacency matrix (one word per row and column) containing all values of the individual RJT ratings. Based on previous studies using this approach^23,85,86,89,90,115^, we applied a weighted undirected network (WUN) thresholding approach. In these estimated WUNs, the relation between node *a* and node *b* is equal to the relationship between node b and node a (i.e., a symmetrical adjacency matrix). All edges are kept in the network, and edges are weighted based on the judgments provided by each participant during the RJT, without any transformation. The benefit of this methodology is that it avoids any arbitrary thresholding of edges for network filtering. Remaining non-responses in the RJT were substituted with a zero for metrics that could not be computed with missing values.

#### Semantic memory network metric computation

For each SemNet, we computed several metrics that quantify the connectivity properties of the network using the Brain Connectivity Toolbox (version 2019-03-03)^95^ running with Matlab R2020. They included: (1) *weight*, which is the raw strength of association extracted from each word pair judgment in the RJT (the higher the *weight*, the more semantically associated the words are); (2) *efficiency*, which is the inverse shortest path length representing the degree to which information can be efficiently exchanged between two nodes (the higher the *efficiency*, the shorter the path between two words is); (3) *clustering coefficient*, which represents the degree to which neighbor of a node are also connected to one another (the higher the *clustering coefficient*, the more interconnected words are); and (4) *eigenvector centrality*, a self-referential measure of centrality where nodes have a higher eigenvector centrality if they connect to other nodes which themselves have a high centrality (the higher the *eigenvector centrality*, the more central the word is) (Fig. 1C). We used these SemNet metrics because they have been commonly used in cognitive research^83,95,116–118^ and creativity research^86,89,90,92,94^. We selected them because they showed significant variations depending on whether individuals solved a problem or not in our previous study that used a similar approach^23^, and because they quantified distinct aspects of SemNets connectivity properties.

We computed the difference between pre- and post-SemNet metrics to measure changes in the properties of SemNets that could be explained by the incubation period. For each metric, we calculated an individual difference between the post-SemNet and pre-SemNet (ΔMetric = Metric_postSemNet_ – Metric_preSemNet_): the higher the ΔMetric, the larger the increase in the considered metric after the incubation period (Fig. 1C).

### Neurophysiological recording

#### Polysomnography

Participants’ sleep/wake states were monitored with a video-polysomnography during the whole experiment. The EEG montage included 10 electrodes on the scalp (F1, F2, C1, C2, P1, P2, O1, O2, T7, and T8, in the 10–20 EEG layout), two electrodes positioned above the right superior canthus and the left inferior canthus to record eye movements (EOG1/EOG2), and one electrode placed on the chin to measure muscle tone (EMG) (Compumedics Ltd, Victoria, Australia). Electrode impedances were below five kOhm. EEG signal was referenced to the controlateral mastoid (A1 or A2) and sampled at 250 Hz.

#### Sleep scoring

For sleep scoring, a 0.3–35 Hz filter was applied for EEG and EOG channels and a 10–100 Hz filter was applied for the EMG channel. For each participant, one experienced scorer (DO) allocated a sleep stage (Wake, N1, N2, N3, or REM sleep) to each 30-s epoch of the incubation period according to the standard sleep scoring guidelines from the American Academy of Sleep Medicine^96^. The sleep scorer was blind to the participant’s performance (RJT scoring and solving success).

#### EEG preprocessing

We excluded EEG recordings of participants in the Wake group because they did a jigsaw puzzle, leading to substantial noise in the EEG signal.

We filtered EEG signal using a notch filter at 50 Hz and at the main frequency harmonics (100 and 150 Hz) to remove power line noise. Signals were band-pass filtered between 0.3 and 40 Hz using sixth-order Butterworth filters. To identify bad channels, we computed the standard deviation (std) and kurtosis of each channel, then applied a logarithmic transformation to these values. We calculated the mean and averaged std of the log-transformed values across channels. The rejection thresholds were defined as follows:

$${{{{\rm{thresh}}}}}_{{{{\rm{std}}}}}={{{\rm{av}}}}{{{{\rm{g}}}}}_{{{{\rm{std}}}}}\pm 3{{{\rm{xav}}}}{{{{\rm{g}}}}}_{{{{\rm{std}}}}}$$

$${{{{\rm{thresh}}}}}_{{{{\rm{kurt}}}}}={{{\rm{av}}}}{{{{\rm{g}}}}}_{{{{\rm{kurt}}}}}\pm 3{{{\rm{xav}}}}{{{{\rm{g}}}}}_{{{{\rm{kurt}}}}}$$

where ${{{\rm{avg}}}}\_{{{\rm{std}}}}$ corresponds to the averaged log-transformed std across channels, and ${{{\rm{av}}}}{{{{\rm{g}}}}}_{{{{\rm{kurt}}}}}$ corresponds to the averaged log-transformed kurtosis across channels.

We rejected channels for which the std or kurtosis exceeded one of the defined thresholds (thresh_std_ and thresh_kurt_). Bad channels were then interpolated using neighboring channel data.

Recordings were epoched in 30-s windows. We defined sleep onset as the first N1 sleep epoch and sleep offset as the last sleep NREM or REM epoch preceding wake. Bad segments (i.e., any epoch containing at least 3 EEG channels exceeding ±150 µV) were excluded from further analysis.

#### EEG measures of cognitive richness

To examine SemNet changes based on oscillatory activity during the nap, we extracted a range of EEG measures across individual sleep epochs. They included a measure of the signal complexity (permutation entropy in theta frequency band—PE_θ_), the oscillatory component of the spectral power in the classical frequency bands (delta, theta, alpha, and beta power), as well as an aperiodic component (1/f spectral slope—Slope_1/f_). These markers have been shown to distinguish between conscious individuals, patients in a minimally conscious state, and those with unresponsive wakefulness syndrome^49,97^. They can also differentiate sleep/wake stages^52,119^ and capture changes in consciousness linked to meditation^120^ or variations in responsiveness to stimuli during sleep^52^. As such, they are believed to reflect varying levels of cognitive richness^49^. As restructuring supposed associative processes that could be facilitated by hyperassociativity state during REM, we speculated that REM sleep-related restructuring would be associated with a richer cognitive state, characterized by a higher PE_θ_ and EEG power in high-frequency bands (theta, alpha, beta), a flattening of the Slope_1/f_, and a lower power in low-frequency bands (delta).

EEG measures were computed for each electrode and each sleep stage independently. We computed the spectral power in the delta (0.5–4 Hz), theta (4–8 Hz), alpha (8–12 Hz) and beta (12–30 Hz) bands using Welch’s method on 5-s hamming windows sliding by 0.5 s. We chose these spectral frequencies because these markers have been robustly associated with changes in consciousness states in the consciousness literature^49,52^. We finally used the relative power for each frequency band computed by dividing the absolute power in the band by the total power across all bands for each electrode. We counteracted the 1/f activity of the spectrum by subtracting the aperiodic component (Slope_1/f_) from the absolute power values of each channel using the FOOOF package. The Slope_1/f_ value was retrieved as an individual EEG measure. Finally, we extracted the permutation entropy in the theta band (PE_θ_) on each 30-s epoch using the NICE toolbox. We focused on the theta band because its frequency range is mainly observed during REM sleep. All EEG measures were average across electrodes for each individual.

### Statistical and reproducibility

#### Group effect on problem-solving

For both solving phases, we computed the solving rate as the percentage of participants who gave the correct solution over the total number of participants. In addition, for the second solving phase, we computed the solving rate independently for each group (Wake [*n* = 20], NREM [*n* = 20], and REM [*n* = 44] groups). We explored whether sleep improved creative problem-solving success by testing whether the solving rate was group-dependent using a Fischer’s exact test.

Additional analyses were conducted to investigate whether participants reported an insight by using the Eureka reports, as well as response confidence ratings for correct and incorrect responses. Individual attentional level during the solving phases were also assessed using the probes. Methods and results related to these analyses are provided in the Supplementary Note 1.

#### Group effect on restructuring

We used analyses of variance (ANOVA) with participants entered as a random effect. All mixed models were run on Rstudio (v 1.4.1717) with aov function. The low solving rate after incubation did not allow us to use solving success (yes or no) as the dependent variable. Hence, △Metric was always the dependent variable (a continuous variable that represents SemNet changes of each node or edge) and was z-scored across all participants. We used as independent variables the Group (categorical variable with three levels: Wake [*n* = 17], NREM [*n* = 17], REM [*n* = 31]), and *semantic distance* (SD, continuous variable). The *semantic distance* variable was computed as in our previous study^23^. It measured the semantic distance of problem-related word pairs in SemNets built in a normative condition without the context of the problem. The higher the *semantic distance*, the most semantically remote a pair is or the most semantically isolated a word is. This normative condition correspond to the RJT ratings of 193 participants before the presentation of the Zoe riddle (see ref. ^23^).

We ran the same model independently for each network metric that we computed (*weight*, *efficiency*, *clustering coefficient*, and *eigenvector centrality*). Results were corrected for multiple comparisons using the Bonferroni method (significance threshold for *p* values fixed at 0.05/4, i.e., 0.0125). *Semantic distance* variable was z-scored across the whole group. The model can be formalized as follow:

1 $$\Delta {{{\rm{Metric}}}}={{{{\mathbf{\beta }}}}}_{{{{\bf{1}}}}}{{{\rm{x}}}}\; {{{\rm{Group}}}}+{{{{\mathbf{\beta }}}}}_{{{{\bf{2}}}}}{{{\rm{x}}}}\; {{{\rm{SD}}}}+{{{{\mathbf{\beta }}}}}_{{{{\bf{3}}}}}{{\rm{x}}}({{{\rm{Group}}}}\; {{{\rm{x}}}}\; {{{\rm{SD}}}})+{{{\rm{Error}}}}({{{\rm{Subject}}}})$$

This model accounts for several factors that could influence SemNet changes (i.e., ΔMetric). The Group factor alone assessed whether sleep was associated with averaged changes in SemNet (i.e., differences in averaged ΔMetric across edges/nodes at the individual level). The Group × semantic distance interaction factor assessed whether sleep was associated with SemNet changes targeting the most semantically remote problem-related concepts.

Significant ANOVA fixed effects were investigated with post hoc tests. For this purpose, we used the same model, comparing the Group factor effect pairwise. All *p* value reported in post hoc tests were corrected for multiple comparisons using the Bonferroni method.

We ran additional mixed-effects ANOVA assessing whether REM sleep would reduce the strength of the semantic associations related to the dominant, but solution-irrelevant, problem representation. In this model, we tested whether the SemNet changes (ΔMetric) differed as a function of the group (Wake [*n* = 17], NREM [*n* = 17], and REM [*n* = 17] group) and the type of nodes or edges (isDominant, categorical variable with two levels: related or unrelated to the dominant representation). To define the dominant representation of the problem, we classified responses of participant from previous study^23^ into categories (Supplementary Table 2). We considered that the dominant representation of the problem was the most frequent (but incorrect) category of responses (see Supplementary Note 3, Supplementary Fig. 9). We ran the same model independently for each network metric that we computed (*weight*, *efficiency*, *clustering coefficient*, and *eigenvector centrality*). Results were corrected for multiple comparisons using the Bonferroni method (significance threshold for *p* values fixed at 0.05/4, i.e., 0.0125). The model can be formalized as follow:

2 $$\Delta {{{\rm{Metric}}}} = 	 {{{{\mathbf{\beta }}}}}_{{{{\bf{1}}}}}{{{\rm{x Group}}}}+{{{{\mathbf{\beta }}}}}_{{{{\bf{2}}}}}{{{\rm{x isDominant}}}}\\ 	 +{{{{\mathbf{\beta }}}}}_{{{{\bf{3}}}}}{{{\rm{x}}}}({{{\rm{Group x isDominant}}}})+{{{\rm{Error}}}}({{{\rm{Subject}}}})$$

As before, significant ANOVA fixed effects were investigated with post hoc tests, comparing the Group factor effect pairwise. All *p* values resulting from post hoc tests were corrected for multiple comparisons using Bonferroni method.

### EEG analyses

We used mixed-effects ANOVA with participants entered as a random effect to investigate whether SemNet changes (ΔMetric) differed as a function of *semantic distance* (SD), and EEG features (EEG). The model can be formalized as follow:

3 $$\Delta {{{\rm{Metric}}}}={{{{\mathbf{\beta }}}}}_{{{{\bf{1}}}}}{{{\rm{x}}}}\; {{{\rm{EEG}}}}+{{{{\mathbf{\beta }}}}}_{{{{\bf{2}}}}}{{{\rm{xSD}}}}+{{{{\mathbf{\beta }}}}}_{{{{\bf{3}}}}}{{{\rm{x}}}}({{{\rm{EEGxSD}}}})+{{{\rm{Error}}}}({{{\rm{Subject}}}})$$

We ran independently the same model for each EEG features (PE_θ_, delta/theta/alpha/beta power, and Slope_1/f_; *n* = 6) computed during REM [*n* = 17] and NREM [*n* = 48] (combining N1, N2 and N3 sleep stages), using the SemNet metrics (*weight*, *efficiency*, *eigenvector centrality*, and *clustering coefficient*; *n* = 4). We used FDR correction for multiple comparisons (6 by 2 by 4 = 48 tests in total).

### Reporting summary

Further information on research design is available in the Nature Portfolio Reporting Summary linked to this article.

## Supplementary information

Supplementary Information

Reporting Summary

Transparent Peer Review file

## Data Availability

The study reported in this article was not formally preregistered. Experimental material is available in the Supplementary Information associated with this article, and in the method of our previous article 23 and its public persistent repository (https://osf.io/pk9nc/?view_only=4c4432106624412391bb4f1016a79ac5)^121^. The data sets generated and/or analyzed during the current study, and the scripts written is available in a public persistent repository (https://osf.io/2apgj/?view_only=c4c15039b6b04a0aafb8e731e996f632)^122^. Data sharing will be anonymized and will not include participants’ personnal information.

## Code availability

We used open software and toolboxes available online: semantic networks metrics were computed using the Brain Connectivity Toolbox (BCT, version 2019-03-03)^95^, EEG analysis were performed with the FOOOF and NICE toolbox, and statistical analyses were done using Rstudio (v 1.4.1717).
