# Supplementary material for: REM sleep favors the restructuring of problem-related semantic associations
Source: Commun Biol. 2026 Jun 3;9:1171. doi: 10.1038/s42003-026-10354-1 (PMC13542217; doi:10.1038/s42003-026-10354-1)
Supplement: Supplementary file 1 [file 42003_2026_10354_MOESM1_ESM.pdf]

REM sleep favors the restructuring of problem-related semantic associations

Supplementary Information

Théophile Bieth[1,2], Nicolas Decat[3]*, Yoed N. Kenett[4]*, Marie Scuccimarra[1], Marcela Ovando-Tellez[1], Alizée Lopez-Persem[1], Célia Lacaux[1,3], Saoussen Ben Younes[1], Arthur Le Coz[1,3], Sarah Moreno-Rodriguez[1], Isabelle Arnulf[1,3], Emmanuelle Volle[1§] & Delphine Oudiette[1,3§]

Affiliation

[1] Sorbonne University, Institut du Cerveau - Paris Brain Institute -ICM-, Inserm, CNRS, AP-HP Hôpital de la Pitié-Salpêtrière, Paris, France

[2] Neurology department, Pitié-Salpêtrière Hospital, AP-HP, F-75013, Paris, France

[3] AP-HP, Pitié-Salpêtrière Hospital, Service des Pathologies du Sommeil, National Reference Center for Narcolepsy, Paris, France

[4] Faculty of Data and Decision Sciences, Technion – Israel Institute of Technology, Haifa 3200003 Israel

* These authors contributed equally

[§] These authors jointly supervised this work

Corresponding Author

Theophile Bieth – theo_bieth@hotmail.fr

Emmanuelle Volle - emmavolle@gmail.com

Delphine Oudiette - delphine.oudiette@gmail.com

Supplementary Note

Supplementary Note 1: Insight report, participant confidence and attentional focus during the solving phases.

Solving the riddle is associated with insight solving

We explored whether successful problem-solving was associated an insight report (i.e., Eureka) using a chi-square analysis (corrected with Yates method when needed). For this test, we used all the participants pooled together (without considering their groups), excluding missing values. Indeed, the low solving rate in the second solving phase did not allow us to explore whether the participants' group influenced the frequency of Eureka. The analysis was repeated independently in each solving phase.

The proportion of Eureka was significantly higher for correct responses than incorrect ones, both in the first (56.52% and 19.37% respectively, $\chi^2(1) = 16.62$, $p = 4.58 \times 10^{-5}$) and the second solving phase (77.78% and 20.15% respectively, $\chi^2(1) = 13.65$, $p = 2.20 \times 10^{-4}$). This finding is consistent with our previous study [1] in which solvers reported a Eureka feeling more frequently than non-solvers. This result suggests that our riddle was related to an insight problem-solving and creative cognition.

Solvers are confident in their responses

We compared the averaged confidence across participants between correct and incorrect responses using two-tailed paired-samples t-test analyses. As for the previous analysis, we did not consider the participants' group.

Participants were more confident in the appropriateness of their responses when their response was the correct solution than when it was not, both in the first (76.11, standard error of the mean, SEM = 28.49 and 37.05, SEM = 30.26, respectively; $t(719) = 6.10$, $p = 1.72 \times 10^{-9}$) and the second solving phase (79.35, SEM = 40.82 and 40.82, SEM = 29.93, respectively; $t(277) = 4$, $p = 8.16 \times 10^{-5}$).

Attentional focus during solving phases

For each attentional probe, we computed the proportion of participants who reported being "focused on the riddle", "distracted by the environment", "having riddle-unrelated thoughts", or "having no thoughts". Participants who found the solution to the riddle were excluded at the time point they wrote the correct responses as being confident in one's solution would potentially decreases the need to actively look for novel solutions, thus distracting participants away of the task. We averaged the proportion of participants who reported being focused on the riddle across the probes, and then compared these proportions between the Wake, NREM, and REM groups using a chi-square analyses (corrected with Yates method when needed).

In both solving phases, the averaged proportion of participants who reported being focused on finding solutions to the riddle remained above 50% (Supplementary Figure 2). This proportion was not significantly different than the proportion of participant reporting not being focused (i.e., "distracted by the environment", "having riddle-unrelated thoughts", or "having no thoughts") between the three groups during the first solving phase (Wake: 57%,

NREM: 74%, REM: 60%, $\chi^2(2) = 2.34$, $p = .31$), and the second solving phase (Wake: 59%, NREM: 75%, REM: 60%, $\chi^2(2) = 1.56$, $p = .46$). As all participants started the experiments in the same conditions (i.e., a partial sleep restriction), this result emphasizes that the attentional focus was not different in all groups.

Supplementary Note 2: Dominant representation of the problem and getting closer to the solution

Measuring how participants' representation of the problem was close to the dominant representation of the problem (isDominant variable)

Two independent judges (TB and MS), blind to the participant's group (sleep/wake, solver/non-solver), qualitatively rated the various solutions they proposed, classifying each solution in a generic category. The list of categories was first established independently by each judge after contemplating all the answers to the riddle given by all participants recruited in the current study and a previous one [1]. In total, 1235 answers from 160 participants were analyzed. First, the two judges shared their list of categories and agreed on a common list of 21 categories that appeared relevant to classify all the responses proposed by the participants. For example, the category "Gravity" includes responses related to the space (e.g., "Zoe is on the moon") and the category "Strength" includes responses implying that the stone is thrown with a lot of strength so that it can land in the sky (e.g., "Zoe has superhuman strength") (see Supplementary Table 2 that summarizes all the categories).

Then, the two judges independently classified each of the 1235 responses generated by the participants into one of these categories. A response could belong to several categories if the subject used several concepts. For example, "Zoe throws the stone so far that it got in orbit" can be labeled as "Strength" because Zoe uses strength to throw the stone, but also as "Gravity" because being in orbit refers to gravity laws. 92.6% of the responses shared the same category for the two judges. When the between-judges classification for a given response was discordant, each judge scored the response again independently. The remaining divergences between judges (n = 71, 5.5%) were discussed individually, and a consensus was reached for all except for 14 of them (1%). For these non-consensual responses, only the common category between the two judges was kept, or when they had no category in common, all categories from both judges were kept.

Based on this categorization, we defined the dominant representation of the problem and how close a response was to the solution (isDominant). We defined the dominant representation of the problem based on a previous independent sample of participants (n = 35, 213 answers) who failed to solve the same riddle [1]. In this study, individual SemNets were computed before and after the presentation of the problem using the same task and material as in the current study but without an incubation period, and with only one solving phase. We considered that the dominant representation of the problem was the most frequent (but incorrect) category of responses. For each of the 21 categories of responses, we computed the proportion of all the responses given by each participant that fell within this category. Then, we computed the *dominance score* as the averaged value of this latter proportion across participants. The higher the *dominance score*, the more a category is repeated during the solving phase (Supplementary Figure 9). The most frequently proposed solutions were related to the concept of gravity (gravity category), the idea that the stone was held by something in the sky (transportation category), or that Zoe was flying (flight category). Among the RJT word list, we defined nodes that were related (n = 7) and unrelated (n = 13) to this dominant representation based on their semantic similarity with the dominant representation of the problem (i.e., related to gravity, the sky, or Zoe flying; Supplementary Table 1), and the edges that were related (n = 21) or unrelated (n = 78) to the dominant representation as the ones that connected nodes respectively related or unrelated to the dominant representation. Edges

that connected nodes related and unrelated to the dominant representation (n = 91) were discarded from the analyses.

To verify that the edges and nodes that we defined as being related to the dominant representation of the problem were indeed dominant in the SemNets, we again used the independent sample of participants (n = 35) who failed to solve the same riddle in our previous study [1]. We tested whether our pre-selected dominant edges and nodes occupied a more important position in these SemNets relative to the non-dominant ones. We used ANOVAs considering participants as a random effect. In this model, we explored whether △Metric (dependent variable) differed depending on the node/edge dominance (i.e., related or not to the dominant representation of the problem). We ran a model for each computed metric (*weight*, *efficiency*, *clustering coefficient*, and *eigenvector centrality*) and each SemNet (naïve [before the presentation of the problem] and problem contextualized SemNet [after participants failed in finding the solution]).

Supporting our hypothesis, we found that the nodes and edges defined as related to the dominant representation of the problem were more semantically associated, more efficient, more interconnected, and more influent compared to others in both naive SemNet (*weight*: $F(1,2517) = 1210$, $p < 10^{-16}$; *efficiency*: $F(1,2547) = 1418$, $p < 10^{-16}$; *clustering coefficient*: $F(1,493) = 217.5$, $p < 10^{-16}$; *eigenvector centrality*: $F(1,493) = 351.5$, $p < 10^{-16}$) and problem-contextualized SemNet (*weight*: $F(1,2480) = 1387$, $p < 10^{-16}$; *efficiency*: $F(1,2547) = 1649$, $p < 10^{-16}$; *clustering coefficient*: $F(1,493) = 218$, $p < 10^{-16}$; *eigenvector centrality*: $F(1,493) = 355.7$, $p < 10^{-16}$).

Measuring how participants were getting closer to the solution (isGettingCloser variable)

Regarding the closeness to the solution, we considered close to the solution the participants' responses that were (i) related to another representation of the usual sky (i.e., response in the "Sky" category), (ii) related to a game (i.e., response in the "Game" category), or (iii) the correct solution (i.e., response in both the "Game" and "Sky" categories). All other responses were defined as far from the solution. A participant was considered as getting closer to the solution if they moved from a far response (neither in "Sky" nor "Game" category) in the first solving phase to a close response (either in "Sky" or "Game" category) or to the correct response (both in "Sky" and "Game" categories) in the second solving phase.

Even if the incubation period did not impact the solving success per se, we wondered whether it helped participants become one step closer to the solution compared to the first solving attempt pre-incubation. While the proportion of participants who got closer to the solution tended to be higher in the REM group, it did not reach the significance threshold (Wake: 20%, n = 4/20 solvers; NREM: 5%, n = 1/20 solvers; REM: 29.5%, n = 13/44 solvers; Fisher test, $p = .085$).

Measuring how REM sleep modulated semantic associations related to the dominant problem representation

We assessed whether REM sleep would reduce the strength of the semantic associations related to the dominant, but solution-irrelevant, problem representation. We tested whether the SemNet changes (△Metric) differed as a function of the group (REM, NREM, and Wake group) and the type of nodes or edges (isDominant, categorical variable with

2 levels: related or unrelated to the dominant representation). Here, we were particularly interested in the interaction effect of these two factors, which allowed us to assess whether sleep induced a SemNet changes that targeted nodes or edges related to the dominant representation. We expected that REM sleep could be associated with a lower representativity of edges and nodes related to the dominant representation (Group x isDominant interaction effect), fostering alternative problem representation.

We found a significant positive interaction effect between isDominant and Group on △Metric for *eigenvector centrality* ($F(2,1232) = 7.13$, $p = 8.34 \times 10^{-4}$, $\eta^2 = .01$) (Supplementary Figure 5). Post-hoc analyses revealed that the interaction effect was driven by a △Metric difference regarding the isDominant variable between the REM and Wake groups ($F(1,910) = 13.5$, $p = 2.52 \times 10^{-4}$, $\eta^2 = .01$), but not between NREM and REM groups ($F(1,910) = 3.07$, $p = .08$), nor between NREM and Wake groups ($F(1,644) = 3.37$, $p = .07$). These results indicate that nodes related to the dominant representation became less central (*eigenvector centrality* decreased) than those unrelated to it in the REM group compared to the Wake group. Such finding suggests that REM sleep decreases the centrality of concepts related to the dominant representation of the problem or increases the centrality of the non-dominant one. These results indicate that nodes related to the dominant representation became less central (*eigenvector centrality* decreased) than those unrelated to it in the REM group compared to the Wake group. Such finding suggests that REM sleep decreases the centrality of concepts related to the dominant representation of the problem or increases the centrality of the non-dominant one. However, the Group x isDominant interaction effect was not statistically significant for *weight* ($F(2,6019) = 2.28$, $p = .10$), *efficiency* ($F(2,6367) = .80$, $p = .45$), and *clustering coefficient* ($F(2,1232) = 1.19$, $p = .30$).

Supplementary Note 3: REM-sleep effect on global SemNet restructuring

We found a statistically significant main effect of Group on △Metric for *weight* ($F(2,59) = 3.37$, $p = .04$, $\eta^2 = .10$), *clustering coefficient* ($F(2, 62) = 3.26$, $p = .045$, $\eta^2 = .10$), and *eigenvector centrality* ($F(2,62) = 4.42$, $p = .016$, $\eta^2 = .12$) (Supplementary Figure 3). However, these results did not survive the correction for multiple comparisons. The main effect of Group was not statistically significant for *efficiency* ($F(2,62) = 1.87$, $p = .16$).

Supplementary Note 4: REM-sleep related SemNet changes did not result from a longer total sleep time in REM group.

To ensure that the REM sleep related SemNet changes we found was not due to a longer total sleep duration in the REM group compared to the NREM group, we ran additional control analyses. We investigated whether SemNet changes (△Metric) differed as a function of the total sleep duration (Sleep Duration, continuous independent variable) and the *semantic distance* (SD). We used similar statistical approach as before for the four metrics (weight, efficiency, clustering coefficient, and eigenvector centrality) with mixed-effects ANOVAs, that can be formalized as follow:

$$(4)\ \triangle\text{Metric} = \boldsymbol{\beta}_1 \times \text{Sleep Duration} + \boldsymbol{\beta}_2 \times \text{SD} + \boldsymbol{\beta}_3 \times (\text{Sleep Duration} \times \text{SD}) + \text{Error(Subject)}$$

We did not find a significant main effect of Sleep Duration on △Metric for the four metrics (*weight*: $F(1,44) = 1.88$, $p = .18$; *efficiency*: $F(1,46) = 1.07$, $p = .31$; *clustering coefficient*: $F(1,46) = 3.08$, $p = .086$; *eigenvector centrality*: $F(1,46) = .35$, $p = .56$). Additionally, we did not find a significant Sleep Duration and SD interaction effect on △Metric for the four metrics (*weight*: $F(1,8521) = .03$, $p = .86$; *efficiency*: $F(1,9070) = 1.50$, $p = .22$; *clustering coefficient*: $F(1,910) = .18$, $p = .68$; *eigenvector centrality*: $F(1,910) = .72$, $p = .40$).

These results suggest that the REM sleep-related SemNet changes observed were not driven by the difference in the total sleep duration between the REM and NREM sleep groups.

Supplementary Note 5: Bootstrapping to investigate the impact of the unbalanced sample size of the three groups.

As our main question concerned the REM sleep stage, we maximized the chance of obtaining participants who would reach REM sleep in the sleep group by asking them to reduce their sleeping time the night before and come early in the morning (7:00 am) when REM sleep pressure is higher. Surprisingly, we observed a proportion of REM sleep in the Sleep group larger than expected (about 2 participants with REM sleep for 1 with NREM sleep only). Increasing the sample size would significantly alter this distribution and recruiting participants during an afternoon nap (to maximize NREM sleep over REM sleep) would introduce potential circadian confounds.

To consider this factor that may influence our statistical analyses, we proposed additional control analyses. We randomly and iteratively subsampled the REM group to make the three groups equivalent in terms of sample size (n = 17). For each REM group resample (n = 500), we ran our statistical analysis (Model 1) and kept the *p* value related to the Group x semantic distance interaction effect. We plotted the distributions of these *p* value for each metric, with whom we found a statistically significant results with the full sample (Figure 2).

The proportion of significant *p* value (i.e., < .05) for the interaction effect remains superior to 95%, suggesting that our findings was not influenced by the unbalanced sample size (Supplementary Figure 4).

Supplementary Note 6: NREM sleep-related SemNet changes at the neural level.

Using mixed-effects ANOVA, we investigated whether SemNet changes (ΔMetric) differed as a function of semantic distance, and EEG features computed separately in N1, N2 and N3 sleep stage. We used the same EEG markers as the main analysis (PE_{θ}, delta/theta/alpha/beta power, and $Slope_{1/f}$) and applied an FDR correction for multiple comparisons.

We observed different associations between EEG markers and remoteness-based SemNet changes according to the sleep sub-stages composing NREM sleep (Supplementary Figure 6A). First, during N1 sleep, we found a higher remoteness-based SemNet changes associated with a higher PE_{θ} (*weight*: F(1,8346) = 6.63, *p* = .01, η^2 = .001, Supplementary Figure 6B), a steeper $Slope_{1/f}$ (*weight*: F(1,8346) = 6.96, *p* = 8.36 x 10^{-3}, η^2 = .001; *efficiency*: F(1,8881) = 15.37, *p* = 8.91 x 10^{-5}, η^2 = .002, Supplementary Figure 6C; *eigenvector centrality*: F(1,891) = 24.62, *p* = 8.36 x 10^{-7}, η^2 = .03), and a lower power in delta frequency band (*weight*: F(1,8346) = 6.44, *p* = .01, η^2 = .001; *efficiency*: F(1,8881) = 6.17, *p* = .01, η^2 = .001, Supplementary Figure 6D).

Then, during N2 sleep, we found a higher remoteness-based SemNet changes associated with a steeper $Slope_{1/f}$ (*weight*: F(1,8339) = 8.12, *p* = 4.39 x 10^{-3}, η^2 = .001; *efficiency*: F(1,8881) = 16.95, *p* = 3.87 x 10^{-5}, η^2 = .002, Supplementary Figure 6E; *eigenvector centrality*: F(1,891) = 6.74, *p* = 8.42 x 10^{-3}, η^2 = .008), a lower power in delta frequency band (*eigenvector centrality*: F(1,891) = 7.88, *p* = 5.11 x 10^{-3}, η^2 = .009, Supplementary Figure 6F), and a higher power in alpha frequency band (*efficiency*: F(1,8881) = 10.20, *p* = 1.41 x 10^{-3}, η^2 = .001, Supplementary Figure 6G; *eigenvector centrality*: F(1,891) = 10.10, *p* = 1.53 x 10^{-3}, η^2 = .01).

Finally, during N3 sleep, we found a higher remoteness-based SemNet changes associated with a lower PE_{θ} (*weight*: F(1,4702) = 8.10, *p* = 4.44 x 10^{-3}, η^2 = .002, Supplementary Figure 6H), a lower power in beta frequency band (*weight*: F(1,4702) = 27.28, *p* = 1.84 x 10^{-7}, η^2 = .006; *efficiency*: F(1,5101) = 11.78, *p* = 6.05 x 10^{-4}, η^2 = .002, Supplementary Figure 6I; *clustering coefficient*: F(1,511) = 9.01, *p* = 2.81 x 10^{-3}, η^2 = .02), a less steep $Slope_{1/f}$ (*efficiency*: F(1,5101) = 7.04, *p* = 7.99 x 10^{-3}, η^2 = .001; *eigenvector centrality*: F(1,511) = 13.95, *p* = 2.09 x 10^{-4}, η^2 = .03, Supplementary Figure 6J), and a higher power in delta frequency band (*weight*: F(1,4702) = 18.05, *p* = 2.20 x 10^{-5}, η^2 = .004).

Supplementary Note 7: Association between SemNet metric changes and successful problem-solving

In our princeps study [1], we found that restructuring – measured as SemNet metric changes – was associated with successful problem-solving. We proposed in the current study to use the SemNet methodology to explore the effect of an intervention (i.e., REM sleep) on restructuring. The current study's design differs fundamentally from our 2024 work (Supplementary Figure 7). Specifically: (1) the timing of SemNet computation differs between studies. In our 2024 paper, SemNets were computed before (pre-exposure) and after the solving phase, whereas in the current study, SemNets were computed immediately after the solving phase and again after the incubation period; (2) the current study lacks pre-problem SemNet assessment (baseline), preventing measurement of how SemNet change from baseline in response to problem exposure; and (3) an incubation period was introduced between SemNet assessments in the current design. These methodological differences make direct replication of our previous study challenging.

However, to explore the link between SemNet metrics and problem-solving success, we conducted two additional analyses. First, we examined whether SemNet metrics before the incubation period were associated with successful problem-solving during the first solving phase (i.e., before the incubation). If problem-solving is associated with changes in semantic remoteness within SemNet, we would expect differences in SemNet organization after the initial solving phase between solvers and non-solvers. After the first problem-solving phase, 23 participants solved the riddle whereas 65 did not. We used a logistic regression with individuals (Subject) as random factors to predict problem-solving success before the incubation (isSolved1) based on pre-incubation $SemNet_{pre}$ metric ($Metric_{Pre}$) and *semantic distance* (SD): isSolved1 = $\beta_0 + \beta_1$ x SD + β_2 x MetrciPre + β_3 x $Metric_{Pre}$ x SD + (-1 + $Metric_{Pre}$ | Subject). We found a statistically significant interaction effect between $SemNet_{pre}$ metric and *semantic distance* on problem-solving for *clustering coefficient* (β_3 = .44, p = .001). This suggests that semantically remote associations are more interconnected (higher *clustering coefficient*) in individuals who solved the riddle early (before the incubation) compared to those who did not. No such significant associations were observed for *weight* (β_3 = .04, p = .087), *efficiency* (β_3 = .04, p = .08), or *eigenvector centrality* (β_3 = .02, p = .71).

Second, we explored whether restructuring (measured with $\triangle$Metric) was associated with successful problem-solving, regardless of the type of incubation (i.e., the group: Wake, NREM, or REM). After the second solving phase, six participants solved the riddle whereas 59 did not. Again, we use a logistic regression with individuals (Subject) as random factor to predict problem-solving success after the incubation (isSolved2) based on SemNet metric changes ($\triangle$Metric) and *semantic distance* (SD): isSolved2 = $\beta_0 + \beta_1$ x SD + β_2 x $\triangle$Metric + β_3 x $\triangle$Metric x SD + (-1 + $\triangle$Metric | Subject). We found statistically significant interaction effects between $\triangle$Metric and *semantic distance* on problem-solving for both *efficiency* (β_3 = .10, p = .0046) and *eigenvector centrality* (β_3 = .21, p = .03). These interaction effects suggest that larger positive changes in *efficiency* and *eigenvector centrality* in semantically remote pairs/words were associated with a higher solving success rate. No such significant associations were observed for *weight* (β_3 = .006, p = .87) or *clustering coefficient* (β_3 = -.14, p = .53).

In summary, despite factors limiting the exact replication of our princeps results, we found several results that are nevertheless consistent with our primary findings. In addition, the current results confirmed that the SemNet metrics used can effectively capture variations

in SemNet organization. The only deviating result concerns the lack of significant effect for global SemNet changes for efficiency. This discrepancy may be due to the lower reliability of global-level SemNet metrics compared to local-level measures [2], or to the small size of the SemNets, which may lead to underpowered estimates at the SemNet global level. We believe that follow-up studies are needed to replicate our results and further examine this issue.

Supplementary Note 8: Exclusion of participants based on RJT completion.

For behavioral and EEG analyses that required SemNet metric, we excluded participants who had a cumulated proportion of zeros and/or non-responses (over the 190 ratings they had to do during the first and/or the second RJT) higher than 15% (n = 15, Supplementary Figure 1).

We chose to exclude participants with an inflated number of zero ratings and non-responses because the meaning behind is ambiguous. Zero ratings can reflect true semantic unrelatedness, meaning that the participant genuinely perceives the two words as completely unrelated, which would provide valid information about their mental semantic memory structure. However, zero ratings could be interpreted in several other ways: (i) they could indicate non-engagement or low effort, in which the participant selects zero indiscriminately or without carefully considering the word pair, perhaps due to fatigue, boredom, or misunderstanding of the task instructions. Of note, we specifically asked participants in the instruction before the RJT completion to use the entire visual scale to rating their relatedness judgment, and the 20 words was beforehand shown to the participant; (ii) they may be used as a substitute for "I don't know" or missing knowledge, when the participant cannot evaluate the pair as the task does not provide a specific dedicated option; (iii) they might reflect a systematic response bias, where the participant habitually overuses extreme low values, either as a personal response style or because of fatigue (e.g., skewing the participant's worldview as "black or white").

This is particularly relevant because zero ratings and non-responses have a large impact on the SemNet metric computation. Specifically, over-inflated zeros (i) reduce the density of the network, making it appear sparser than it truly is; (ii) lower the *clustering coefficient* by eliminating triangles of connections, giving the impression of weaker local coherence; (iii) increase path lengths and reduce global efficiency, making the network appear less integrated; and (iv) can artificially inflate modularity, suggesting mote compartmentalized substructures, and destabilize *eigenvector centrality*, making key nodes appear less important or central than they are. Additionally, if zeros are unevenly distributed or disproportionately present at one timepoint, the resulting differences may reflect response artifacts rather than true changes in semantic structure, undermining the validity of the longitudinal analysis. Therefore, carefully assessing zero is critical to ensure that observed changes over time reflect genuine cognitive differences rather than data noise.

The threshold we used is somewhat arbitrary. However, we provide in Supplementary Figure 8 the distribution of proportion of zeros and non-responses for each participant during the first and second RJT, indicating the 15% threshold. These graphs show that excluded participants largely exceeded the threshold. In the plots, we also indicated alternative thresholds (i.e., 10 and 20%, in dashed lines). We observed that the threshold choice did not drastically change the count of excluded participants.

Supplementary Figures

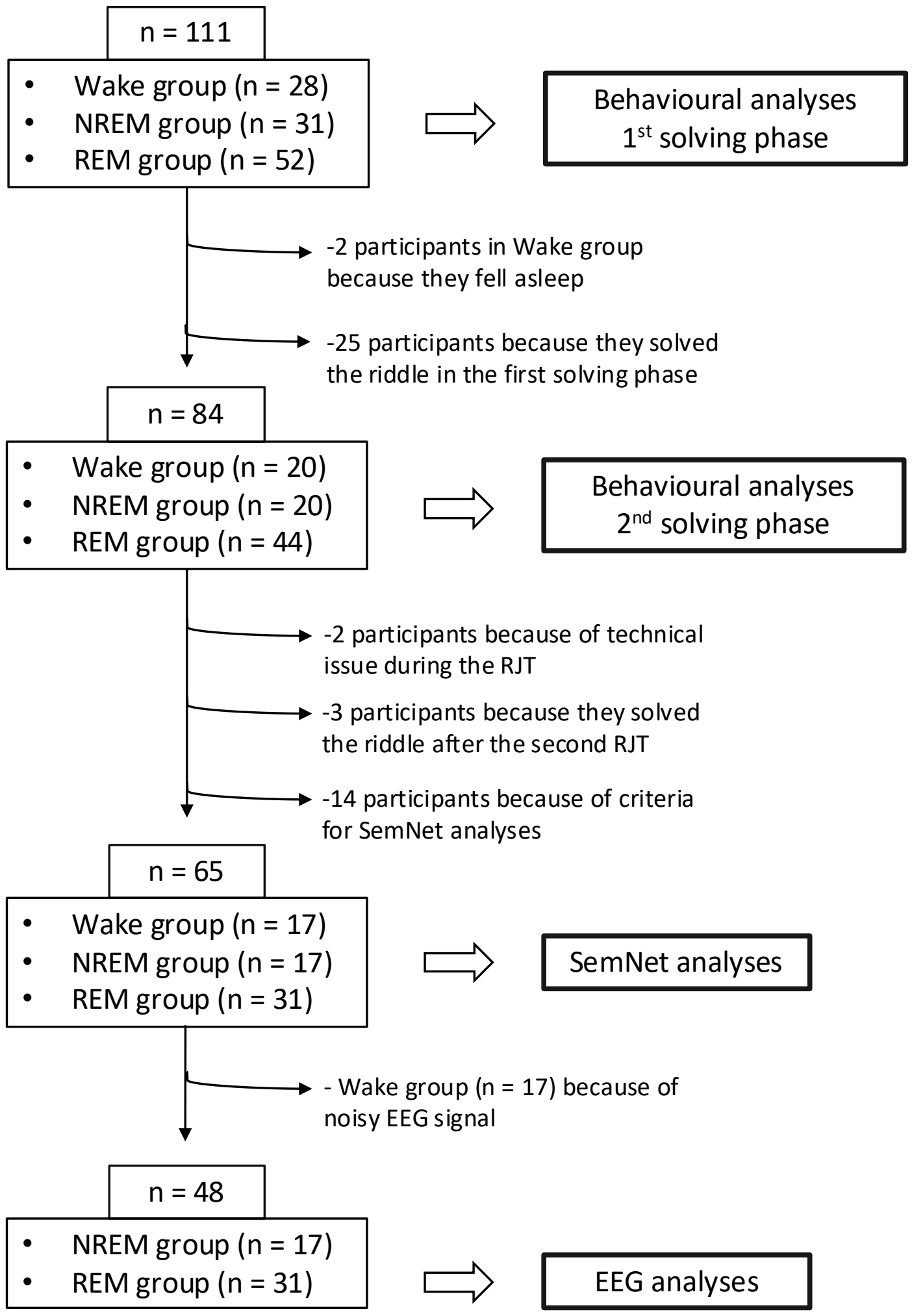

Supplementary Figure 1. Flow chart.
RJT – relatedness judgment task; SemNet – semantic network

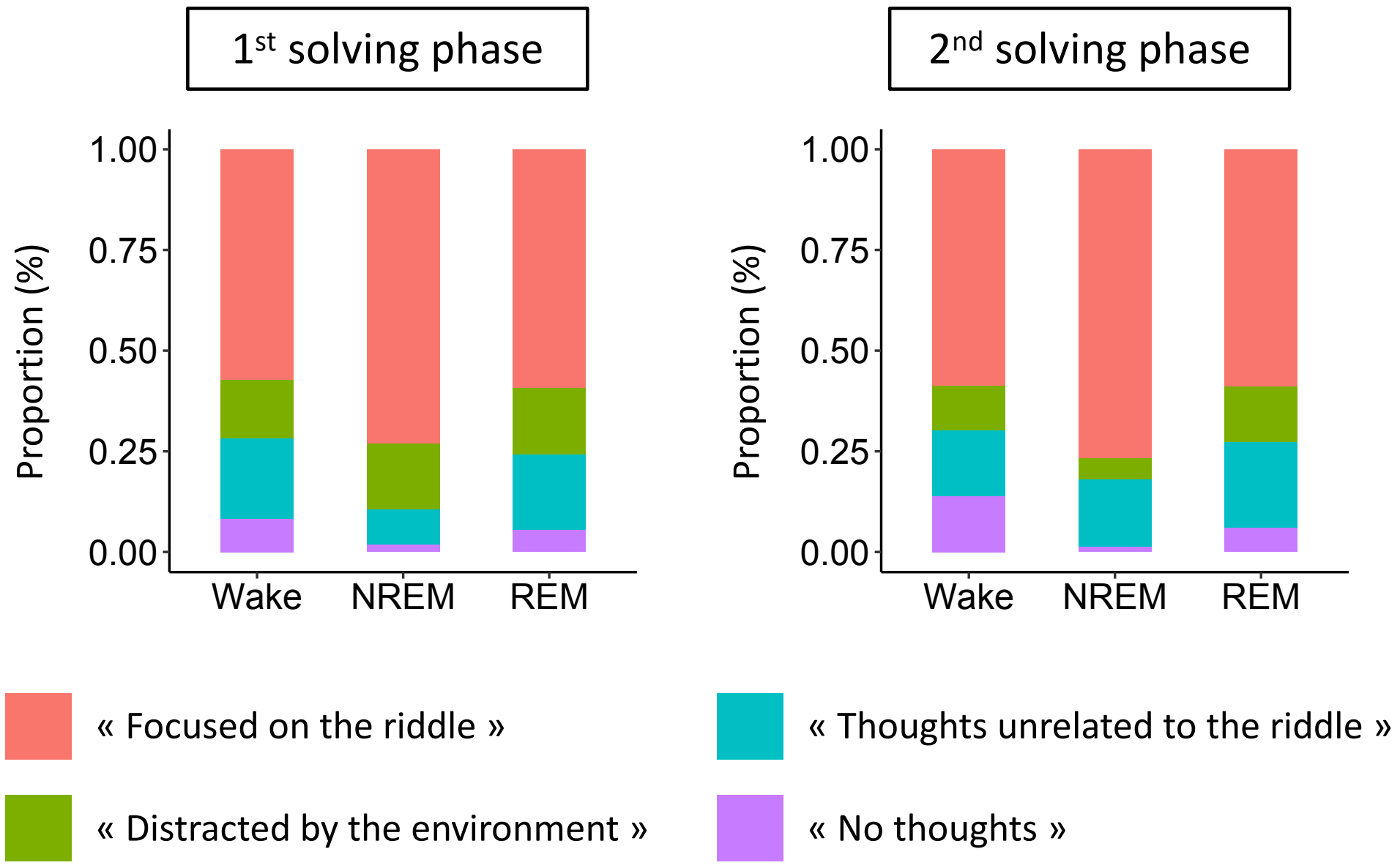

Supplementary Figure 2. Attentional focus during the solving phases.
Bar plots representing the averaged proportion of participants' responses to each attentional probe ("Focused on the riddle" in red, "Distracted by the environment" in green, "Thoughts unrelated to the riddle" in blue, and "No thought" in purple) across the probes (one every 2 minutes for the first solving phase, left part; one every minute for the second one, right part) for Wake (n = 28 and 20 for the first and second solving phase respectively), NREM (n = 31 and 30 for the first and second solving phase respectively), and REM (n = 52 and 44 for the first and second solving phase respectively) groups.

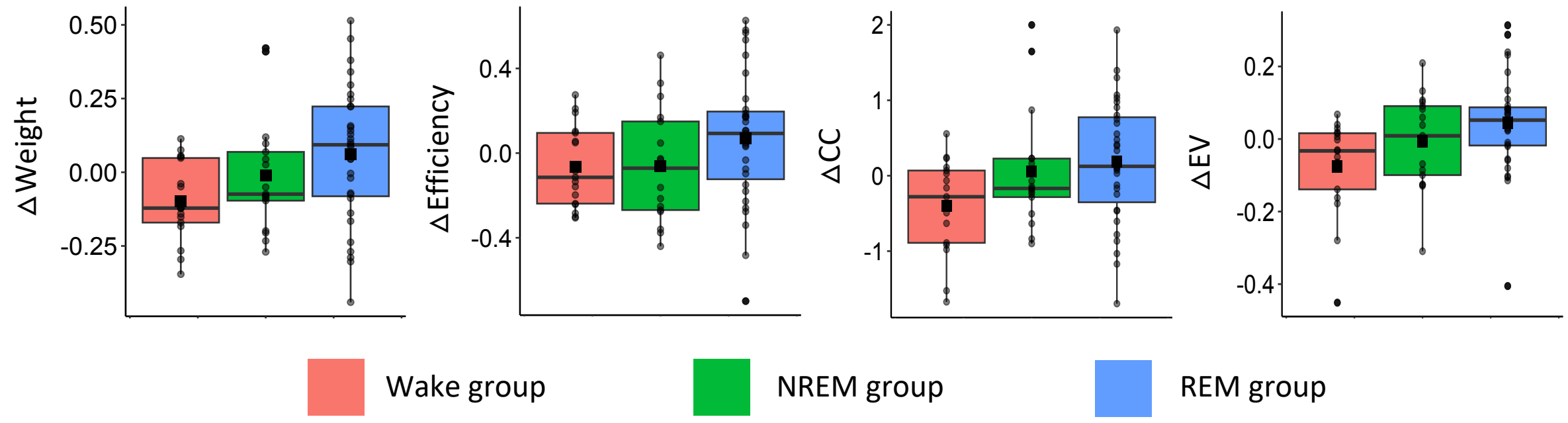

Supplementary Figure 3. REM sleep and global SemNet changes.
Averaged values of △Metric as a function of the group (Wake, n = 17; NREM, n = 17; and REM, n = 31) for the four metrics. Each dot represents a participant, color boxes represent the upper and lower quartiles, black lines and squares indicate respectively the median and the mean.

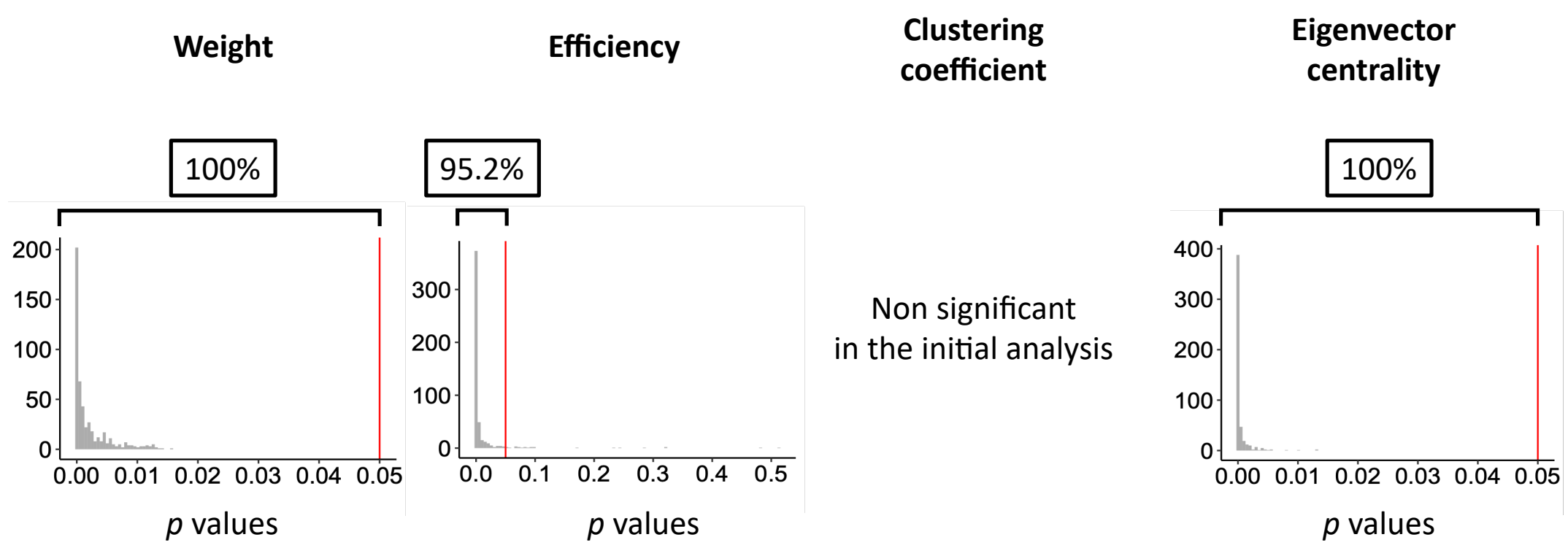

Supplementary Figure 4. Distribution of p values resulting from bootstrapping procedure. Distribution of the 500 *p* values resulting from a bootstrapping that resample the REM group sample size to be equivalent to the other sample size groups for the Group x *semantic distance* interaction effect. The bootstrapping procedure was not done for non statistically results with the unbalanced sample size. Red vertical line indicates the statistical threshold (i.e., .05). The proportion of *p* values over the 500 tests done that remained under the statistical threshold was specified.

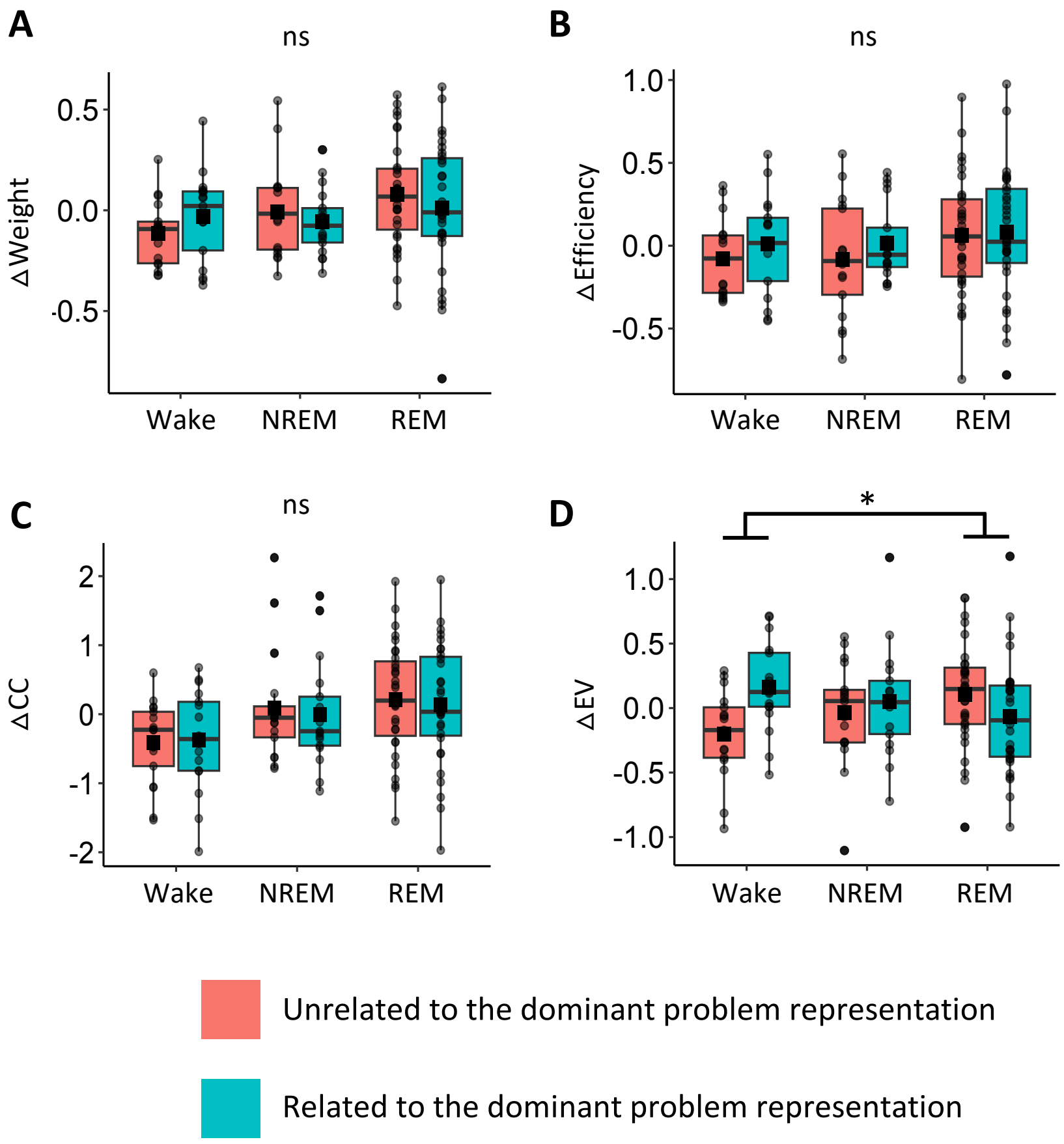

Supplementary Figure 5. REM sleep favors problem-related alternative associations over dominant ones. Changes in the semantic networks after the incubation period (△Metric) as a function of the group (Wake, n = 17; NREM, n = 17; and REM, n = 31) and of the type of nodes/edges (related or unrelated to the dominant representation in blue and red, respectively) for weight (A), efficiency (B), clustering coefficient - CC (C), and eigenvector centrality - EV (D). Each dot represents a participant, color boxes represent the upper and lower quartiles, and black lines and squares indicate respectively the median and mean.
*: $p < .05$, ns: non-statistically significant differences.

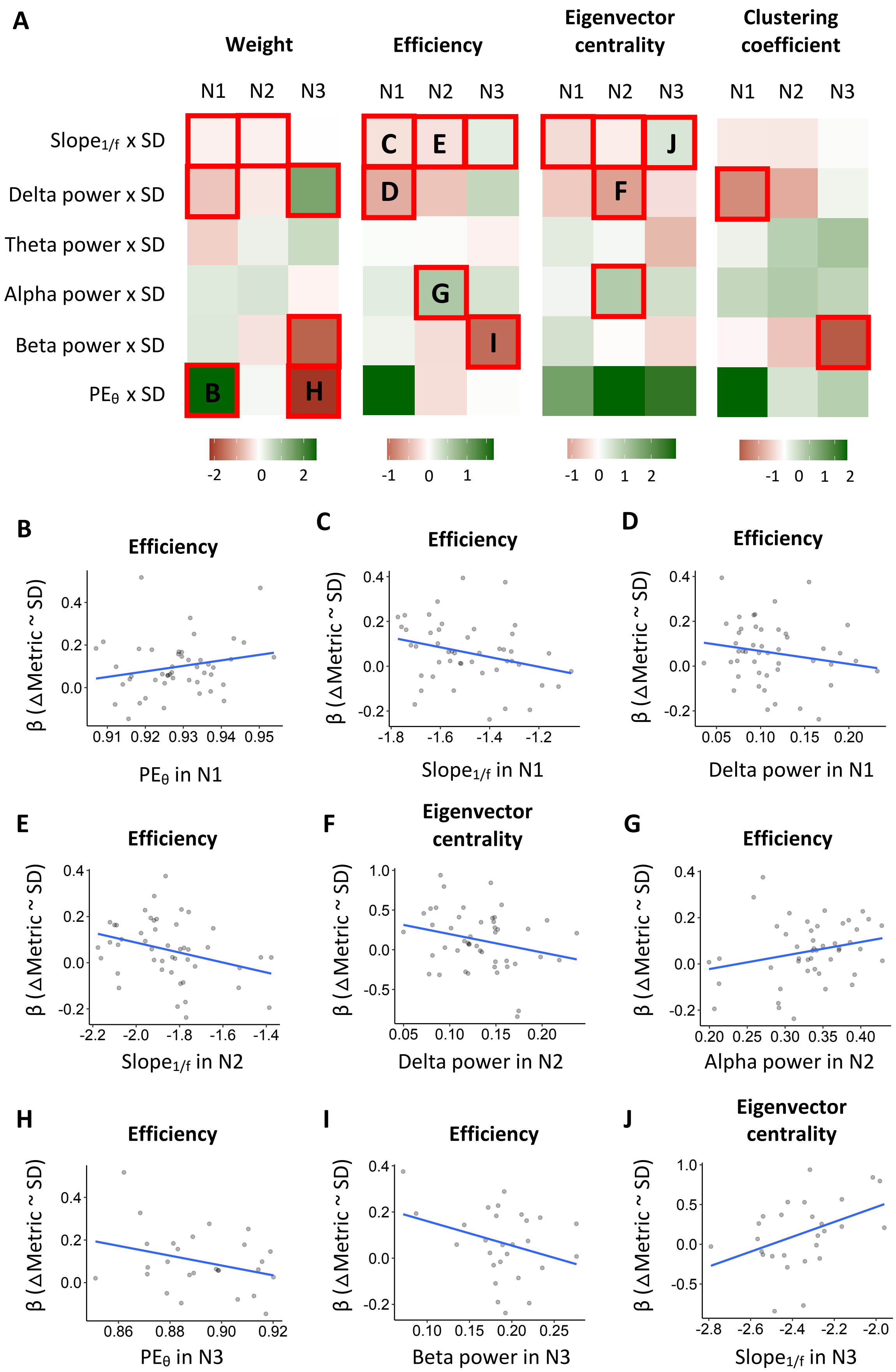

A
Weight
Efficiency
Eigenvector centrality
Clustering coefficient
N1 N2 N3
Slope1/f x SD
Delta power x SD
Theta power x SD
Alpha power x SD
Beta power x SD
PEθ x SD
C E J
D F
G
I
B H
-2 0 2
-1 0 1
-1 0 1 2
-1 0 1 2
B
Efficiency
β (ΔMetric ~ SD)
PEθ in N1
C
Efficiency
Slope1/f in N1
D
Efficiency
Delta power in N1
E
Efficiency
Slope1/f in N2
F
Eigenvector centrality
Delta power in N2
G
Efficiency
Alpha power in N2
H
Efficiency
PEθ in N3
I
Efficiency
Beta power in N3
J
Eigenvector centrality
Slope1/f in N3

Supplementary Figure 6. EEG measures associated with semantic-based SemNet changes differ within NREM sleep.

Heatmaps (A) represent statistical results for each EEG markers (rows) computed in N1 (n = 47), N2 (n = 47) and N3 (n = 27) sleep stages (columns) for the four metrics. The color code indicates the regression coefficients of the EEG x *semantic distance* (SD) interaction factor, ranging from red (negative values) to green (positive values). Squares framed in red indicate significant *p* values (i.e., < .05 after FDR correction). Letters inside the red-framed squares correspond to the figure panels that provide additional graphical representations of these results (from B to J), showing the regression coefficient between △Metric and SD as a function of an EEG marker with each dot representing an individual participant.

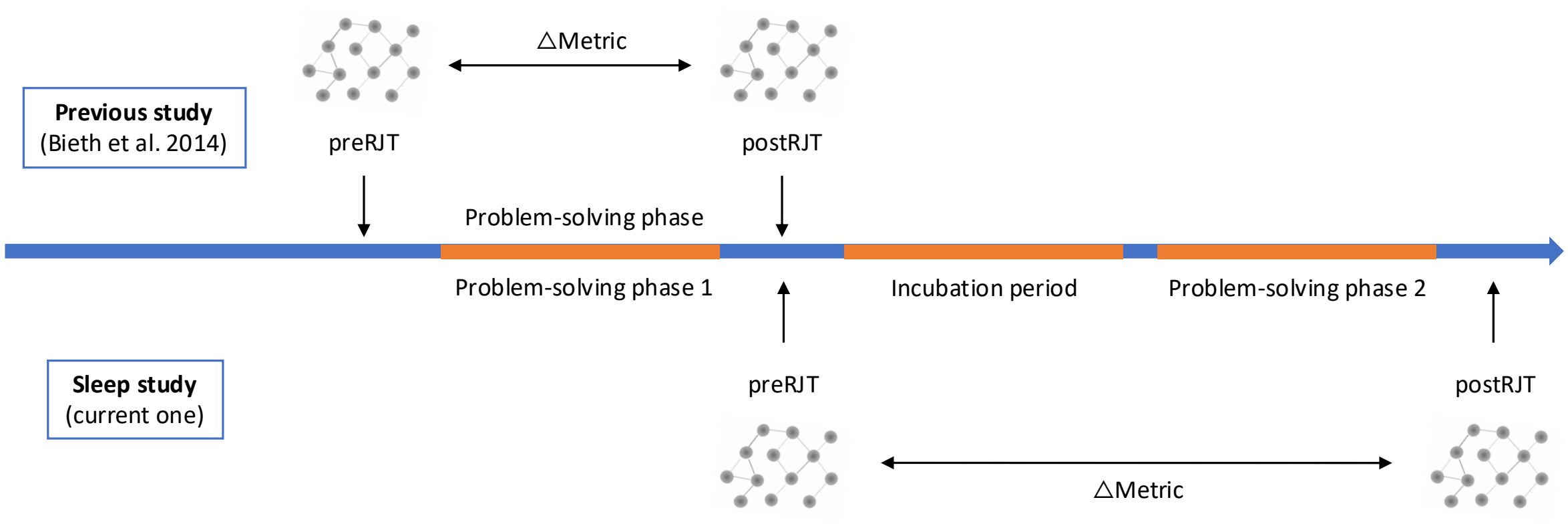

Supplementary Figure 7. Experimental design differences between our previous study [1] and the current one.

A **First RJT**

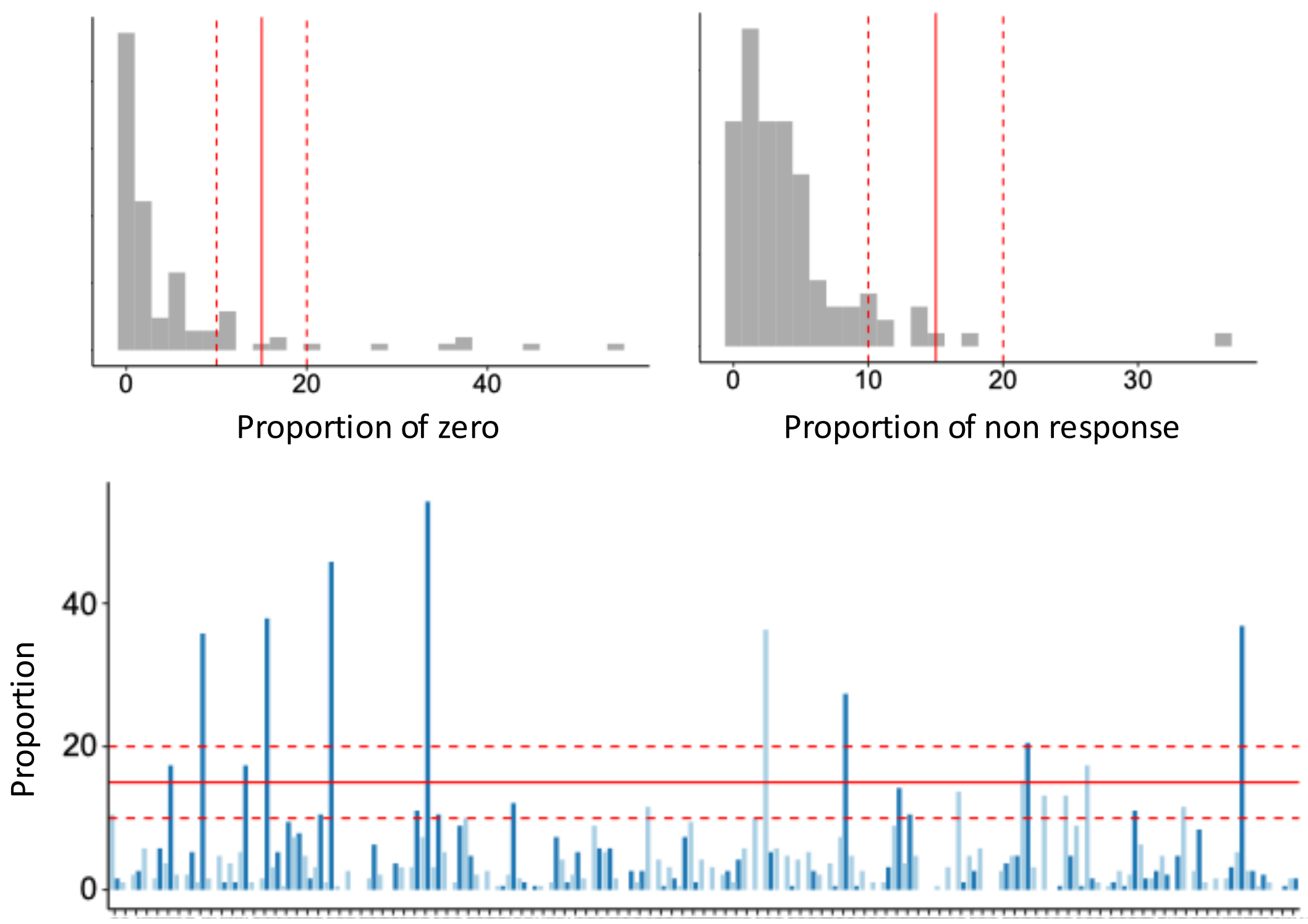

B **Second RJT**

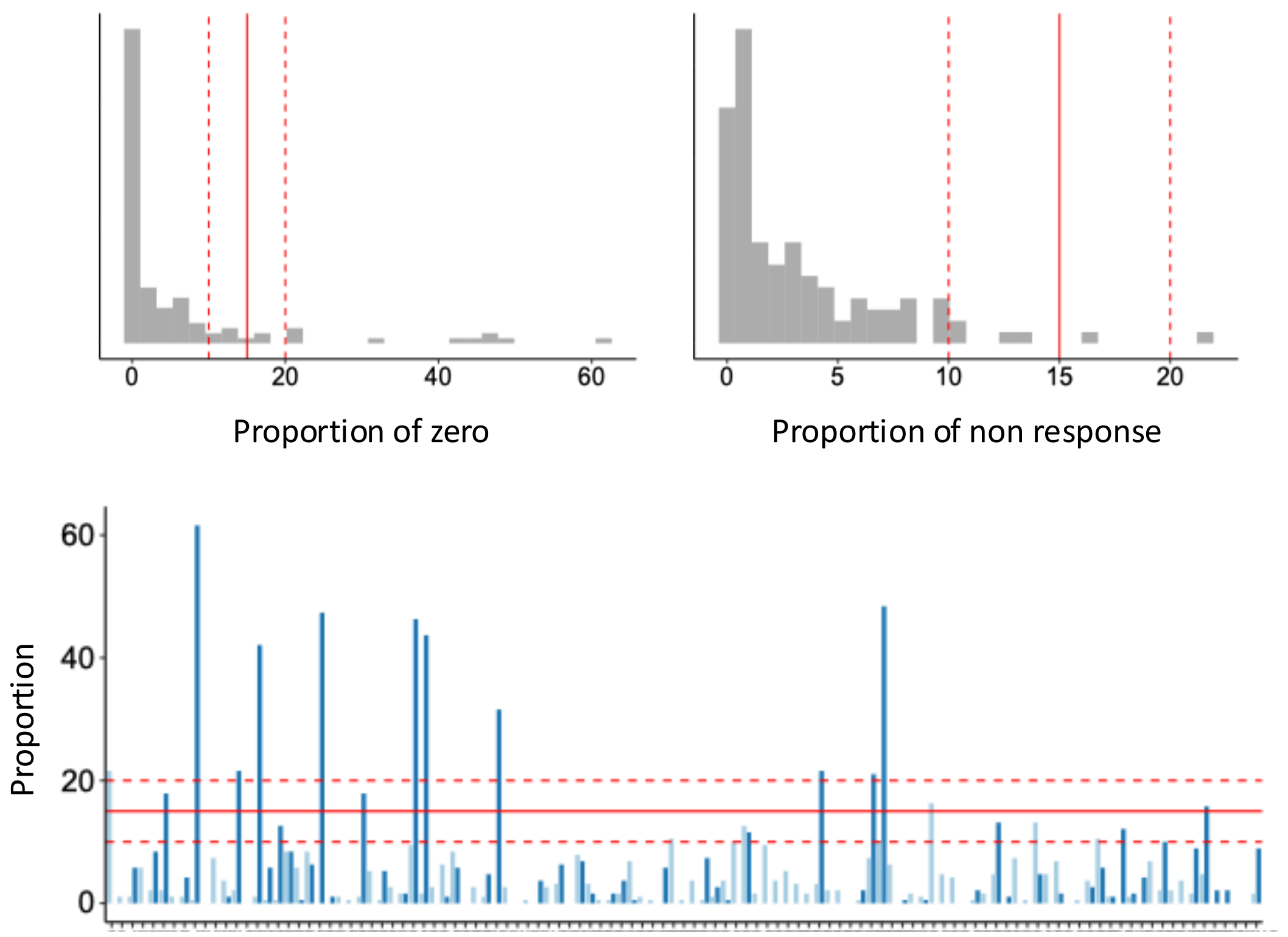

Supplementary Figure 8. Distribution of zero ratings and non-responses during the first (A) and second (B) RJT. Distribution of individual proportions of zero ratings (left) and non-responses (right) are provided in the top. Proportions values are provided for each individual in the bottom, with proportion of zero ratings in dark blue and of non-responses in light blue. The threshold we used to exclude participants (15%) is indicated with the solid red line. We also indicated alternative thresholds (10 and 20%) with the dashed red lines.

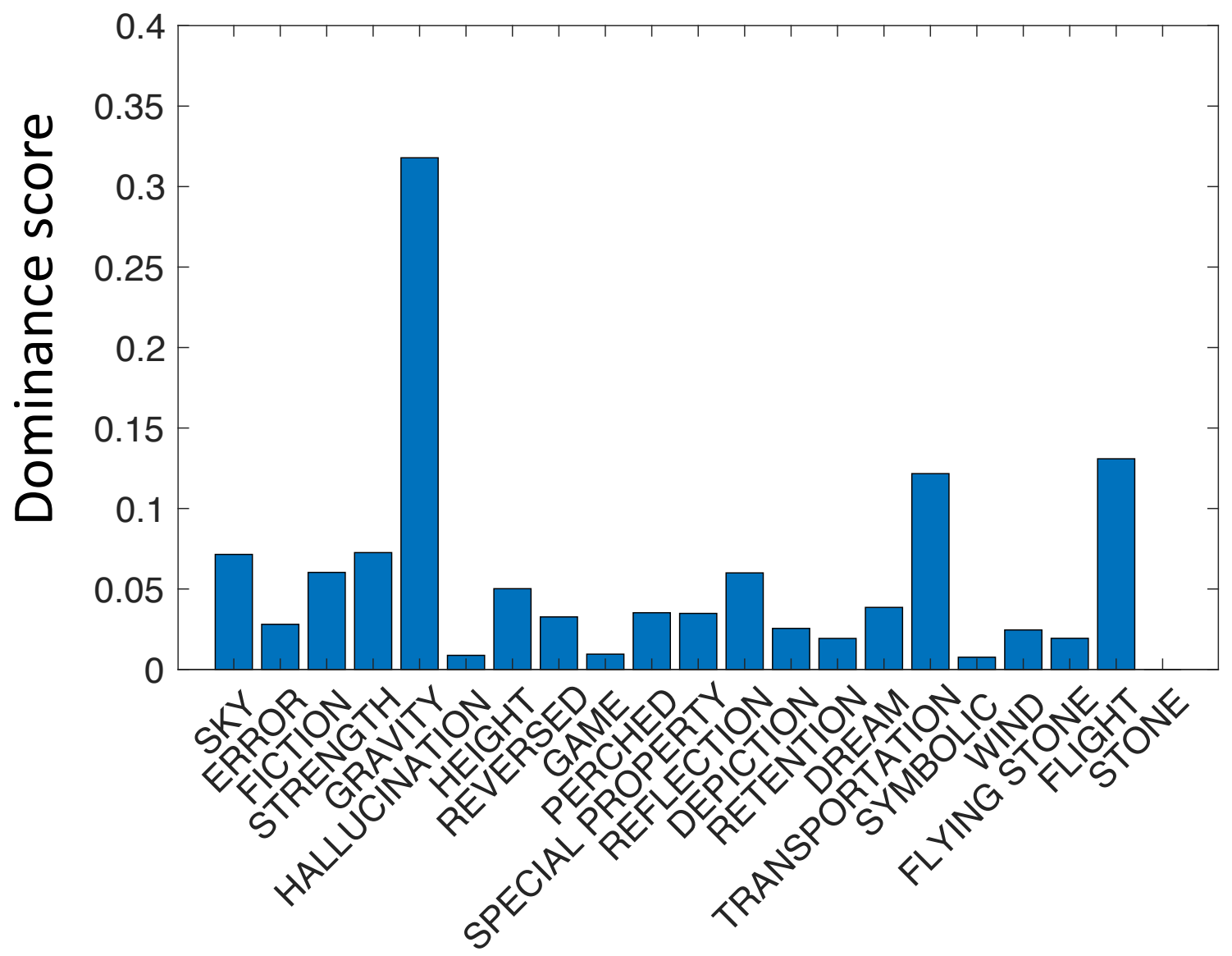

Supplementary Figure 9. Dominant representation of the problem.

For each of the 21 categories of responses, we computed the proportion of all the responses given by each participant that fell within this category. The *dominance score* corresponds to the averaged value of this latter proportion across participants (the higher the dominance score, the higher a category is repeated during the solving phase).

Supplementary Table

Word list	
French	English
**air**	**air**
arrivée	arrival
**atterrir**	**to land**
**avion**	**plane**
caillou	stone
chaussure	shoe
chiffre	digit
**ciel**	**sky**
cour	yard
dessin	drawing
écrire	to write
**espace**	**space**
**gravité**	**gravity**
jardin	garden
lancer	to throw
pied	foot
sauter	to jump
sol	ground
tracer	to trace
**voler**	**to fly**

Supplementary Table 1. Riddle-specific word list used in the RJT.
List of 20 words preselected for the riddle with their respective English translations. Word in bold were related to the dominant representation of the riddle.

Category	Definition
**Zoe literally throws a stone that lands in the sky**	
**Flight**	Zoe is flying (e.g., on a plane, an air balloon or a rocket)
Flying stone	The stone flies by itself
**Gravity**	Context of absence of gravity (e.g., the setting is in space or on the moon)
Height	Zoe throws the stone from an elevated place
Perched	The stone lands on something above the ground
Special property	The stone has a special property that enables it to land in the sky (e.g., very light)
Retention	Something keeps the stone in the sky (e.g., a magnet)
Strength	The stone is thrown with strength (by Zoe or with the help of a machine)
**Transportation**	A flying device takes the stone to the sky (e.g., a bird or plane wings)
Wind	The wind carries the stone to the sky
**Zoe throws a stone, but it does not literally land in the sky**	
Reversed	Earth and sky are reversed
Game	Context of a game (e.g., Zoe plays a game)
Reflection	Zoe throws the stone on something that reflects the sky (e.g., water, mirror…)
Sky	The sky refers to something other than the usual physical sky (e.g., a painting or a drawing)
**Zoe does not really throw a stone in the sky**	
Dream	Zoe is dreaming
Error	Zoe is mistaken or is lying
Fiction	Context of a fictional narrative fiction (e.g., a novel or a movie)
Hallucination	Zoe is hallucinating or on drugs
Depiction	Figural depiction of the scene (e.g., a drawing or a theatre play)
Stone	The stone is not a real stone (e.g., it is a bird)
Symbolic	Metaphoric phrase or symbolic meaning of the riddle

Supplementary Table 2. List of categories used to classify the responses.
Categories in bold indicate the most frequent categories of responses proposed by participants in [1]. Underlined categories indicate categories of responses close to the expected solution.

Supplementary references

1. Bieth, T. *et al.* Changes in semantic memory structure support successful problem-solving and analogical transfer. *Commun. Psychol.* **2**, 54 (2024).
2. Robinson, M. M., DeStefano, I. C., Vul, E. & Brady, T. F. Local but not global graph theoretic measures of semantic networks generalize across tasks. *Behav. Res. Methods* **56**, 5279–5308 (2024).
