# Supplementary material for: REM sleep favors the restructuring of problem-related semantic associations
Source: Commun Biol. 2026 Jun 3;9:1171. doi: 10.1038/s42003-026-10354-1 (PMC13542217; doi:10.1038/s42003-026-10354-1)
Supplement: Supplementary file 2 [file 42003_2026_10354_MOESM2_ESM.pdf]

nature portfolio

Corresponding author(s): Theophile Bieth (theo_bieth@hotmail.fr)

Last updated by author(s): 29/04/2026

Reporting Summary

Nature Portfolio wishes to improve the reproducibility of the work that we publish. This form provides structure for consistency and transparency in reporting. For further information on Nature Portfolio policies, see our Editorial Policies and the Editorial Policy Checklist.

Statistics

For all statistical analyses, confirm that the following items are present in the figure legend, table legend, main text, or Methods section.

n/a	Confirmed	
☐	☒	The exact sample size (*n*) for each experimental group/condition, given as a discrete number and unit of measurement
☐	☒	A statement on whether measurements were taken from distinct samples or whether the same sample was measured repeatedly
☐	☒	The statistical test(s) used AND whether they are one- or two-sided<br>*Only common tests should be described solely by name; describe more complex techniques in the Methods section.*
☐	☒	A description of all covariates tested
☐	☒	A description of any assumptions or corrections, such as tests of normality and adjustment for multiple comparisons
☐	☒	A full description of the statistical parameters including central tendency (e.g. means) or other basic estimates (e.g. regression coefficient) AND variation (e.g. standard deviation) or associated estimates of uncertainty (e.g. confidence intervals)
☐	☒	For null hypothesis testing, the test statistic (e.g. F, t, r) with confidence intervals, effect sizes, degrees of freedom and P value noted<br>*Give P values as exact values whenever suitable.*
☒	☐	For Bayesian analysis, information on the choice of priors and Markov chain Monte Carlo settings
☒	☐	For hierarchical and complex designs, identification of the appropriate level for tests and full reporting of outcomes
☐	☒	Estimates of effect sizes (e.g. Cohen's *d*, Pearson's *r*), indicating how they were calculated

Our web collection on statistics for biologists contains articles on many of the points above.

Software and code

Policy information about availability of computer code

Data collection	Behavioral data were collected using Psychopy v3.2.4 (open softwares: https://www.psychopy.org)
Data analysis	Analyses were conducted using open softwares and toolboxes available online as described in the Methods section. In particular, we used the Brain Connectivity Toolbox version 2019-03-03 running on Matlab R2020a to compute the semantic network metrics, and Rstudio v 1.4.1717 to run statistical analyses. EEG analysis were performed with the FOOOF and NICE toolbox.

For manuscripts utilizing custom algorithms or software that are central to the research but not yet described in published literature, software must be made available to editors and reviewers. We strongly encourage code deposition in a community repository (e.g. GitHub). See the Nature Portfolio guidelines for submitting code & software for further information.

Data

Policy information about availability of data

All manuscripts must include a data availability statement. This statement should provide the following information, where applicable:

- Accession codes, unique identifiers, or web links for publicly available datasets
- A description of any restrictions on data availability
- For clinical datasets or third party data, please ensure that the statement adheres to our policy

The study reported in this article was not formally preregistered. Experimental material is available in the Supplementary data associated with this article. The data

set generated and/or analyzed in the current study are available in a persistent repository. Data sharing is anonymzed and participants' demographical information are not included.

Research involving human participants, their data, or biological material

Policy information about studies with human participants or human data. See also policy information about sex, gender (identity/presentation), and sexual orientation and race, ethnicity and racism.

Reporting on sex and gender	We considered sex based on self-reporting but did not analysed or segregated sex data because we did not have an hypothesis on sex differences.
Reporting on race, ethnicity, or other socially relevant groupings	We did not report this demographical information.
Population characteristics	One hundred and eleven native French speakers aged between 18 and 34 years old (mean age = 21.6 years, SEM = 2.22 years, 77 women and 34 men) participated in this study.
Recruitment	Participants were recruited via the RISC platform (RISC-UAR 3332 CNRS, https://www.risc.cnrs.fr) for online announcement.
Ethics oversight	COMMITTEE FOR THE PROTECTION OF INDIVIDUALS (CPP) Ile-de-France III<br>n° : 2019-A00562-55<br>Ref. CPP : 3681–RM<br>Ref. CNRIPH : 19.03.05.55006

Note that full information on the approval of the study protocol must also be provided in the manuscript.

Field-specific reporting

Please select the one below that is the best fit for your research. If you are not sure, read the appropriate sections before making your selection.

☒ Life sciences ☐ Behavioural & social sciences ☐ Ecological, evolutionary & environmental sciences

For a reference copy of the document with all sections, see nature.com/documents/nr-reporting-summary-flat.pdf

Life sciences study design

All studies must disclose on these points even when the disclosure is negative.

Sample size	For this study, we predefined a target sample size of 120 participants, aiming for approximately 30 participants per group. This calculation factored in potential no-shows, cancellations, and the possibility that some individuals in the sleep groups might not fall asleep. It also consider the solving rate of our riddle based on previous published study.
Data exclusions	We excluded :<br>(i) participants with technical issues during the experiment.<br>(ii) participants who already knew the riddle or salved the riddle before the incubation period.<br>(iii) participants' RJT in which participants did not respond or rated the word pairs as zero in more than 15% of all trials in one RJT. We considered that these participants were not sufficiently engaged in the RJT and that missing or zero ratings would importantly bias the network with missing links.<br>(iv) participants who solved the riddle after the alloted time, during the RJT since we do not know if the solution came to his/her mind in the midst of performing the RJT.<br>(v) participants in the sleep group who slept.<br><br>All information related to data exclusions were detailled in dedicated figure. Exclusions criteria were pre established.
Replication	No replication was provided in the current study.
Randomization	We did not randomized the group attribution
Blinding	No blinding was done.

Reporting for specific materials, systems and methods

We require information from authors about some types of materials, experimental systems and methods used in many studies. Here, indicate whether each material, system or method listed is relevant to your study. If you are not sure if a list item applies to your research, read the appropriate section before selecting a response.

Materials & experimental systems

n/a	Involved in the study
☒	☐ Antibodies
☒	☐ Eukaryotic cell lines
☒	☐ Palaeontology and archaeology
☒	☐ Animals and other organisms
☒	☐ Clinical data
☒	☐ Dual use research of concern
☒	☐ Plants

Methods

n/a	Involved in the study
☒	☐ ChIP-seq
☒	☐ Flow cytometry
☒	☐ MRI-based neuroimaging

Plants

Seed stocks	NA
Novel plant genotypes	NA
Authentication	NA
