# Supplementary material for: REM sleep favors the restructuring of problem-related semantic associations
Source: Commun Biol. 2026 Jun 3;9:1171. doi: 10.1038/s42003-026-10354-1 (PMC13542217; doi:10.1038/s42003-026-10354-1)
Supplement: Supplementary file 3 [file 42003_2026_10354_MOESM3_ESM.pdf]

nature portfolio

Peer Review File

REM sleep favors the restructuring of problem-related semantic associations

Corresponding Author: Dr Théophile Bieth

This file contains all reviewer reports in order by version, followed by all author rebuttals in order by version.

Version 0:

Reviewer comments:

Reviewer #1

(Remarks to the Author)
Participants attempted to solve a riddle and completed a relatedness judgement task (RJT) for word pairs related to the riddle before and after an EEG-monitored incubation period that consisted of sleep (that included REM for a subset participants) or wakefulness. Memory restructuring across the incubation period was examined by creating SemNets based on the RJT before and after the incubation window and examining changes in various network properties. Results indicated that behaviorally, there was no difference in the ability to solve the riddle across participant groups. However, changes in multiple SemNet properties were greater for participants who achieved REM during incubation compared with those who remained awake, and these properties were associated with increases in multiple EEG indices that reflect cognitive richness. The authors conclude that these results indicate that memory restructuring occurs during REM, but additional processes are necessary for problem-solving success.

This is a well-constructed experiment and data analyses are very thorough and appropriate for the topic. The study overcomes a large weakness in the prior literature on the contribution of sleep to creative problem solving by operationalizing and empirically measuring memory restructuring. Although the SemNets used are necessarily quite simple, the fact that they captured changes in several network properties across the incubation period will be of interest to many and points to the usefulness of such measures in this line of research. I have a few concerns with the manuscript in its current form, as detailed below, but overall, this is a novel experiment that provides new insights about memory restructuring and REM sleep.

I am a bit concerned about the conclusion that the results suggest that additional processes beyond memory restructuring are necessary for successful problem solving (especially in the abstract). I agree that this is one possible reason that problem solving did not improve for the REM group relative to the wake group after incubation, but there are also several other explanations. Some of these are considered by the authors, but others are not. For example, although it appears that some restructuring occurred for participants who slept, it is possible that additional restructuring is necessary to generate the correct solution. Given that, on average, participants spent roughly 15 min in REM, more REM time may be necessary for more elaborate restructuring to take place.
Additionally, theoretically, a stable cortical problem representation must be created during non-REM stages prior to the restructuring that occurs during REM. Again, the nap may not have provided enough time for a stable problem representation to be created. I don't think any of these possible explanations are more likely than others, thus, this should be reflected in the manuscript.

The analysis examining whether participants in the REM group were "closer" to the correct solution than other participants seems superfluous, as this would suggest that an analytical, gradual path led participants to the solution. This is hard to reconcile with the notion that creative/insight problem solving is typically associated with a "eureka" moment and that in this experiment, participants who solved the riddle reported having a eureka feeling more often than non-solvers.

Multiple EEG features typically implicated in "cognitive richness" (i.e., states of consciousness, level of task engagement) were associated with changes in the SemNet properties across incubation, suggesting that cognitive richness predicts the amount of restructuring that takes place. Additional clarification on why these concepts may be related would be useful to aid in comprehension. Is the idea that more restructuring takes place when a person is closer to consciousness?

In the spectral power analyses of EEG data, is there a reason the sigma band was excluded (typically 12 – 15 Hz; here, these frequencies are included in the beta band, 12 – 30 Hz)? Given that sleep events are frequently isolated in the sigma band (e.g., sleep spindles), it seems odd that it was not considered in this experiment.

Reviewer #2

(Remarks to the Author)
In this study, the authors examined how REM sleep, compared to NREM sleep and wake, affects the ability a solve a verbal riddle as well as how it affects semantic/associative connections between words related to the riddle when analyzed through a semantic network approach (semNet). In addition, the authors examined how changes in the semNet metrics were related to global EEG measures collected during sleep. Their main findings were: (a) there was no significant effect of sleep on solving the riddle; (b) some changes in semNet metrics were more pronounced following sleep compared to wake, particularly for concepts deemed a-priori as semantically distant; and some of these were showing particular changes in REM sleep versus NREM sleep; (c) there were several correlations between global EEG measures during sleep and SemNet changes, scattered across REM and NREM.

Overall, this is an interesting study with partly inconsistent results. I have a few core concerns regarding the statistical analyses that must be thoroughly addressed; and then a series of other comments concerning issues like how the study is presented, its conclusions and so on.

Core concern:

1. My biggest concern with the analysis is the removal of 15 participants who had 15% of trials either unanswered or rated as 0 in the RJT task. This, to me, is not intuitive at all. It is one thing to remove an extreme participant who, say, answered 0 (or 100) to almost all the pairs; but 15% is a low and unsubstantiated threshold, and determining that participants were not sufficiently engaged because they thought, for example, that a fifth of the pairs should warrant a 0 rating does not seem reasonable to me. Looking at the authors' 2024 paper that informed the current study, a different threshold (10%) was used to remove participants. So, there is no consistency even with this seemingly arbitrary thresholding. The authors should present the full results including these 15 removed participants. Alternatively, they should present an unquestionable, evidence-based reason why these participants are clear outliers compared to the others. I do not object to the removal of very few participants here or there who are truly extreme in their response patterns (which, perhaps, may not allow for a faithful estimation of their SemNet metrics); but this vast removal looks dubious and unjustifiable.
2. Correction for multiple comparisons is not applied robustly or consistently, yielding some questionable results. The authors employed acceptable control for the multiple comparisons in the EEG analysis; but no similar control was employed for their previous analyses. Specifically, it is not clear why there is correction for 4 independent analyses of 4 different metrics when the EEG data were examined (48 in fact, which the 4 different metrics are part of), but there is no such control for the main results in Figures 2 and 3. Looking at the p-values, it seems to me that none of the main effect results in Figure 2A would survive such a correction, but the rest of the results would (especially the interaction results in Figure 2B, which are really the heart of the paper). If I'm correct, this would also be consistent with the additional bootstrapping analysis the authors present in the Supplementary (Figure S2). Please employ this correction, and only report results as significant if they pass the multiple comparison correction.
Other concerns:

Introduction:
1. There are repeated declarations that the general topic of the current study, the effects of (REM) sleep on restructuring of memories, has been scarcely studied. This is unjustifiable. Even the literature review in the Intro mentions quite a number of studies examining this very topic, and if we consider studies that looked at memory restructuring and sleep in general (not just REM sleep), there are many additional ones not cited that could fall under this definition (e.g., see my review paper Lerner & Gluck, 2019, which the authors cite). The authors emphasize in the Introduction that their study is unique because it decouples performance measures from measures of the mechanism (in this case, solving the riddle versus the SemNet analysis). I totally agree with THAT claim, and this is what needs to be emphasized in the introduction as the main motivation for the study, not the claim that the general topic of REM sleep and memory restructuring has not been examined much. Please remove phrases like "few studies have attempted…", "only two experimental studies", "is scarce" and so on.
2. "REM sleep supports neural plasticity and synapse reshaping" – this sentence and the whole paragraph surrounding it, need to be considered carefully. It is true that there are indications that REM sleep contributes to hyper-associativity, greater connectivity and bias towards excitation; however, there is also considerable evidence to the exact opposite, with REM sleep encouraging synaptic pruning and a bias towards inhibition. This known contradiction is, in fact, addressed and emphasized in the very first paragraph of one of the papers the authors cited (Aime et al., 2022). Please rewrite this paragraph to reflect the actual complexity regarding REM sleep as reflected in the literature.
3. "higher level of EEG activation" (last sentence of the introduction): Please shortly clarify, already at this point, what does "high" mean in this context.

Results:
1. Generally, the main weakness of the results is the lack of demonstrated association between performance on the riddle and either sleep or effects related to the SemNet metrics. The authors acknowledge it; however, they make little attempt to check these relations thoroughly on the pretense that too few REM subjects solved the riddle. But some things could still be examined across participant groups. For example, is there a relation between solvers (25 out of 111) on the first session and the baseline SemNet metrics? Is there a relation between solvers in the second session (10 out of 84) and differences in the

SemNet metrics across sessions? Even in the absence of a sleep effect, it should still be examined whether the SemNet metrics have relevance to the ability to solve the riddle. I realize that this very question was the focus of the authors' 2024 paper – but as far as I could see in that earlier paper, the relation was only shown directly for the Weight metric, whereas many of the strongest results in the current work were for the EV metric. I would expect that the authors could at least try to replicate the 2024 result, and maybe even uncover additional relations between SemNet metrics and solving abilities. And if there are none, this should be clearly noted and addressed in Discussion (see later).
2. Many of the results seem to be related to effects of sleep as a whole rather than REM sleep in particular. More can be done to examine the specific contribution of REM sleep. For example, including the percentage of REM sleep as a covariate in the model (or run a follow-up analysis where the contribution of REM sleep percentage is examined). This can be carried out either over all sleep participants, or just those achieving REM sleep, or both.
3. Please change the order of the results such that the analysis of performance on the riddle is presented first, and only then the SemNet results.

Discussion:
1. "…and our results were consistent with our previous study (Bieth, Kenett, et al., 2024). The only deviating result concerns the lack of significant effect for global SemNet changes for efficiency" – again, that's not proven. The authors should check relations between solvers and SemNet metrics across participant groups to see if they replicate the relation to Weight found in the 2024 study. They then need to discuss it – especially if it's not replicated – in the context of their other findings.
2. The authors mention N1 potential involvement in insight and cite Lacaus et al., 2021. Some results from my own lab using pink noise corroborated the potential importance of N1 in insight. Consider citing as well (Vickrey & Lerner, 2023). There might be more.

Methods:
1. It is mentioned that additional tasks, unrelated to the ones presented in this paper, were carried out before and after. What is the risk that those other tasks affected the current one? Are these other tasks consistent across participants? More information needs to be supplied (perhaps in the Supplementary) to alleviate concerns.
2. "Fischer test and corrected with Yates method" – I'm confused. Either you used a chi-square test - and then corrected with Yates if needed; or you used Fischer's exact test - and then no correction is needed at all. I'm not sure what "Fischer test" you refer to here.

Itamar Lerner, PhD

Reviewer #3

(Remarks to the Author)
This paper investigates how different sleep stages contribute to creative problem-solving by examining changes in semantic memory networks before and after a 90-minute incubation period that included either NREM sleep, REM sleep, or wakefulness. Using fine-grained EEG analyses, the authors show that REM sleep promotes the reorganization of problem-related associations, consistent with its proposed role as a "hyperassociative" state. Interestingly, this REM-related restructuring was not sufficient on its own to enhance problem-solving success. Overall, this is a very strong and well-designed study, which provides valuable insights into the links between sleep and problem-solving, applying a highly innovative approach. Nevertheless, I have the impression that the manuscript focuses somewhat too heavily on REM sleep, despite also uncovering intriguing findings regarding NREM sleep that deserve more attention and discussion.

I suggest revising the manuscript, taking into consideration the comments below, before proceeding to the next step.

Introduction:

"REM sleep dreams are typically longer (Antrobus, 1983; Casagrande et al., 1990; Foulkes, 1964), more vivid, hallucinatory (Hobson & Stickgold, 1994; Zadra et al., 1998), and metaphorical (Malinowski & Horton, 2021) than NREM sleep dreams." – Yes, this seems to be correct to some extent, nonetheless, some studies question this two-state model of dreaming and suggest that circadian factors might strongly influence the differences between NREM and REM dreams, indicating that nrem dreams become more similar to REM dreams in the morning hours. See for instance, Nielsen, T. A. (2004). Chronobiological features of dream production. Sleep Medicine Reviews, 8(5), 403-424.

„For example, a study found that REM sleep facilitates solving of anagram word puzzles (Walker et al., 2002)." – Although the cited study aimed to highlight the role of REM sleep in creative problem solving, the authors should note that int hat specific study REM only showed an advantage versus NREM sleep, but the advantage was equal to that of waking.

Methods:

1) Were all the participants familiar with the hopscotch game? I believe this might largely influence the semantic networks activated by the riddle. How could the authors take this into consideration?

2) „First, participants who found the solution after the second RJT (n = 3) were excluded" – I do not understand this. In Fig1/A the RJT is after the 2nd attempt of riddle solving and not before.

3) The pre-established word pairs are supposed to reflect the semantic networks activated by the task; however, isn't it likely that different individuals follow different associative paths to solve the riddle, and may arrive to the same solution differently?

4) Excluding participants with many 0 ratings seems a bit arbitrary to me, as these responses were also allowed, and reflect valid responses (of a lack of perceived associations) for the word pairs.

Results

Although the authors controlled for confounds such as TST to exclude the possibility that sleep duration and not specifically REM sleep was associated with the change in Semantic relatedness metric, based on the first table, the REM and NREM groups also differed in N2, SWS and WASO, however, these confounds were not taken into consideration.

The authors accentuate the specific effect of REM on semantic networks; however, in many outcome measures, REM and NREM sleep do not show significant differences. Do the authors consider that this is due to a lack of power? It is also interesting that the variability in the REM group seems to be much larger, suggesting that participants reaching REM considerably differ. Could the authors further explore why the variability in the REM group is much higher compared to the NREM and wake groups?

In Fig4/E - I recommend to highlight with different colours the participants from the REM and the NonREM only groups, to explore whether the NREMalpha-metric correlations are present in both groups or only in the REM group.

Discussion

"Of note, the absence of difference between the NREM and REM groups in some of our behavioural analyses and the NREM-related EEG results also suggests that the contribution of sleep in restructuring processes may be more complex, possibly involving the different NREM sleep stages that precede REM sleep." – This hypothesis could be tested by combining REM and NREM EEG measures as predictors of semantic network change.

Version 1:

Reviewer comments:

Reviewer #1

(Remarks to the Author)
I thank the reviewers for their quick and thorough responses to my comments. Nearly all my previous concerns have been sufficiently addressed, with the exception of the analysis of whether the REM group was "closer" to the correct solution than other participants. I agree that the analysis can still be reported, I just feel that there should be a brief explanation of how this idea of incremental steps leading one closer to the solution should be reconciled with the "eureka" feeling reported by solvers. It is unclear why participants were asked to report if they had a eureka feeling or not and what this tells us then. Is it that the subjective eureka experience comes suddenly, but only after a sufficient amount of incremental restructuring has taken place?

Also, as a side note, I agree with the author's response to Reviewer 2, where they note that research concerning the role of REM sleep in semantic restructuring is relatively sparse compared with the broader literature. However, the authors may be interested in the following study that was not cited but also addresses this issue:
Westerberg CE, Fickle SE, Troupe CE, Madden-Rusnak A, & Deason RG (2025). An afternoon nap facilitates analogical transfer in creative problem solving. Journal of Sleep Research, 34(4), e14419. https://doi.org/10.1111/jsr.14419

Reviewer #2

(Remarks to the Author)
I appreciate the authors' detailed answers to my comments. Their responses to most of them are satisfactory. However, regarding my two core concerns, I felt that their reply was mostly aimed at explaining away my worries rather than making significant changes to the manuscript, which I find insufficient. Before I go into the specifics, let me explain my general approach from a bird's eye-view: There are many ways to analyze data, which often yield many results – some stronger, some weaker. The way weaker results are handled depends on how meticulous the researchers want to be. Should everything be reported, even if the evidence is weak and the statistical control is not as rigorous as it can be? or should only the stronger results be reported, knowing that you are walking on firmer ground? Usually it's something in between, but my

view and experience is that there is a lot of noise that often gets mixed with true signal and doesn't reflect true effects, and the better the journal (and I consider Communications Biology a respectable platform) – the higher the emphasis should be on the stronger results, without attempting to milk findings to the last drop. The current study yielded many results, not always consistent with each other; but some results were clearly stronger than others, passing more rigorous statistical control – and those are the ones the manuscript should be focused on if accepted for publication.

Now for the details.

• Regarding removal of participants: I still find the 15% threshold to be quite arbitrary, and I do not agree with some of the authors' arguments. Let's treat things on a case-by-case basis. Regarding participants with high levels of non-responses, I am more inclined to accept the authors' justification that their data skews the network computation – missing data can do that – even though I have to admit that looking at the distribution, only the one with 36% seems like a true outlier. Regarding the participants with high proportions of zero rankings – while a value of 0 can in principle reflect all of the possibilities the researchers state rather than participants' true evaluation of the semantic distance, these are merely speculations. From my own personal experience with obtaining semantic relatedness ratings from subjects, if you ask them about it later – they would often stick by their extreme (e.g., zero) ratings. People's opinions vary widely on those things, often much more than what researchers seem to think are the "right" values. Either way, removing a big number of participants because the computational model suffers from their extreme value seems to be bad practice unless done very sparingly. The authors should apply an acceptable and strict threshold for removal of participants (maybe something like answers with >5 MAD from the median? I can't really tell without seeing the specific numbers, but it should be a something that can be supported by standard outlier removal methodology) that doesn't end up removing a high proportion of their participants). Looking at the histograms, the eye test suggests, as mentioned above, that only one participant is an outlier for the non-response participants, and maybe 2-4 for the zero proportion participants; but obviously the determination needs to be by the values rather than the eye. The authors should then report the results running on that exclusive number of participants in the main text; and only in the supplementary, if they insist, present results after applying a higher removal of participants.
o Additional sidenote: The authors mention (and I see in the histograms) 10 people removed due to zero proportions and 3 removed due to missing value. That sums up to 13. Where is the 14th removed participant coming from?

• Regarding correction of multiple comparisons: Again, while I appreciate the reviewer's argument that their behavioral hypotheses are a-priori and therefore should not be corrected for multiple comparisons, this is, at the bottom line, a decision of how statistically strict they want to be. It is not obvious to me that the 4 parameters mentioned are a subset of a bigger set of parameters, some of which are expected to yield effects, and some aren't (thus justifying concentrating on them without correction). In the Bieth et al (2024) paper, these are in fact the exact 4 parameters reported. The fact is that if the authors choose to be strict, their results would be more compatible with the alternative bootstrapping analysis in the supplementary. This, to me, is a good reason to focus on the strong result rather than report the weaker ones. The authors should definitely begin by reporting that strong result as their main finding (correcting for multiple comparisons). Only then, either in the supplementary or in a subsection of the main results, indicate that if they loosen their criterion and not correct for multiple comparisons, they also get these and these additional effects. They can then discuss the strength of the different results and their implications in the discussion (which they seem to have already began to). I would especially appreciate an interpretation of how they see their results in case only the strong finding is true and the rest is noise. Importantly, note that the final findings in this analysis will also probably be influenced to a degree by the inclusion of the omitted participants (my previous bullet point).

• Regarding previous literature: I appreciate the authors toning down their statements; still, they should be aware that there are more works showing links between REM sleep and creativity in the broader sense than they cite in the manuscript or mention in their response (e.g., Alger & Payne, 2016; Batterlink et al, 2014; the latter relates to semantic memory). If we are talking about links to sleep in general, or SWS, there are significantly more, including related to semantics (Tamminen et al, 2010 jumps to my mind; see my review, Lerner & Gluck, 2019 which the authors cite, for some more).

Reviewer #3

(Remarks to the Author)
The authors have adequately addressed my questions. I appreciate the additional analyses performed to study the combined role of NREM and REM sleep on performance.

Version 2:

Reviewer comments:

Reviewer #1

(Remarks to the Author)
The authors have adequately assuaged my concern regarding how the incremental steps that lead to a problem solution are reconciled with the "eureka" feeling that occurs when the solution is realized and have incorporated my suggested citation about the role of REM in semantic restructuring and I have no further suggestions.

Regarding Reviewer 2's comments 2 and 3, I think the authors have sufficiently addressed the concerns. They have controlled for multiple comparisons and moved any analyses that didn't survive the correction to the supplementary information. I also agree with the authors that they do not need to include further citations about REM sleep and creativity "in

the broader sense”. The articles suggested by the reviewer were focused on memory, not creative problem solving, and the manuscript already includes the citation for a review article that summarizes many of those studies.

For comment 1 from Reviewer 2, I commend the authors for their extensive consideration of how best to exclude outliers. They completed an analysis using MAD to identify outliers as suggested, which ended up excluding even more participants than their original method. They have provided an extensive explanation for the decision to stick with the 15% cut-off and are transparent by providing the data in supplementary figure 8. I think that is the best that can be done here. However, there is one part that is still confusing. In the “side note” the authors included in their response to this comment, it says that 19 participants were excluded for reasons related to the RJT. In the manuscript on p. 8, it says 21 were excluded for “various task-specific reasons”. However, in the method section on p. 20, it says 19 were excluded. Is the 21 on p. 8 a typo? If not, why were the other 2 participants excluded?

Open Access This Peer Review File is licensed under a Creative Commons Attribution 4.0 International License, which permits use, sharing, adaptation, distribution and reproduction in any medium or format, as long as you give appropriate credit to the original author(s) and the source, provide a link to the Creative Commons license, and indicate if changes were made.
In cases where reviewers are anonymous, credit should be given to 'Anonymous Referee' and the source.
The images or other third party material in this Peer Review File are included in the article’s Creative Commons license, unless indicated otherwise in a credit line to the material. If material is not included in the article’s Creative Commons license and your intended use is not permitted by statutory regulation or exceeds the permitted use, you will need to obtain permission directly from the copyright holder.
To view a copy of this license, visit https://creativecommons.org/licenses/by/4.0/

Reviewer #1

R#1 Global comments

Participants attempted to solve a riddle and completed a relatedness judgement task (RJT) for word pairs related to the riddle before and after an EEG-monitored incubation period that consisted of sleep (that included REM for a subset participants) or wakefulness. Memory restructuring across the incubation period was examined by creating SemNets based on the RJT before and after the incubation window and examining changes in various network properties. Results indicated that behaviorally, there was no difference in the ability to solve the riddle across participant groups. However, changes in multiple SemNet properties were greater for participants who achieved REM during incubation compared with those who remained awake, and these properties were associated with increases in multiple EEG indices that reflect cognitive richness. The authors conclude that these results indicate that memory restructuring occurs during REM, but additional processes are necessary for problem-solving success.

This is a well-constructed experiment and data analyses are very thorough and appropriate for the topic. The study overcomes a large weakness in the prior literature on the contribution of sleep to creative problem solving by operationalizing and empirically measuring memory restructuring. Although the SemNets used are necessarily quite simple, the fact that they captured changes in several network properties across the incubation period will be of interest to many and points to the usefulness of such measures in this line of research. I have a few concerns with the manuscript in its current form, as detailed below, but overall, this is a novel experiment that provides new insights about memory restructuring and REM sleep.

We thank the Reviewer for their overall positive assessment of our study, constructive feedback, and help improving our manuscript. We address all the comments in detail below. Each of the Reviewer's comments are written below in italic, our response is in plain text, and the citations from the revised manuscript are highlighted in blue. We also made minor changes to further improve the manuscript, all highlighted in yellow.

R#1 Comment #1

I am a bit concerned about the conclusion that the results suggest that additional processes beyond memory restructuring are necessary for successful problem solving (especially in the abstract). I agree that this is one possible reason that problem solving did not improve for the REM group relative to the wake group after incubation, but there are also several other explanations. Some of these are considered by the authors, but others are not. For example, although it appears that some restructuring occurred for participants who slept, it is possible that additional restructuring is necessary to generate the correct solution. Given that, on average, participants spent roughly 15 min in REM, more REM time may be necessary for more elaborate restructuring to take place.

Additionally, theoretically, a stable cortical problem representation must be created during non-REM stages prior to the restructuring that occurs during REM. Again, the nap may not have provided enough time for a stable problem representation to be created. I don't think any of these possible explanations are more likely than others, thus, this should be reflected in the manuscript.

We agree with the Reviewer that alternative explanations are possible. To account for this possibility, we extend the Discussion as follows:

p. 1: “While REM drove memory restructuring, it alone did not boost problem-solving success, suggesting that more extensive restructuring or additional processes are necessary for creative breakthroughs.”

p. 17: “Fourth, the REM sleep-related restructuring effect may not be sufficient to induce successful problem-solving on its own. For instance, additional restructuring, potentially during longer REM sleep periods, might be necessary to reach the optimal problem representation required for solving success. This interpretation is consistent with insight theories (Bowden et al., 2005; Siegler, 2000), proposing that restructuring needs to exceed a threshold in order to be consciously integrated and lead to an insight solving.”

p. 17: “Finally, a more integrative hypothesis, supported by theoretical models (Deperrois et al., 2022; Giuditta, 2014; Landmann et al., 2014; Lewis et al., 2018; Shuster et al., 2025), is that NREM and REM stages work in tandem across the sleep cycle: NREM supports the initial stabilization and offline consolidation of memory traces, which then serve as the substrate for REM-dependent associative restructuring. In our study, participants had a 90-minute nap, a duration that might have restricted both the depth of NREM-mediated consolidation and the subsequent opportunity for REM sleep to act upon a sufficiently stabilized memory trace. Future studies using longer sleep periods or multiple sleep cycles could help disentangle the distinct and interactive contributions of NREM and REM stages to creative problem-solving.”

R#1 Comment #2

The analysis examining whether participants in the REM group were “closer” to the correct solution than other participants seems superfluous, as this would suggest that an analytical, gradual path led participants to the solution. This is hard to reconcile with the notion that creative/insight problem solving is typically associated with a “eureka” moment and that in this experiment, participants who solved the riddle reported having a eureka feeling more often than non-solvers.

We thank the Reviewer for this interesting comment. We believe that both mechanisms – insight and non-insight - are possible for solving problems. To our opinion, it is not because most solvers solved the problem with a Eureka that a Eureka is mandatory to solve the problem. Durso et al’s study investigated SemNet changes during the problem-solving phase, repeating the computation of individual networks longitudinally over time (Durso et al., 1994). Although they considered that participants solved the problem with insight based on the nature of the problem (and not the Eureka report), they showed that SemNet restructuring occurred progressively during the solving phase in solvers compared to non-solvers. This gradual restructuring was also observed in other studies using different insight problems (Cushen & Wiley, 2012; Danek et al., 2020). In our previous study (Bieth et al., 2024), we showed that SemNet metric changes were significatively higher in participants who solved the problem with a Eureka compared to other solvers. It echoes the previous comment suggesting that solving the riddle may require reaching a restructuring threshold that could be higher (or more quickly reached) for insight solving. These previous findings

motivated us to keep the analysis examining whether participants in the REM group were "closer" to the correct solution than other participants.

R#1 Comment #3

Multiple EEG features typically implicated in "cognitive richness" (i.e., states of consciousness, level of task engagement) were associated with changes in the SemNet properties across incubation, suggesting that cognitive richness predicts the amount of restructuring that takes place. Additional clarification on why these concepts may be related would be useful to aid in comprehension. Is the idea that more restructuring takes place when a person is closer to consciousness?

We apologize for the lack of clarity of our EEG analyses and interpretation. In brief, increasing evidence in the field of consciousness suggests that the complexity and nature of a neurophysiological state constrain the complexity of the cognitive processes it can support. For example, EEG measures, such as faster frequency activity or increased complexity, have been associated with better prognosis in disorders of consciousness, higher level of engagement in a cognitive task during wake, or responsiveness to auditory stimuli during sleep. We reckoned that memory restructuring, a process that requires the breaking of obvious associations and the formation of novel ones in memory, similarly might necessitate a neurophysiological mode that is rich, complex, with a wide repertoire of possible neural configuration.

We clarified the hypotheses that led us to empirically test it in the current study, as follows:

> p.3: "These observations suggest that the complexity and nature of a neurophysiological state constrain the complexity of the cognitive processes it can support and might thus be particularly relevant for memory restructuring, - a 'complex' process requiring the formation of novel associations in memory. Although satisfying, this hypothesis remains to be tested."

> p. 16: "In addition, to demonstrate that restructuring occurs during sleep, we extend these previous accounts to a more complex cognitive process, showing that the quality of brain activity during REM sleep (measured by EEG markers) is a key determinant of the extent of restructuring. This provides an interesting mechanistic link between the neurophysiological properties of the sleeping brain and the dynamic of offline semantic memory organization."

We also clarified the expected results of our EEG analyses, as follows:

> p.5: "Finally, in an exploratory analysis, we examined whether REM sleep-related memory restructuring and/or creative benefit would be reflected by specific EEG markers -such as faster cortical activity, higher complexity, or flatter spectral slope-, all thought to reflect a neurophysiological mode that is rich, with a wide repertoire of possible neural configurations, and allowing for complex cognitive processes to take place. We hypothesized that the cognitive complexity of memory restructuring should likewise be reflected in a richer cortical state."

R#1 Comment #4

In the spectral power analyses of EEG data, is there a reason the sigma band was excluded (typically 12 – 15 Hz; here, these frequencies are included in the beta band, 12 – 30 Hz)? Given that sleep events are frequently isolated in the sigma band (e.g., sleep spindles), it seems odd that it was not considered in this experiment.

We agree with the Reviewer that sleep studies typically do not combine the sigma (spindle frequency) and beta bands. We chose these spectral frequencies because they have been robustly associated with changes in consciousness states in the consciousness literature (see for example Sitt et al., 2014, who compared the efficiency of various measures in differentiating patients with different disorders of consciousness). These same measures have also proven effective in differentiating periods of reactiveness versus non-reactiveness to external sounds within a same global state (a sleep stage) in a recent study from our group (Türker et al., 2023). For a better comparability with this body of work, we chose to keep the same EEG markers.

However, to address the Reviewer's concern, we performed an additional analysis in which we extracted the sigma frequency band separately from the beta one (i.e., sigma 12-15 Hz, and beta 15-30 Hz). The results were largely similar (see figure below), except for two points. First, we lost the significant positive association between remoteness-based SemNet changes and power in theta frequency band during REM for *weight*, possibly due to the increased number of statistical comparisons which were corrected for. Second, the negative association between remoteness-based SemNet changes and power in beta frequency band during REM became statistically significant after removing sigma frequencies from our initial beta band.

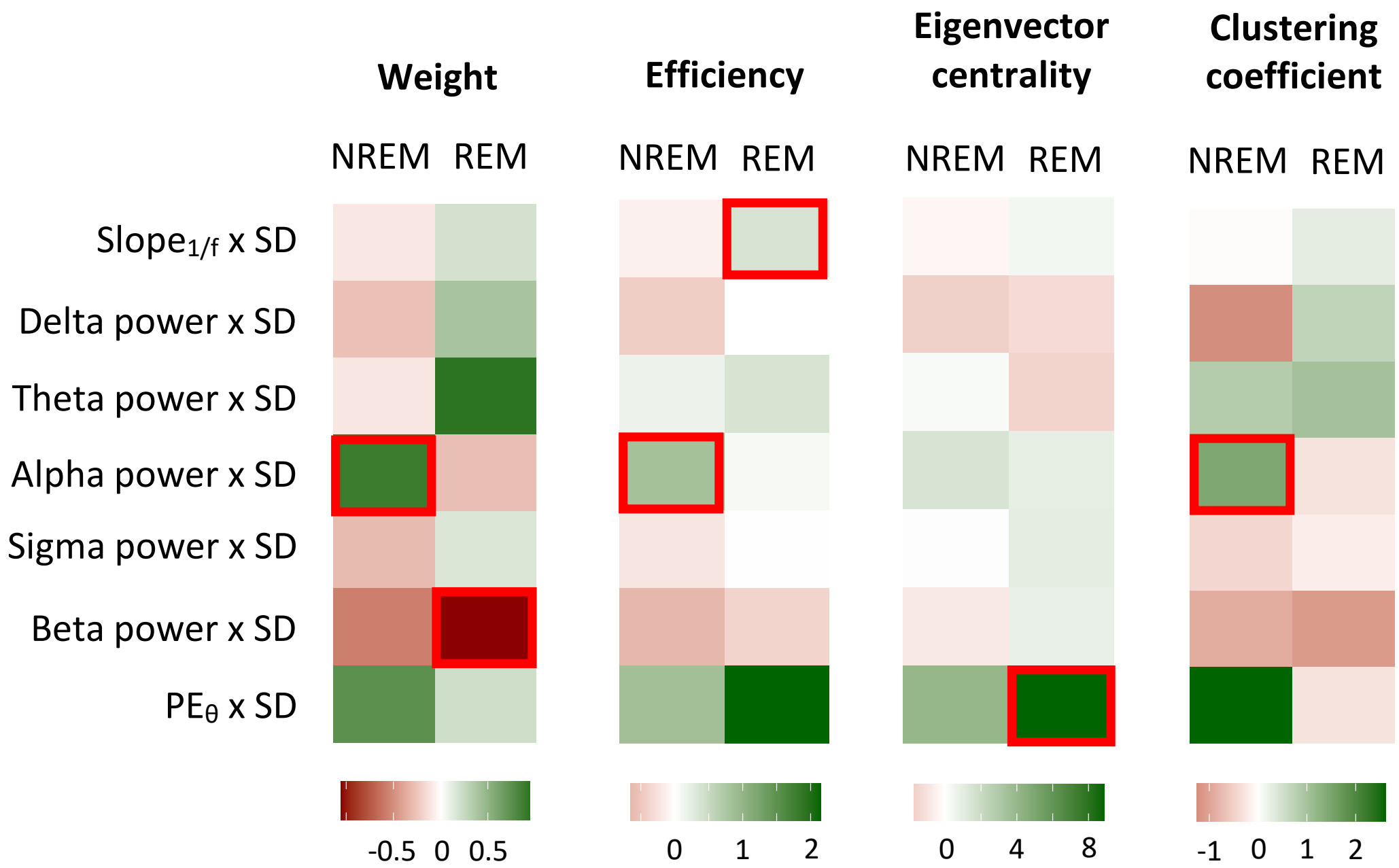

We chose to keep our initial analysis as it was hypothesis-driven based on the neuroscience of consciousness literature and on previous works of our team. However, we made our EEG data with sigma frequency band available on the public persistent repository associated with our article (see Data availability section) to allow comparisons with sleep studies. In addition, if the Editor or Reviewer strongly feel that we should include this analysis in Supplementary Information, we could do so.

Reviewer #2

R#2 Global comments

In this study, the authors examined how REM sleep, compared to NREM sleep and wake, affects the ability a solve a verbal riddle as well as how it affects semantic/associative connections between words related to the riddle when analyzed through a semantic network approach (semNet). In addition, the authors examined how changes in the semNet metrics were related to global EEG measures collected during sleep. Their main findings were: (a) there was no significant effect of sleep on solving the riddle; (b) some changes in semNet metrics were more pronounced following sleep compared to wake, particularly for concepts deemed a-priori as semantically distant; and some of these were showing particular changes in REM sleep versus NREM sleep; (c) there were several correlations between global EEG measures during sleep and SemNet changes, scattered across REM and NREM.
Overall, this is an interesting study with partly inconsistent results. I have a few core concerns regarding the statistical analyses that must be thoroughly addressed; and then a series of other comments concerning issues like how the study is presented, its conclusions and so on.

We thank the Reviewer for their overall positive assessment of our study, constructive feedback, and help improving our manuscript. We address all the comments in detail below. Each of the Reviewer's comments are written below in italic, our response is in plain text, and the citations from the revised manuscript are highlighted in blue. We also made minor changes to further improve the manuscript, all highlighted in yellow.

R#2 Comment #1

My biggest concern with the analysis is the removal of 15 participants who had 15% of trials either unanswered or rated as 0 in the RJT task. This, to me, is not intuitive at all. It is one thing to remove an extreme participant who, say, answered 0 (or 100) to almost all the pairs; but 15% is a low and unsubstantiated threshold, and determining that participants were not sufficiently engaged because they thought, for example, that a fifth of the pairs should warrant a 0 rating does not seem reasonable to me. Looking at the authors' 2024 paper that informed the current study, a different threshold (10%) was used to remove participants. So, there is no consistency even with this seemingly arbitrary thresholding. The authors should present the full results including these 15 removed participants. Alternatively, they should present an unquestionable, evidence-based reason why these participants are clear outliers compared to the others. I do not object to the removal of very few participants here or there who are truly extreme in their response patterns (which, perhaps, may not allow for a faithful estimation of their SemNet metrics); but this vast removal looks dubious and unjustifiable.

We understand the Reviewer's concern that excluding participants who had 15% of trials unanswered and/or rated as zero during the RJT may seem excessive and arbitrary. However, we believe these exclusions are justified for three key reasons: (1) the distribution of responses indicates that most excluded participants exhibited response patterns that substantially exceeded reasonable variability; (2) a high proportion of zeros and non-responses considerably affects the SemNet metric computation and would introduce a bias that is particularly problematic for longitudinal analyses; and (3) zero responses are inherently ambiguous and may reflect disengagement rather than true absence of associations. Below, we elaborate on each of these points, show how the 15% threshold is relevant to exclude

participants with extreme values of zeros or non-responses, and present supplementary analyses including all participants to demonstrate the robustness of our findings.

In the behavioral and EEG analyses that required SemNet metrics, we excluded participants who had a cumulated proportion of zeros and/or non-responses (over the 190 ratings they had to do during the first and/or the second RJT) higher than 15% (n = 14, 12.6% of all participants). We acknowledge that the threshold we used can appear somewhat arbitrary. We chose this threshold based on the general distribution of the zeros/non-responses data in our participants. Please see below the distribution of proportion of zeros and non-responses for each participant during the first and second RJT, with the 15% threshold indicated by a solid, red bar. These graphs show that excluded participants largely exceeded this 15% threshold. In the plots, we also indicated alternative thresholds (i.e., 10% and 20%, in dashed lines). Among the 14 excluded participants, three had a high proportion of non-responses (22%, 36%, and 16%), which represents data loss that affects network computation. The other participants (n = 10) had a high proportion of zeros (mean = 34%, SD = 16%, minimum 17%, maximum 62%, > 20% for 9 of them). We observed that the threshold choice did not drastically change the count of excluded participants.

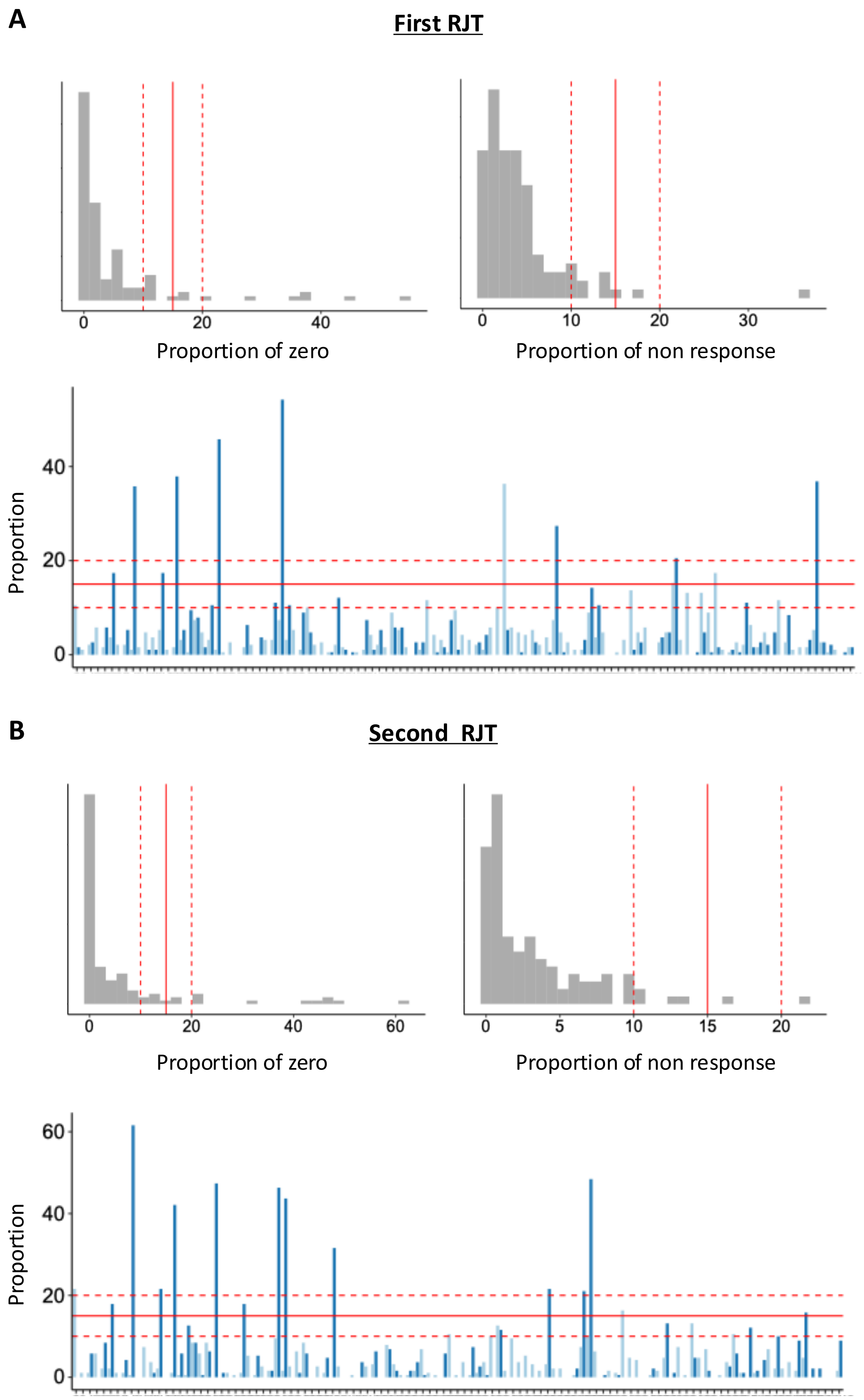

Figure. Distribution of zero ratings and non-responses during the first (A) and second (B) RJT. For each RJT is displayed 1) the distribution of individual proportions of zero ratings (left) and non-responses (right) and 2) below the proportion values for each individual, with proportion of zero ratings in dark blue and of non-responses in light blue. Threshold we used to exclude participants (15%) is indicated with the solid red line. We also indicated alternative thresholds (10 and 20%) with the dashed red lines.

To reassure the Reviewer on the robustness of our findings, we ran our main behavioral analyses with all participants who did not solve the riddle pre-incubation, including the 14 participants we excluded in our original manuscript. Hence, this new sample is composed of 79 participants (18 in the Wake group, 20 in the NREM group, and 41 in the REM group).

Crucially, our main results showing that REM sleep was associated with remoteness-based SemNet changes still hold in the full sample. Regarding local changes, the interaction effect of *semantic distance* and Group on SemNet metric changes remain significant for *weight* ($F(2,13836) = 8.57$, $p = 1.91 \times 10^{-4}$, $\eta^2 = .001$), *efficiency* ($F(2,14928) = 7.66$, $p = 4.75 \times 10^{-4}$, $\eta^2 = .001$) and *eigenvector centrality* ($F(2,1498) = 8.21$, $p = 2.85 \times 10^{-4}$, $\eta^2 = .01$). Post-hoc analyses revealed that the slope (representing the relationship between *semantic distance* and △Metric) was significantly steeper in the REM group compared to the Wake group (*weight*: $F(1,10319) = 15.13$, $p = 1.01 \times 10^{-4}$, $\eta^2 = .002$; *efficiency*: $F(1,11149) = 13.10$, $p = 2.97 \times 10^{-4}$, $\eta^2 = .001$; *eigenvector centrality*: $F(1,1119) = 15.92$, $p = 7.05 \times 10^{-5}$, $\eta^2 = .01$). In addition, post hoc tests showed significant differences between the REM and NREM groups for *efficiency* ($F(1,11527) = 6.25$, $p = .012$, $\eta^2 = .001$), but not for *weight* ($F(1,10628) = .01$, $p = .94$) nor for *eigenvector centrality* ($F(1,1157) = 2.72$, $p = .10$). Finally, the NREM group had a steeper slope compared to the Wake group for *weight* ($F(1,6725) = 12.78$, $p = 3.52 \times 10^{-4}$, $\eta^2 = .002$), but not for *eigenvector centrality* ($F(1,720) = 4.84$, $p = .028$ – non-significant after correction for multiple comparison) nor for *efficiency* ($F(1,7180) = .1.11$, $p = .29$). These results indicate that REM sleep enhanced local SemNet changes by increasing the centrality (*eigenvector centrality* increased) and the connection (*weight* and *efficiency* increased) of nodes and edges that were semantically remote. Of note, similarly to our original analysis, the *semantic distance* x Group interaction effect was not statistically significant for *clustering coefficient* ($F(2,1498) = .82$, $p = .44$).

The results on the effect of REM sleep on global SemNet changes seem less robust when taking the full sample than in the original analysis. When computing the main effect of Group on SemNet metric changes, was significant for *clustering coefficient* ($F(2,76) = 3.26$, $p = .044$, $\eta^2 = .08$). Although the results go in the same direction as our initial analyses, post-hoc tests did not allow to draw conclusions, since no results remained significant after correction for multiple comparisons (REM vs NREM: $F(1,59) = .32$, $p = .57$; REM vs Wake: $F(1,57) = 6.07$, $p = .0168$, $\eta^2 = .10$; Wake vs NREM: $F(1,36) = 4.27$, $p = .046$, $\eta^2 = .10$). The main effect of Group was not significant for the other metrics (*weight*: $F(2,73) = 2.45$, $p = .09$; *efficiency*: $F(2,76) = .59$, $p = .56$; *eigenvector centrality*: $F(2,76) = 2.07$, $p = .13$).

In sum, our results remained very similar when including vs excluding participants who had > 15% of zeros/non-responses during the RJTs. The only deviating results were: (i) the loss of some results concerning the sleep-related global restructuring, and (ii) the loss of significant differences between REM vs NREM, and NREM vs Wake, in the post-hoc tests for *eigenvector centrality* (however, our key result, - the difference when comparing REM and Wake groups- remains significant).

If the Editor or Reviewer strongly feel that we should include this analysis in Supplementary Information, we could do so. However, we still believe that our initial analysis is 'cleaner' because the meaning behind a zero rating and a non-response is clearly ambiguous.

As the Reviewer proposed, zero ratings can reflect true semantic unrelatedness, meaning that the participant genuinely perceives the two words as completely unrelated, which would provide valid information about their mental semantic memory structure. However, zero ratings could be interpreted in several other ways: (i) they could indicate non-engagement or low effort, in which the participant selects zero indiscriminately or without carefully considering the word pair, perhaps due to fatigue, boredom, or misunderstanding of the task instructions. Of note, we specifically asked participants in the instruction before the RJT completion to use the entire visual scale for rating their relatedness judgment, and the 20 words were beforehand shown to the participant; (ii) they may be used as a substitute for "I don't know" or missing knowledge, when the participant cannot evaluate the pair as the task does not provide a specific dedicated option; (iii) they might reflect a systematic response bias, where the participant habitually overuses extreme low values, either as a personal response style or because of fatigue (e.g., skewing the participant's worldview as "black or white").

This is particularly relevant because zero ratings affect the SemNet metrics more than any other value, as over-inflated zero ratings (i) reduce the density of the network, making it appear sparser than it truly is; (ii) lower the *clustering coefficient* by eliminating triangles of connections, giving the impression of weaker local coherence; (iii) increase path lengths and reduce global *efficiency*, making the network appear less integrated; and (iv) can artificially inflate modularity, suggesting more compartmentalized substructures, and destabilize *eigenvector centrality*, making key nodes appear less important or central than they are.

In a longitudinal design with two timepoint as ours, making sure that we exclude "bad" ratings is particularly important because any zero-inflated or low-quality responses at either time point can bias the observed within-subject differences, potentially leading to misleading conclusions about changes in semantic structures over the resting period. Therefore, carefully assessing zero ratings and non-responses is critical to ensure that observed changes over time reflect genuine cognitive differences rather than data noise.

To account for the Reviewer's comment, we clarified in the method section the threshold we used to exclude participants based on the RJT completion as follows :

> p.22: "Finally, we excluded participants based on their RJT completion quality. Individuals with more than 15% of non-responses (i.e., no response within the allotted time) and/or ratings of zero in the first and/or second RJT were excluded (n = 14). This exclusion was motivated by three critical concerns: (1) zero ratings and non-responses are inherently ambiguous and may reflect disengagement rather than true absence of associations, (2) any zero-inflated or low-quality responses at either time point can systematically distort the detection of true within-subject changes in semantic structure, and (3) zero ratings affect the SemNet metrics more than any other value (see *Supplementary Note 7*). Although arbitrary, this threshold effectively identifies participants whose response patterns deviate substantially from the sample distribution (see data distribution on **Figure S6**) and whose inclusion would risk compromising network stability and interpretability. The final sample used for SemNet analyses included 65 participants (wake: n = 17; NREM: n = 17; REM: n = 31)."

We added the *Supplementary Note 7*, and the **Figure S6** to provide more precise justification for participants' exclusion.

By reviewing the manuscript, we identified an error in the count of excluded participants. There were two participants excluded for technical issue and 14 based on the RJT completion. It did not impact the analyses as the excluded participants remained the same. We have corrected this error in the manuscript and **Figure S1**:

> p.22: "Second, two additional participants were excluded due to technical issues during the RJT."

R#2 Comment #2

Correction for multiple comparisons is not applied robustly or consistently, yielding some questionable results. The authors employed acceptable control for the multiple comparisons in the EEG analysis; but no similar control was employed for their previous analyses. Specifically, it is not clear why there is correction for 4 independent analyses of 4 different metrics when the EEG data were examined (48 in fact, which the 4 different metrics are part of), but there is no such control for the main results in Figures 2 and 3. Looking at the p-values, it seems to me that none of the main effect results in Figure 2A would survive such a correction, but the rest of the results would (especially the interaction results in Figure 2B, which are really the heart of the paper). If I'm correct, this would also be consistent with the additional bootstrapping analysis the authors present in the Supplementary (Figure S2). Please employ this correction, and only report results as significant if they pass the multiple comparison correction.

Thanks for the comment. Our behavioral analyses were hypothesis-driven, focusing on only a subset of four, specific SemNet metrics (*weight*, *efficiency*, *eigenvector centrality*, and *clustering coefficient*) that our previous work (Bieth et al., 2024) identified as sensitive to capture SemNet changes associated with successful problem-solving. Given these strong a priori hypotheses, we did not apply corrections for multiple comparisons across the four measures. However, we applied a rigorous correction for multiple comparisons for post-hoc tests that lead to the interpretations and conclusions we proposed in the manuscript. As mentioned by the Reviewer, we provided additional bootstrapping analyses. These analyses do not control for multiple comparisons (there are no permutations) and were done to investigate whether the unbalanced sample sizes could have impacted our results (see *Supplementary Note 4*). Since the EEG analyses were more exploratory than the behavioral analyses, we applied corrections for multiple comparisons.

We understand the Reviewer's concerns about the main effects in Figure 2A (global restructuring) potentially not surviving a correction for multiple comparisons. Nevertheless, the interaction effects in Figure 2B, which represent the core theoretical contribution of our paper, remain robust regardless of correction methods. To address the Reviewer's concern while maintaining our hypothesis-driven focus, we have revised our manuscript to emphasize the interaction effects as the primary findings, while toning down our conclusions related to global restructuring, as follows:

> p.9: “These results suggest that individual SemNets became more interconnected (*clustering coefficient* increased) and more strongly connected (*weight* increased) with more central nodes (*eigenvector centrality* increased) after the incubation period for participants who reached REM sleep compared to those who did not sleep.”

> p.11: “This sleep-related global restructuring must be interpreted with caution.”

> p.12: “Overall, REM sleep was mainly associated with local remoteness-based SemNet changes.”

> p.15: “Confirming our hypotheses, we found that REM sleep yields a restructuring of semantic associations mainly targeting the semantically remote associations of ideas in problem-related concepts.”

We also add in the method section explanation on why we did not correct our behavioral results for multiple comparisons:

> p.26: “As our analyses were hypothesis-driven (we focused on specific SemNet metrics identified in our previous study), we did not apply corrections for multiple comparisons across the four measures.”

R#2 Comment #3

There are repeated declarations that the general topic of the current study, the effects of (REM) sleep on restructuring of memories, has been scarcely studied. This is unjustifiable. Even the literature review in the Intro mentions quite a number of studies examining this very topic, and if we consider studies that looked at memory restructuring and sleep in general (not just REM sleep), there are many additional ones not cited that could fall under this definition (e.g., see my review paper Lerner & Gluck, 2019, which the authors cite). The authors emphasize in the Introduction that their study is unique because it decouples performance measures from measures of the mechanism (in this case, solving the riddle versus the SemNet analysis). I totally agree with THAT claim, and this is what needs to be emphasized in the introduction as the main motivation for the study, not the claim that the general topic of REM sleep and memory restructuring has not been examined much. Please remove phrases like “few studies have attempted…”, “only two experimental studies”, “is scarce” and so on.

We appreciate the Reviewer's perspective regarding the existing literature. While we acknowledge that some studies have touched on related topics, we maintain that the specific investigation of REM sleep's role in semantic memory restructuring remains relatively understudied compared to the broader sleep-memory consolidation literature, despite being widely mentioned in theoretical papers. Many of the studies the Reviewer references (including those in Lerner & Gluck, 2019) focus on different forms of memory processing (e.g., procedural sequence learning, temporal regularity extraction) rather than the creative semantic restructuring that is central to our work. The studies specifically examining REM sleep and creativity with semantic material (e.g., Cai et al., Wagner et al. Stickgold et al.) remain limited in number and were conducted over decade(s) ago.

That said, we appreciate the Reviewer's emphasis on our methodological innovation, - the decoupling of performance measures from underlying SemNet mechanisms -, as the

primary contribution. We have revised our Introduction to place greater emphasis on this methodological advance as the main motivation, while toning down language suggesting the topic is entirely unexplored:

> p.3: "Two experimental studies have provided indirect evidence in favor of the hypothesis that REM sleep is a hyperassociative state."

> p.3: "The relationship between sleep or sleep-related memory restructuring and creative problem-solving was investigated in several experimental studies, leading to disparate findings. Some studies used creativity tasks that were supposed to necessitate the formation of novel/distant semantic associations or a change in the representation of the problem (i.e., restructuring) (Bowden et al., 2005)."

> p.4: "Hence, the literature linking sleep, memory restructuring, and creative problem-solving is inconsistent."

R#2 Comment #4

"REM sleep supports neural plasticity and synapse reshaping" – this sentence and the whole paragraph surrounding it, need to be considered carefully. It is true that there are indications that REM sleep contributes to hyper-associativity, greater connectivity and bias towards excitation; however, there is also considerable evidence to the exact opposite, with REM sleep encouraging synaptic pruning and a bias towards inhibition. This known contradiction is, in fact, addressed and emphasized in the very first paragraph of one of the papers the authors cited (Aime et al., 2022). Please rewrite this paragraph to reflect the actual complexity regarding REM sleep as reflected in the literature.

We thank the Reviewer for this important clarification. We agree that REM sleep seems to involve complex and sometimes contradictory neuroplastic processes. Our use of 'synapse reshaping' was intended to capture this bidirectional nature of REM sleep plasticity - encompassing both synaptic strengthening and pruning processes as highlighted in Aime et al. (2022). The regional heterogeneity in REM sleep effects (with some areas showing enhanced plasticity while others undergo pruning) aligns with our hypothesis that REM sleep creates a neurochemically permissive environment for flexible memory reorganization, which may manifest differently across brain regions and circuit types. We have revised our wording to better reflect this complexity, as follows:

> p.2: "First, both the high level of acetylcholine (Hasselmo, 1999; Rasmusson, 2000) and GABA (Aime et al., 2022; Bridi et al., 2020; Niethard et al., 2016; Tamaki et al., 2020) during REM sleep supports neural plasticity and synapse reshaping. This neurochemical environment facilitates dynamic changes in synaptic connectivity, which may manifest as either synaptic strengthening or pruning depending on the brain regions and specific neural circuits involved (Aime et al., 2022; Poe, 2017; Ravassard et al., 2009)."

R#2 Comment #5

"higher level of EEG activation" (last sentence of the introduction): Please shortly clarify, already at this point, what does "high" mean in this context.

We clarified our EEG hypothesis, as follows:

p.5: "Finally, in an exploratory analysis, we examined whether REM sleep-related memory restructuring and/or creative benefit would be reflected by specific EEG markers, -such as faster cortical activity, higher complexity, or flatter spectral slope-, all thought to reflect a neurophysiological mode that is rich, with a wide repertoire of possible neural configurations, and allowing for complex cognitive processes to take place. We hypothesized that the cognitive complexity of memory restructuring should likewise be reflected in a richer cortical state."

R#2 Comment #6

Generally, the main weakness of the results is the lack of demonstrated association between performance on the riddle and either sleep or effects related to the SemNet metrics. The authors acknowledge it; however, they make little attempt to check these relations thoroughly on the pretense that too few REM subjects solved the riddle. But some things could still be examined across participant groups. For example, is there a relation between solvers (25 out of 111) on the first session and the baseline SemNet metrics? Is there a relation between solvers in the second session (10 out of 84) and differences in the SemNet metrics across sessions? Even in the absence of a sleep effect, it should still be examined whether the SemNet metrics have relevance to the ability to solve the riddle. I realize that this very question was the focus of the authors' 2024 paper – but as far as I could see in that earlier paper, the relation was only shown directly for the Weight metric, whereas many of the strongest results in the current work were for the EV metric. I would expect that the authors could at least try to replicate the 2024 result, and maybe even uncover additional relations between SemNet metrics and solving abilities. And if there are none, this should be clearly noted and addressed in Discussion (see later).

We thank the Reviewer for this relevant comment. In our previous study (Bieth et al 2024), we found that SemNet metric changes (and not $SemNet_{post}$ metric) were associated with successful problem-solving. Please note that in Bieth et al 2024, these results were already replicated in three independent datasets. While we agree that replicating those results again would be valuable, the current study's design differs fundamentally from our 2024 work, making direct replication challenging. Specifically: (1) the timing of SemNet computation differs between studies: in our 2024 paper, SemNets were computed before (pre-exposure) and after the solving phase, whereas in the current study, networks were computed immediately after the solving phase and again after the incubation period; (2) the current study lacks pre-problem SemNet assessment (baseline), preventing measurement of how semantic network change from baseline in response to problem exposure; and (3) an incubation period was introduced between SemNet assessments in the current design. The figure bellow illustrates these differences between the two studies.

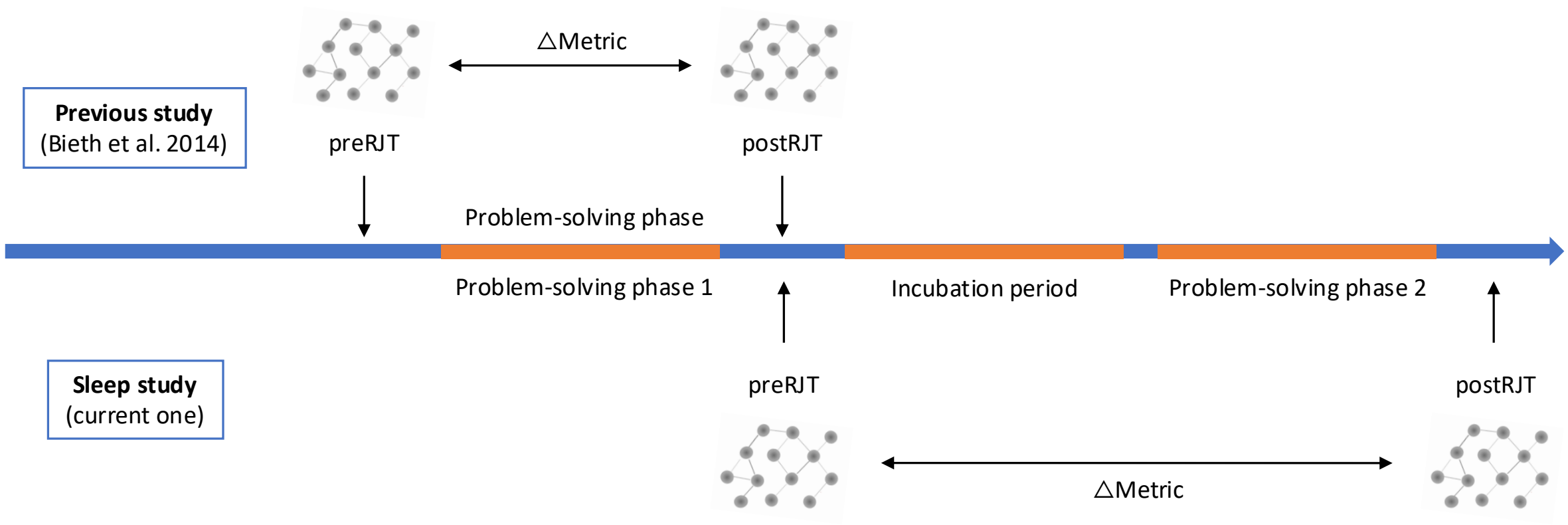

These methodological differences mean that the current study addresses complementary rather than replicable questions. Nevertheless, we investigated the Reviewer's suggestions by adapting statistical models used in our previous study in the current study.

First, we explored whether SemNet metric before the incubation period were associated with successful problem-solving during the first solving phase (i.e., before the incubation). If problem-solving is associated with remoteness-based SemNet changes, we could expect that the SemNet organizations after the solving phase differ between solvers and non-solvers. After the first problem-solving phase, 23 participants solved the riddle whereas 65 did not. We use a logistic regression with mixed-effect on the individual (Subject) to predict problem-solving before the incubation (isSolved1) with $SemNet_{pre}$ metric ($Metric_{Pre}$) and *semantic distance* variable (SD): $isSolved1 = \beta_0 + \beta_1 \times SD + \beta_2 \times Metric_{Pre} + \beta_3 \times Metric_{Pre} \times SD + (-1 + Metric_{Pre} \mid Subject)$. We found a statistically significant interaction effect of $SemNet_{pre}$ metric and *semantic distance* on problem-solving for *clustering coefficient* ($\beta_3 = .44$, $p = .001$). It suggests that semantically remote associations are more interconnected (higher *clustering coefficient*) in individuals who have solved the riddle compared to those who did not. We did not find significant association for *weight* ($\beta_3 = .04$, $p = .087$), *efficiency* ($\beta_3 = .04$, $p = .08$), and *eigenvector centrality* ($\beta_3 = .02$, $p = .71$). It is important to note though that, while we see a difference in SemNet organization between solvers and non-solvers pre-incubation, we cannot characterize this as 'restructuring' per se, as we lack a pre-problem baseline measurement and thus cannot quantify actual changes in network organization.

Second, we explored whether restructuring (measured with △Metric) was associated with successful problem-solving, regardless of the type of incubation (i.e., the group). After the second solving phase, six participants solved the riddle whereas 59 did not. We use a logistic regression with mixed-effect on the individual (Subject) to predict problem-solving after the incubation (isSolved2) with SemNet metric changes (△Metric) and *semantic distance* variable (SD): $isSolved2 = \beta_0 + \beta_1 \times SD + \beta_2 \times \triangle Metric + \beta_3 \times \triangle Metric \times SD + (-1 + \triangle Metric \mid Subject)$. We found statistically significant interaction effect of △Metric and *semantic distance* on problem-solving for *efficiency* ($\beta_3 = .10$, $p = .0046$), and *eigenvector centrality* ($\beta_3 = .21$, $p = .03$). These interaction effects indicate that larger positive changes in *efficiency* and *eigenvector centrality,* in semantically remote pairs/words, are associated with a higher solving success rate. We did not find such significant effect for *weight* ($\beta_3 = .006$, $p = .87$), and *clustering coefficient* ($\beta_3 = -.14$, $p = .53$).

In summary, despite factors limiting exact replication of our princeps results, we found several results that are nevertheless consistent with our primary findings. Interestingly, *weight*, *efficiency*, and *eigenvector centrality* were the metrics that captured significant REM sleep-related SemNet changes, as they did in our previous study (Bieth et al, 2024). It confirmed their potential in capturing variation in SemNet organization changes. Consequently, we rephrased the discussion to highlight the differences between the two protocols that makes replication hard to provide, as follows:

> p.17: "Interestingly, *weight*, *efficiency*, and *eigenvector centrality* were the metrics that captured statistically significant SemNet changes depending on the incubation period, as they did in our previous study (Bieth, Kenett, et al., 2024). However, direct replication of our previous findings is not possible here due to key differences in the experimental designs (**Figure S5**). Specifically, the current study lacks a pre-problem baseline assessment and includes an intervening incubation period with experimental manipulation between the two SemNet measurement time points, whereas our previous study computed networks before and after the solving phase without interruption. Despite these methodological differences that limit direct comparability, we nevertheless found several results that are consistent with our primary findings (see *Supplementary Note 6*)."

We proposed to include the additional analyses above in the *Supplementary Note 6* and **Figure S5**.

R#2 Comment #7

Many of the results seem to be related to effects of sleep as a whole rather than REM sleep in particular. More can be done to examine the specific contribution of REM sleep. For example, including the percentage of REM sleep as a covariate in the model (or run a follow-up analysis where the contribution of REM sleep percentage is examined). This can be carried out either over all sleep participants, or just those achieving REM sleep, or both.

We appreciate the Reviewer's suggestion. While we agree that including the percentage of REM sleep (pREM) could provide additional insights, we believe our current methodology already investigates the specific effect of REM sleep. Crucially, we analyze EEG markers specifically during REM sleep, which provides more precise and mechanistically relevant measures compared to simply quantifying time spent (or %) in REM sleep. Specifically, our EEG-derived metrics capture the actual neural activity patterns and physiological processes occurring during REM periods, rather than just their duration.

However, we acknowledge the value of examining REM sleep percentage as a complementary approach. We have therefore conducted additional analyses including REM sleep percentage as a covariate. We used Model 1, replacing the Group variable by pREM ($\triangle$Metric = β_1 x pREM + β_2 x SD + β_3 x pREM x SD + Error(Subject)). We did not find a statistically significant interaction effect for any of the four metrics (*weight*: $F(1,5415) = 1.26$, $p = 0.26$; *efficiency*: $F(1,5857) = .06$, $p = .81$; *eigenvector centrality*: $F(1,587) = 2.53$, $p = .11$; *clustering coefficient*: $F(1,587) = .10$, $p = .75$). These results suggest that the specific neural dynamics

during REM sleep, captured through our EEG marker, provide more sensitive and theoretically meaningful predictors of semantic network changes than REM sleep percentage.

We feel that the EEG analysis already provides evidence that brain activity during REM sleep is associated with memory restructuring. However, we will be happy to include the pREM analysis in Supplementary Information if the editor or reviewer feel strongly that it can be useful.

R#2 Comment #8

Please change the order of the results such that the analysis of performance on the riddle is presented first, and only then the SemNet results.

We followed the Reviewer's suggestion and now provide solving rates before the SemNet results.

R#2 Comment #9

"…and our results were consistent with our previous study (Bieth, Kenett, et al., 2024). The only deviating result concerns the lack of significant effect for global SemNet changes for efficiency" – again, that's not proven. The authors should check relations between solvers and SemNet metrics across participant groups to see if they replicate the relation to Weight found in the 2024 study. They then need to discuss it – especially if it's not replicated – in the context of their other findings.

Please see our response to the Reviewer #2 Comment #6 that addresses this point.

R#2 Comment #10

The authors mention N1 potential involvement in insight and cite Lacaux et al., 2021. Some results from my own lab using pink noise corroborated the potential importance of N1 in insight. Consider citing as well (Vickrey & Lerner, 2023). There might be more.

We thank the Reviewer for this relevant comment. We added this reference in the manuscript:

> p.18: "Promising studies also put forward the potential role of the sleep onset stage (N1) for creative thinking and insight problem-solving (Lacaux et al., 2021; Vickrey & Lerner, 2023)."

R#2 Comment #11

It is mentioned that additional tasks, unrelated to the ones presented in this paper, were carried out before and after. What is the risk that those other tasks affected the current one? Are these other tasks consistent across participants? More information needs to be supplied (perhaps in the Supplementary) to alleviate concerns.

As we mentioned, participants performed an additional task which aims at investigating analogical reasoning processes, and is out of the scope of the current study. We plan to analyze it in a further study. This task used figural material (i.e., numbers, letters, geometric shapes presented in different sizes, colors or positions). Briefly, in this task,

participants were asked to select a target (among two) that shared a relationship with a cue based on analogical reasoning. The task was composed of two parts: a training part, in which participants were trained on a given analogical rule, and a transfer part, in which they had to transfer this rule to new stimuli.

We believe that performing this task did not affect the riddle solving and RJT for several reasons. First, the analogy task involves a non-semantic material, which does not interfere with the semantic material used in the RJT. No semantic association can be made between the two sets of stimuli. Second, all participants, whatever their group (i.e., wake, NREM or REM group) did the same analogy task. Hence, it is unlikely that this additional task would affect the groups in different ways. Finally, the two parts of the task (i.e., training and transfer parts) were respectively done before starting the current study and at the very end of the experimental testing, minimizing its potential impact.

We now specify this point in the manuscript to avoid any confusion related to the reviewer's concerns, as follows:

> p.21: "Specifically, this task involves non-semantic and figural material that does not interact with the current experiment and was performed by all participants regardless of their group."

R#2 Comment #12

"Fischer test and corrected with Yates method" – I'm confused. Either you used a chi-square test - and then corrected with Yates if needed; or you used Fischer's exact test - and then no correction is needed at all. I'm not sure what "Fischer test" you refer to here.

We only used a Fischer's exact test to compare proportions. We apologize for this confusion concerning the methodology. We corrected our manuscript, as follows:

> p.25: We explored whether sleep improved creative problem-solving success by testing whether the solving rate was group-dependent using a Fischer's exact test.

Reviewer #3

R#3 Global comments

This paper investigates how different sleep stages contribute to creative problem-solving by examining changes in semantic memory networks before and after a 90-minute incubation period that included either NREM sleep, REM sleep, or wakefulness. Using fine-grained EEG analyses, the authors show that REM sleep promotes the reorganization of problem-related associations, consistent with its proposed role as a "hyperassociative" state. Interestingly, this REM-related restructuring was not sufficient on its own to enhance problem-solving success. Overall, this is a very strong and well-designed study, which provides valuable insights into the links between sleep and problem-solving, applying a highly innovative approach. Nevertheless, I have the impression that the manuscript focuses somewhat too heavily on REM sleep, despite also uncovering intriguing findings regarding NREM sleep that deserve more attention and discussion.

I suggest revising the manuscript, taking into consideration the comments below, before proceeding to the next step.

We thank the Reviewer for their overall positive assessment of our study, constructive feedback, and help improving our manuscript. We address all the comments in detail below.

Each of the Reviewer's comments are written below in italic, our response is in plain text, and the citations from the revised manuscript are highlighted in blue. We also made minor changes to further improve the manuscript, all highlighted in yellow.

R#3 Comment #1

"REM sleep dreams are typically longer (Antrobus, 1983; Casagrande et al., 1990; Foulkes, 1964), more vivid, hallucinatory (Hobson & Stickgold, 1994; Zadra et al., 1998), and metaphorical (Malinowski & Horton, 2021) than NREM sleep dreams." – Yes, this seems to be correct to some extent, nonetheless, some studies question this two-state model of dreaming and suggest that circadian factors might strongly influence the differences between NREM and REM dreams, indicating that nrem dreams become more similar to REM dreams in the morning hours. See for instance, Nielsen, T. A. (2004). Chronobiological features of dream production. Sleep Medicine Reviews, 8(5), 403-424.

We agree with the Reviewer's comment and now acknowledges the debate in the introduction. Changes can be found as follows in the manuscript:

> p.3: "Indeed, REM sleep dreams are typically longer (Antrobus, 1983; Casagrande et al., 1990; Foulkes, 1964), more vivid, hallucinatory (Hobson & Stickgold, 1994; Zadra et al., 1998), and metaphorical (Malinowski & Horton, 2021) than NREM sleep dreams (even if this is debated, see Nielsen, 2004)."

R#3 Comment #2

„For example, a study found that REM sleep facilitates solving of anagram word puzzles (Walker et al., 2002)." – Although the cited study aimed to highlight the role of REM sleep in creative problem solving, the authors should note that int hat specific study REM only showed an advantage versus NREM sleep, but the advantage was equal to that of waking.

The Reviewer is correct. We now specify this point as follows:

p.3: “For example, a study found that REM sleep facilitates solving of anagram word puzzles compared to NREM sleep (Walker et al., 2002).”

R#3 Comment #3

Were all the participants familiar with the hopscotch game? I believe this might largely influence the semantic networks activated by the riddle. How could the authors take this into consideration?

Thanks for the question. The hopscotch game is a very famous kid’s game in France where the starting square is named “earth” and the final one “sky”. While we did not directly assess participants' familiarity with this game through explicit questioning, all participants had a debriefing with the experimenter at the end of the experimental session. During this debriefing, we asked them, among other things, whether they understood the riddle after giving them the solution, and gave them the opportunity to freely comment. Four participants (3.6%) stated that they did not understand the riddle and its solution with one specifying that associating the verb “to land” and the word “sky” made no sense (thus reflecting a linguistic rather than game-familiarity issue). Among participants who reported understanding the riddle, three additional participants spontaneously mentioned that they did not know the game or that the last square was named “sky”.

Hence, although we did not explicitly ask participants whether they were familiar with the hopscotch game, most of them understood the riddle. However, we agree with the reviewer that the limited number of solvers could be partially attributed to game unfamiliarity. We added this point in the discussion, as follows:

p.17: “We cannot exclude that the limited number of solvers may be partially attributable to participants’ unfamiliarity with the hopscotch game, as we did not control for this factor.”

R#3 Comment #4

„First, participants who found the solution after the second RJT (n = 3) were excluded” – I do not understand this. In Fig1/A the RJT is after the 2nd attempt of riddle solving and not before.

As mentioned in the method section (see below), we asked the participants just after the second RJT whether they found a new solution to the riddle during the second RJT completion. This is not similar to a solving phase, but just a final question before continuing the experiment. The aim was to control whether the RJT may trigger the solution of the riddle.

We clarify this point as follows:

p. 22: “After they performed the RJT, we asked the participants whether they thought of a new response to the riddle during the RJT to ensure that participants did not solve the riddle during the RJT.”

R#3 Comment #5

The pre-established word pairs are supposed to reflect the semantic networks activated by the task; however, isn't it likely that different individuals follow different associative paths to solve the riddle, and may arrive to the same solution differently?

We thank the Reviewer for this interesting point. While the associative pathways to reach the solution may vary across individuals, we expect the terminal associations (sky-drawing) to be relatively consistent among successful solvers, as they must ultimately converge on the same conceptual endpoint. In addition, according to Schilling's (2005) theory, insight leads to the creation of new edges or 'shortcuts' in SemNets. Thus, while individuals vary in the associative paths they take when trying to solve the riddle, the end result should be similar in its effect on their SemNet, i.e., local memory restructuring as measured with our SemNet metrics. Nevertheless, we acknowledge that individual differences in associative routes represent a potential limitation of our approach that future studies could address through more flexible, personalized network approaches. We added this limitation in the Discussion as follows:

> p.17-18: "Second, due to practical constraints associated with building problem-related SemNets (including a long-time commitment and the need for riddle-specific semantic material), we chose to use only one riddle in this study (although our method was validated using four different riddles (Bieth, Kenett, et al., 2024)), with a limited number of words that may restrict the identification of less common associative pathways to the solution. In addition, according to Schilling's theory (2005), insight leads to the creation of new edges or 'shortcuts' in SemNets. Thus, while individuals vary in the associative paths they take when trying to solve the riddle, the end result should be similar in its effect on their SemNet, i.e., local memory restructuring as measured with our SemNet metrics. Our approach provides tools and methods to generalize the results using multiple riddles and larger SemNets across different sessions in future studies."

R#3 Comment #6

Excluding participants with many 0 ratings seems a bit arbitrary to me, as these responses were also allowed, and reflect valid responses (of a lack of perceived associations) for the word pairs.

This comment echoes Reviewer #2 Comment #1. Please, refer to the reply to this comment.

Briefly, we justified participant exclusions for three key reasons: (1) the distribution of responses indicates that most excluded participants exhibited response patterns that substantially exceeded reasonable variability (see *Supplementary Note 7* and **Figure S6**). Among the 14 excluded participants, three had a high proportion of non-responses (22%, 36%, and 16%), which represents data loss that affects network computation. The other participants (n = 10) had a high proportion of zeros (mean = 34%, SD = 16%, minimum 17%, maximum 62%, > 20% for 9 of them). We observed that the threshold choice did not drastically change the count of excluded participants; (2) a high proportion of zeros and non-responses considerably affect the SemNet metric computation and would introduce a bias that is

particularly problematic for longitudinal analyses; and (3) zero responses are inherently ambiguous and may reflect disengagement rather than true absence of associations.

To reassure the Reviewer on the robustness of our findings, we ran our main behavioral analyses with all participants who did not solve the riddle pre-incubation, including the 14 participants we excluded in our original manuscript. In sum (but please, see Reviewer #2 Comment #1 for details in our analyses), our results remained very similar when including participants who have been excluded based on the proportion of zeros/non-responses during the RJTs. In particular, our main results showing that REM sleep was associated with remoteness-based SemNet changes still hold in the full sample. The only deviating results were: (i) the loss of some results concerning the sleep-related global restructuring, and (ii) the loss of significant differences between REM vs NREM, and NREM vs Wake, in the post-hoc tests for *eigenvector centrality* (however, our key result, -the difference when comparing REM and Wake groups- remains significant).

R#3 Comment #7

Although the authors controlled for confounds such as TST to exclude the possibility that sleep duration and not specifically REM sleep was associated with the change in Semantic relatedness metric, based on the first table, the REM and NREM groups also differed in N2, SWS and WASO, however, these confounds were not taken into consideration.

We thank the reviewer for this important observation. We acknowledge that the REM and NREM groups differed in multiple sleep parameters beyond TST, including N2, SWS, and WASO. Specifically, the NREM group exhibited more fragmented sleep (higher WASO) and lighter sleep architecture (reduced N3) compared to the REM group, potentially reflecting lower sleep pressure in the NREM group.

While we controlled for TST as our primary measure of sleep duration, we recognize that other sleep parameters may contribute to semantic restructuring. However, we note that WASO is highly correlated with TST (rho = -.60, $p = 7.45 \times 10^{-6}$), suggesting that controlling for both variables simultaneously would introduce multicollinearity concerns and substantially increase the number of statistical tests, thereby elevating the risk of Type I errors without necessarily improving model interpretation.

Importantly, our findings provide strong evidence for REM sleep-specific effects, as evidenced by distinct EEG markers associated with semantic restructuring that were present during REM sleep. Additionally, our exploratory analyses revealed preliminary EEG indicators suggesting potential NREM contributions to restructuring processes, though these findings are less interpretable.

As we already acknowledge in our manuscript, we cannot isolate the effect of 'pure' REM sleep independently from NREM sleep, and the neural mechanisms underlying semantic restructuring, and when exactly such reorganization occurs during naps, remain unclear. Our study design precludes definitive conclusions about whether NREM sleep initiates restructuring processes that subsequently unfold during REM sleep, or whether both stages contribute independently to SemNet changes. We have added clarification regarding these limitations to our revised manuscript, as follows:

> p.18-19: "Finally, because REM sleep occurs after other sleep stages, we were unable to isolate the effect of 'pure' REM sleep independently from NREM sleep. A few studies have highlighted NREM sleep as a state favorable to problem-solving (Beijamini et al.,

2014, 2021; Sanders et al., 2019). Promising studies also put forward the potential role of the sleep onset stage (N1) for creative thinking and insight problem-solving (Lacaux et al., 2021; Vickrey & Lerner, 2023). In our study, we found a higher remoteness-based SemNet changes in the NREM group than in the Wake group for one of our local metrics (eigenvector centrality). The neural mechanisms underlying the restructuring of semantic associations and when exactly such a reorganization occurs during the nap remain unknown. In an exploratory analysis, we found several EEG features associated with restructuring processes that differed according to the sleep stage. Even though we applied a rigorous correction for multiple comparisons, the results must be interpreted with caution and need to be replicated in further studies. In particular, further researchs are needed to clarify the role of each NREM sleep stage and whether NREM sleep is necessary to initiate the restructuring processes that would unfold during subsequent REM sleep. Moreover, our groups also differed in sleep fragmentation and time spent in different NREM stages, which may represent additional factors that could contribute to semantic restructuring either independently or in interaction with REM sleep processes."

R#3 Comment #8

The authors accentuate the specific effect of REM on semantic networks; however, in many outcome measures, REM and NREM sleep do not show significant differences. Do the authors consider that this is due to a lack of power? It is also interesting that the variability in the REM group seems to be much larger, suggesting that participants reaching REM considerably differ. Could the authors further explore why the variability in the REM group is much higher compared to the NREM and wake groups?

While we observed differences between the Wake and REM groups on most SemNet metrics for global restructuring (main effect of Group), it is true that we did not find significant differences between the NREM and REM group for this analysis (or the dominant representation analysis). To be fair, there were no differences between the Wake group and the NREM group either, making it hard to interpret what happens during NREM sleep compared to the other groups. In all analyses, the data looked like a staircase, with the lowest ΔMetric in the Wake group, the highest ΔMetric in the REM group, and the NREM group falling in the middle (Figure 2A). It is therefore likely that, depending on the variability and magnitude of the effect size, statistical significance may or may not be reached when comparing the NREM group to the Wake or REM groups, or when analyzing specific metrics. Another possibility is that memory restructuring already starts during NREM sleep. This idea fits with our EEG results showing that some EEG markers during NREM sleep are also associated with SemNet changes.

Importantly, our general conclusion that REM sleep is a hyperassociative state does not rely solely on this analysis. Crucially, we did find significant differences both between the Wake and REM groups and between the REM and NREM groups for local, remoteness-based restructuring (interaction Group x *semantic distance*) for two of our metrics (*efficiency* and *eigenvector centrality*), which directly supports our primary hypothesis. In addition, we found specific EEG markers during REM sleep associated with remoteness-based SemNet changes.

We agree with the reviewer that the variability of $\triangle$Metric in the REM group seems to be larger than in the Wake or NREM groups. Please note that this variability is taken into account by our statistical mixed-effect model. The reasons for this higher SemNet metric variability after REM remain to be explored. One possibility may be that REM sleep also fostered SemNets changes that are individual-specific but unrelated to the riddle.

R#3 Comment #9

In Fig4/E - I recommend to highlight with different colours the participants from the REM and the NonREM only groups, to explore whether the NREMalpha-metric correlations are present in both groups or only in the REM group.

Thanks for the insightful suggestion. We changed **Figure 4** by adding a color code indicating the participants' group. The distribution suggests that an association between remoteness-based SemNet changes and alpha EEG power in NREM is present in both group.

We added the new figure in the manuscript and modified the legend consequently, as follows:

> p.14: "Letters inside the red-framed squares correspond to the figure panels that provide additional graphical representations of these results (from **B** to **G**), showing the regression coefficient between $\triangle$Metric and SD as a function of an EEG marker with each dot representing an individual participant using a color code indicating their group (REM group in blue and NREM group in red)."

R#3 Comment #10

"Of note, the absence of difference between the NREM and REM groups in some of our behavioural analyses and the NREM-related EEG results also suggests that the contribution of sleep in restructuring processes may be more complex, possibly involving the different NREM sleep stages that precede REM sleep." – This hypothesis could be tested by combining REM and NREM EEG measures as predictors of semantic network change.

As suggested by the Reviewer, we ran additional analyses aiming to explore this hypothesis. We compared different statistical models that include EEG markers computed during both NREM and REM sleep. We progressively complexified the models to explore whether adding information about NREM sleep would better explain the variability explained by REM sleep alone (and conversely). We used SemNet metrics that showed significant results in our EEG analyses in both NREM and REM sleep (i.e., *weight* with theta power in REM sleep and alpha power in NREM sleep; and *efficiency* with $Slope_{1/f}$ in REM sleep and alpha power in NREM sleep). We compared the simplest model with successively more complex ones by computing analysis of variance tables using the *Anova* function in r.

The models with increasing levels of complexity for the *efficiency* can be formalized as follows:

(A) : $\triangle$Efficiency = SD x $Slope_{1/f}$[in REM]

(B) : $\triangle$Efficiency = SD x $Slope_{1/f}$[in REM] + SD x Alpha[in NREM]

(C) : △Efficiency = SD x $Slope_{1/f}$[in REM] x Alpha[in NREM]

(D) : △Efficiency = SD x Alpha[in NREM]
(E) : △Efficiency = SD x Alpha[in NREM] + SD x $Slope_{1/f}$[in REM]
(F) : △Efficiency = SD x Alpha[in NREM] x $Slope_{1/f}$[in REM]

Results are summarized in the Table below. Briefly, we found that Model B and Model C were not significantly better than Model A (p = .91 and p = .44, respectively) to explain △Efficiency. In contrast, we found that Model E and F were significantly better than Model D (p = .004 and p = .006, respectively) to explain △Efficiency. Of note, Model F was not significantly better than Model E (p = .17). These results suggest that the most parsimonious model that explains our data is the one with REM sleep alone and that adding NREM sleep does not increase our explanatory power.

	npar	AIC	BIC	logLik	deviance	Comparison	Chisq	Df	*p*
**A**	6	16535	16576	-8261.7	16523	-	-	-	-
**B**	8	16539	16593	-8261.6	16523	B > A?	.18	2	.91
**C**	10	16540	16606	-8259.8	16520	C > A?	3.77	4	.44
**D**	6	16546	16586	-8267.2	16534	-	-	-	-
**E**	8	16539	16593	-8261.6	16523	E > D?	11.05	2	.004
**F**	10	16540	16606	-8259.8	16520	F > D?	14.63	4	.006
						F > E?	3.58	2	.17

We applied the same approach for the *weight*. The models with increasing levels of complexity can be formalized as follows:

(A) : △Weight = SD x Theta[in REM]
(B) : △Weight = SD x Theta[in REM] + SD x Alpha[in NREM]
(C) : △Weight = SD x Theta[in REM] x Alpha[in NREM]

(D) : △Weight = SD x Alpha[in NREM]
(E) : △Weight = SD x Alpha[in NREM] + SD x Theta[in REM]
(F) : △Weight = SD x Alpha[in NREM] x Theta[in REM]

Results are summarized in the Table below. We found that Model B was not significantly better than Model A (p = .11) to explain △Weight. However, Model C was significantly better in explaining △Weight than Model A (p = .001). In addition, we found that Model E and F were significantly better than Model D ($p = 1.60 \times 10^{-4}$ and $p = 2.86 \times 10^{-6}$, respectively) to explain △Weight. Finally, Model F was significantly better than Model E (p = .001). Contrary to the result obtained with *efficiency*, these results suggest that the most parsimonious model that explains our data is the one combining REM and NREM sleep including an interaction factor between them.

	npar	AIC	BIC	logLik	deviance	Comparison	Chisq	Df	*p*
**A**	6	15447	15487	-7717.7	15435	-	-	-	-
**B**	8	15447	15500	-7715.4	15431	B > A?	4.49	2	.11
**C**	10	15427	15503	-7708.6	15417	C > A?	18.15	4	.001
**D**	6	15460	15500	-7724.2	15448	-	-	-	-
**E**	8	15447	15500	-7715.4	15431	E > D?	17.49	2	$1.60\ 10^{-4}$
**F**	10	15437	15503	-7708.6	15417	F > D?	31.14	4	$2.86\ 10^{-6}$
						F > E?	13.66	2	.001

We chose not to include these exploratory analyses in the main manuscript as our experimental design and main objective focused on REM sleep. In addition, these new analyses were not all consistent, were driven by our first results, and do not take into account the whole data.

We believe our findings provide a solid framework for further targeted investigation to validate this emergent hypothesis, using pre-planned experimental designs and statistical analyses, as indicated in the discussion. This additional data is now available on the public persistent repository.

<u>Reviewer #1</u>

R#1 Global comments

I thank the reviewers for their quick and thorough responses to my comments. Nearly all my previous concerns have been sufficiently addressed, with the exception of the analysis of whether the REM group was "closer" to the correct solution than other participants. I agree that the analysis can still be reported, I just feel that there should be a brief explanation of how this idea of incremental steps leading one closer to the solution should be reconciled with the "eureka" feeling reported by solvers. It is unclear why participants were asked to report if they had a eureka feeling or not and what this tells us then. Is it that the subjective eureka experience comes suddenly, but only after a sufficient amount of incremental restructuring has taken place?

We appreciate the Reviewer's positive feedback. We address all the comments in detail below. Each of the Reviewer's comments is written below in italic, our response is in plain text, and the citations from the revised manuscript are highlighted in yellow.

We thank the reviewer for this helpful comment. We agree that the relation between gradual progress toward a solution and the subjective experience of Eureka should be clarified. In our view, these two aspects are not contradictory.

The Eureka report captures the subjective phenomenology of the solution moment, that is, whether the answer entered awareness suddenly and with an insight-like feeling. We collected Eureka reports as our previous work demonstrated that restructuring is associated with insight solving. Unfortunately, the insight solving rate was insufficient to allow statistical analyses in the current study.

While our collection of insight solving via the Eureka report is classical in creativity research involving problem-solving (Laukkonen & Tangen, 2018), our approach to collecting all candidate solutions that came to mind is original, and it provides a window into the dynamics of the search space and its closeness to the solution space, i.e., the dynamics of problem-solving.

Our "closeness to the solution" analysis was intended to capture whether participants' responses reflected partial progress toward the correct solution, regardless of whether the final solution was reached. A participant may therefore move incrementally closer to the solution through gradual restructuring (as shown in Durso et al., 1994, experiment 2; see also Bilalic et al., 2019; Cushen & Wiley, 2012; Danek et al., 2020), while still experiencing the final emergence of the correct answer as sudden. In this sense, the subjective Eureka experience may reflect the moment at which a sufficient amount of prior restructuring has accumulated to make the solution consciously accessible.

So, to clarify, the purpose of collecting Eureka reports was to distinguish subjective insight solving from non-insight solving, but we did not necessarily assume that restructuring must itself occur in an all-or-none fashion.

In the method section of the manuscript, we explicitly stated that insight and non-insight solving methods were not mutually exclusive; they could occur successively during the same trial when considering different ideas, and we instructed participants to consider only

the few seconds before the idea came to mind when answering the Eureka question. We also clarified that getting closer to the solution is not synonymous with analytical solving.

Accordingly, we revised the method section describing the problem-solving phase and added the following information:

> p.19: "It was opposed to analytic solving where individuals can explain the internal steps and mental operations leading to the solution.".

Also, as a side note, I agree with the author's response to Reviewer 2, where they note that research concerning the role of REM sleep in semantic restructuring is relatively sparse compared with the broader literature. However, the authors may be interested in the following study that was not cited but also addresses this issue: Westerberg CE, Fickle SE, Troupe CE, Madden-Rusnak A, & Deason RG (2025). An afternoon nap facilitates analogical transfer in creative problem solving. Journal of Sleep Research, 34(4), e14419. https://doi.org/10.1111/jsr.14419

We indeed missed this relevant reference, -thanks for sharing it. We now cite this interesting study in the introduction:

> p.3: "For example, a study found that REM sleep facilitates solving of anagram word puzzles compared to NREM sleep (Walker et al., 2002) and a recent study showed that greater REM sleep proportion during an afternoon nap was linked to successful analogical transfer in creative problem-solving, suggesting that REM sleep supports the formation of novel connections between source and target problems (Westerberg et al., 2025)."

We also added a recent study that showed an association between EEG markers of cognitive richness with creativity task:

> p.3: "Such EEG measures have been linked to enhanced cognitive processing, allowing to discriminate between different states of consciousness (e.g., wake vs. anesthesia) (Sitt et al., 2014), but also between varying levels of engagement in a cognitive task during wakefulness (Höhn et al., 2024; Zhang et al., 2026) and responsiveness to auditory stimuli during sleep (Türker et al., 2023)."

> p.14: "This is in line with a few recent studies which showed variations in the values of these EEG markers during simple cognitive tasks performed during wakefulness (Höhn et al., 2024; Zhang et al., 2026), the sleep onset period (Strauss et al., 2022) and bona-fide sleep (Türker et al., 2023)."

Reviewer #2

R#2 Global comments

I appreciate the authors' detailed answers to my comments. Their responses to most of them are satisfactory. However, regarding my two core concerns, I felt that their reply was mostly aimed at explaining away my worries rather than making significant changes to the manuscript, which I find insufficient. Before I go into the specifics, let me explain my general approach from a bird's eye-view: There are many ways to analyze data, which often yield many results – some stronger, some weaker. The way weaker results are handled depends on how meticulous the researchers want to be. Should everything be reported, even if the evidence is weak and the statistical control is not as rigorous as it can be? or should only the stronger results be reported, knowing that you are walking on firmer ground? Usually it's something in between, but my view and experience is that there is a lot of noise that often gets mixed with true signal and doesn't reflect true effects, and the better the journal (and I consider Communications Biology a respectable platform) – the higher the emphasis should be on the stronger results, without attempting to milk findings to the last drop. The current study yielded many results, not always consistent with each other; but some results were clearly stronger than others, passing more rigorous statistical control – and those are the ones the manuscript should be focused on if accepted for publication.

We thank the Reviewer for their overall positive assessment of our revisited manuscript. We address all the comments in detail below. Each of the Reviewer's comments is written below in italic, our response is in plain text, and the citations from the revised manuscript are highlighted in yellow.

We understand the global comment of the Reviewer and propose a restructuring of our manuscript. The revisited manuscript now emphasizes our main robust results (i.e., the REM sleep-related remoteness-based SemNet changes at the behavioral and EEG levels, problem-solving rates regarding the group). We moved to Supplementary Information, additional or less robust results (i.e., REM sleep-related global SemNet changes, REM sleep-related effect on the dominant representation of the problem, and the analysis of getting closer to the solution).

R#2 Comment #1

Regarding removal of participants: I still find the 15% threshold to be quite arbitrary, and I do not agree with some of the authors' arguments. Let's treat things on a case-by-case basis. Regarding participants with high levels of non-responses, I am more inclined to accept the authors' justification that their data skews the network computation – missing data can do that – even though I have to admit that looking at the distribution, only the one with 36% seems like a true outlier. Regarding the participants with high proportions of zero rankings – while a value of 0 can in principle reflect all of the possibilities the researchers state rather than participants' true evaluation of the semantic distance, these are merely speculations. From my own personal experience with obtaining semantic relatedness ratings from subjects, if you ask them about it later – they would often stick by their extreme (e.g., zero) ratings. People's opinions vary widely on those things, often much more than what researchers seem to think are the "right" values. Either way, removing a big number of participants because the

computational model suffers from their extreme value seems to be bad practice unless done very sparingly. The authors should apply an acceptable and strict threshold for removal of participants (maybe something like answers with >5 MAD from the median? I can't really tell without seeing the specific numbers, but it should be a something that can be supported by standard outlier removal methodology) that doesn't end up removing a high proportion of their participants). Looking at the histograms, the eye test suggests, as mentioned above, that only one participant is an outlier for the non-response participants, and maybe 2-4 for the zero proportion participants; but obviously the determination needs to be by the values rather than the eye. The authors should then report the results running on that exclusive number of participants in the main text; and only in the supplementary, if they insist, present results after applying a higher removal of participants.

Additional sidenote: The authors mention (and I see in the histograms) 10 people removed due to zero proportions and 3 removed due to missing value. That sums up to 13. Where is the 14th removed participant coming from?

We thank the reviewer for this thoughtful comment. We agree that participant exclusion should be applied sparingly and that an arbitrary threshold is not ideal unless it can be clearly justified. We also appreciate the reviewer's point that extreme zero ratings may in some cases reflect genuine subjective judgments rather than invalid responding. At the same time, our concern is slightly different: in the present study, high proportions of zero ratings are problematic not only because they may reflect extreme responding, but because they can substantially distort the estimation of SemNet structure itself. This issue is particularly important in our longitudinal design, where our key outcome depends on change in network structure from pre- to post-test. Thus, an inflated proportion of zero ratings at either time point can have a disproportionate effect not only on the network estimated at that session, but also on the derived measure of restructuring (Post – Pre).

To make this clearer, we have now added a new visualization showing the proportion of zero ratings at pre-test against post-test for each participant, with the 15% threshold indicated on both axes and excluded participants highlighted in red. We believe this makes the longitudinal issue more transparent than separate histograms (as displayed in our previous rebuttal), because it shows that problematic cases are not confined to the pre-test distribution: several participants show elevated proportions of zero ratings at both tests, and some exceed the threshold at only one of the two sessions. In a longitudinal network analysis, such cases are especially problematic because they can spuriously inflate or attenuate estimates of change.

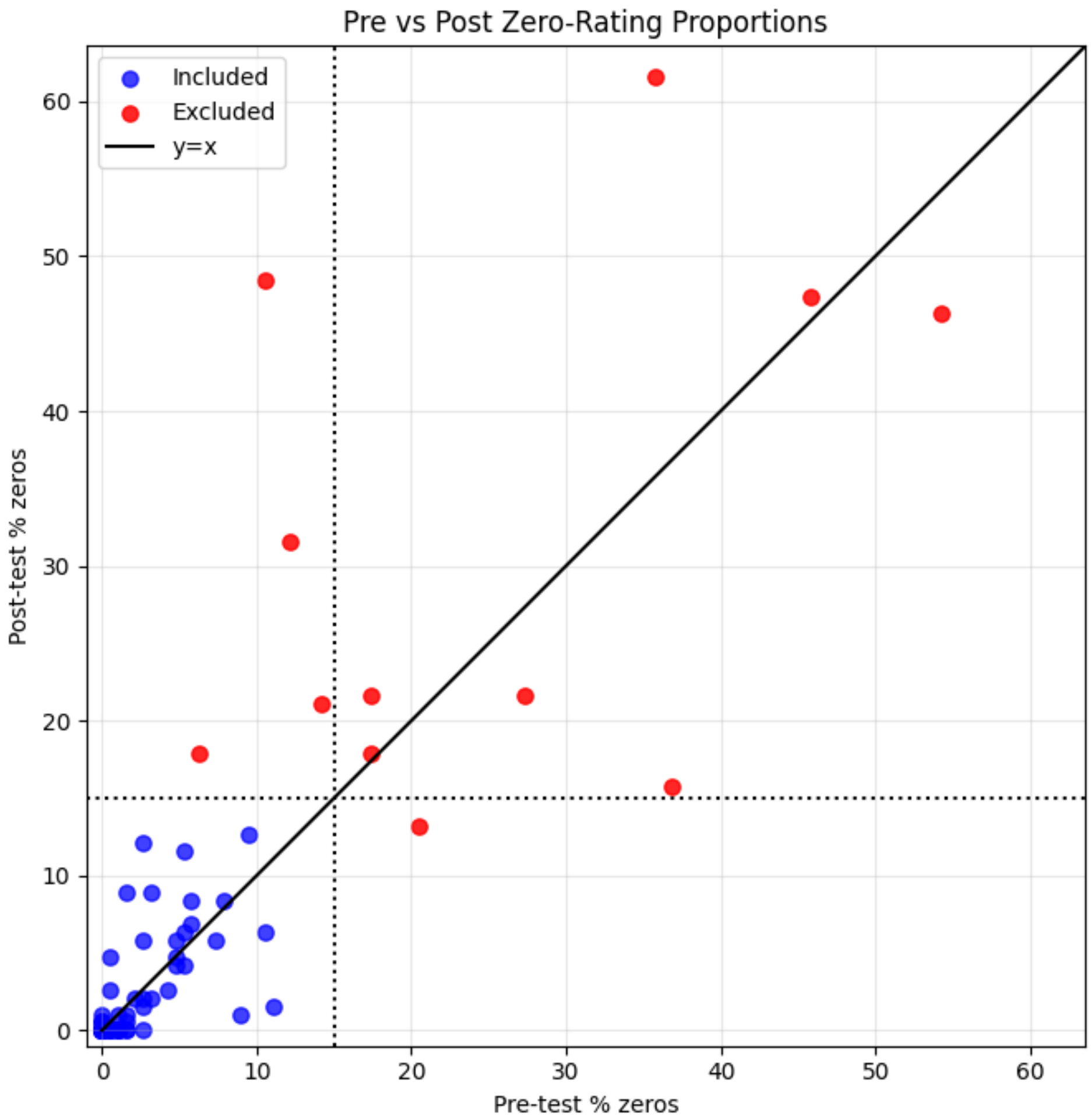

Following the reviewer's suggestion, we also explored a more formal robust outlier criterion based on the median absolute deviation (MAD). In our data, the median proportion of zero ratings was 1.32% at pre-test and 0.79% at post-test, with corresponding adjusted MADs of 1.95 and 1.17, respectively. A threshold of median + 3 × adjusted MAD would therefore correspond to 7.17% at pre-test and 4.30% at post-test, and even a threshold of median + 5 × adjusted MAD would correspond to 11.07% at pre-test and 6.64% at post-test. Likewise, using the conventional modified z-score cutoff of 3.5 would imply thresholds of approximately 11.57% at pre-test and 6.94% at post-test. These values are substantially lower than our 15% criterion because the distribution of zero-rating proportions is bounded at 0 and highly concentrated near zero.

This analysis confirmed two points. First, the participants excluded under our original criterion are indeed located at the extreme end of the distribution. Second, a formal MAD-based rule becomes very stringent in this dataset and would exclude many additional participants (n=14 additional participants) relative to our 15% criterion. In our view, this makes the MAD approach useful as a sensitivity analysis, but too restrictive as the primary exclusion rule for these bounded and zero-inflated data.

We therefore retained the 15% threshold in the main analysis, while clarifying its rationale more explicitly. We do not present it as a universal or purely statistical cutoff, but rather as a pragmatic criterion chosen to balance two considerations: (a) limiting the influence

of participants whose high proportion of zero ratings would disproportionately distort SemNet estimation, especially in a longitudinal pre–post comparison, and (b) avoiding the exclusion of a large proportion of participants under a stricter distribution-based rule. In line with the reviewer's suggestion, we now report the MAD-based sensitivity analysis and clarify that the main conclusions are not driven by the broader exclusion rule.

We modified the methods section as followed:

p.20-21: "Finally, we excluded participants based on their RJT completion quality. Individuals with more than 15% of non-responses (i.e., no response within the allotted time) and/or ratings of zero in the first and/or second RJT were excluded (n = 14, 11 for inflated proportion of zeros, two for inflated proportion of non-responses, and one for both). This threshold was adopted as a pragmatic quality-control criterion rather than as a formal statistical outlier cutoff. This exclusion was motivated by three considerations: (1) zero ratings and non-responses are inherently ambiguous and may reflect disengagement rather than true absence of associations, (2) in a longitudinal design, inflated number of zero ratings or non-responses at either time point can systematically distort the detection of true within-subject changes in semantic structure, and (3) zero ratings have a disproportionate impact on SemNet metrics relative to any other response values (see *Supplementary Note 8*). As a sensitivity analysis, we additionally examined unusually high proportions of zero ratings using a robust outlier approach based on the median absolute deviation (MAD), computed separately for pre-test and post-test. Specifically, modified z-scores were calculated as $M_i=0.6745(x_i-\text{median})/\text{MAD}$, with the conventional threshold of 3.5 used to flag extreme cases. The constant 0.6745 scales the MAD-based score to be comparable to a standard z-score under normality. Zero-rating proportions were strongly right-skewed and concentrated near zero; the median was 1.32% at pre-test and 0.79% at post-test, with corresponding adjusted MAD values of 1.95 and 1.17. These robust dispersion estimates implied substantially lower data-driven thresholds than the prespecified 15% criterion, indicating that a MAD-based rule would be overly stringent for these bounded and zero-inflated data. We therefore retained the 15% rule as the primary exclusion criterion. Although arbitrary, this threshold effectively identifies participants whose response patterns deviate substantially from the sample distribution (see data distribution on **Figure S8**) and whose inclusion would risk compromising network stability and interpretability while avoiding overly aggressive participant removal."

Side note: In total, 19 participants were excluded for reasons related to the RJT. Specifically, 3 participants solved the riddle during the RJT, 2 were excluded because of technical issues during the task, 12 were excluded because they showed an inflated proportion of zero ratings, and 3 were excluded because they showed an inflated proportion of missing responses (NaNs). One of these latter participants had already been excluded for an inflated proportion of zero ratings.

R#2 Comment #2

Regarding correction of multiple comparisons: Again, while I appreciate the reviewer's argument that their behavioral hypotheses are a-priori and therefore should not be corrected for multiple comparisons, this is, at the bottom line, a decision of how statistically strict they want to be. It is not obvious to me that the 4 parameters mentioned are a subset of a bigger set of parameters, some of which are expected to yield effects, and some aren't (thus justifying concentrating on them without correction). In the Bieth et al (2024) paper, these are in fact the exact 4 parameters reported. The fact is that if the authors choose to be strict, their results would be more compatible with the alternative bootstrapping analysis in the supplementary. This, to me, is a good reason to focus on the strong result rather than report the weaker ones. The authors should definitely begin by reporting that strong result as their main finding (correcting for multiple comparisons). Only then, either in the supplementary or in a subsection of the main results, indicate that if they loosen their criterion and not correct for multiple comparisons, they also get these and these additional effects. They can then discuss the strength of the different results and their implications in the discussion (which they seem to have already began to). I would especially appreciate an interpretation of how they see their results in case only the strong finding is true and the rest is noise. Importantly, note that the final findings in this analysis will also probably be influenced to a degree by the inclusion of the omitted participants (my previous bullet point).

As mentioned before, we revisited our manuscript to report only the strongest results and move to *Supplementary Note* the other ones. It allows us to emphasize our main result and make our manuscript easier to read. We thank the Reviewer for that.

In addition, as the Reviewer asked, we corrected our results for multiple comparisons to increase their statistical reliability. Results that did not survive the correction were mentioned in the Supplementary Note.

Changes can be tracked as follows:

> p.9: "In addition, the main effects of Group on ∆Metric did not survive the correction for multiple comparisons (see *Supplementary Note 3*, **Figure S3**), suggesting that the impact of sleep on SemNet restructuring is mainly local rather than global.
>
> p.10: "To further interpret these results, we ran additional exploratory analyses detailed in *Supplementary Note 2*."
>
> p.24: "Results were corrected for multiple comparisons using the Bonferroni method (significance threshold for *p*-values fixed at 0.05/4, i.e., 0.0125)."
>
> p.25: "We ran the same model independently for each network metric that we computed (*weight*, *efficiency*, *clustering coefficient,* and *eigenvector centrality*). Results were corrected for multiple comparisons using the Bonferroni method (significance threshold for *p*-values fixed at 0.05/4, i.e., 0.0125)."

R#2 Comment #3

Regarding previous literature: I appreciate the authors toning down their statements; still, they should be aware that there are more works showing links between REM sleep and creativity in the broader sense than they cite in the manuscript or mention in their response (e.g., Alger & Payne, 2016; Batterlink et al, 2014; the latter relates to semantic memory). If we are talking about links to sleep in general, or SWS, there are significantly more, including related to semantics (Tamminen et al, 2010 jumps to my mind; see my review, Lerner & Gluck, 2019 which the authors cite, for some more).

Our introduction focused on creative problem-solving. The references the Reviewer proposed concern the effect of sleep on memory consolidation or integration as measured using memory tasks. They did not refer to creative problem-solving . Although scientifically interesting, we believe that they do not fit within the scope of our study. Considering that our manuscript is already long (119 references), and that we cited the most relevant and influential articles in the domain, we decided not to include them.

However, while reviewing the literature again, we came across a relevant article linking a creativity task with EEG markers of cognitive richness. We included it to emphasize the rationale we provided regarding the use of EEG markers in our study:

> p.3: "Such EEG measures have been linked to enhanced cognitive processing, allowing to discriminate between different states of consciousness (e.g., wake vs. anesthesia) (Sitt et al., 2014), but also between varying levels of engagement in a cognitive task during wakefulness (Höhn et al., 2024; Zhang et al., 2026) and responsiveness to auditory stimuli during sleep (Türker et al., 2023)."

> p.14: "This is in line with a few recent studies which showed variations in the values of these EEG markers during simple cognitive tasks performed during wakefulness (Höhn et al., 2024; Zhang et al., 2026), the sleep onset period (Strauss et al., 2022) and bona-fide sleep (Türker et al., 2023)."

Reviewer #3

R#3 Global comments

The authors have adequately addressed my questions. I appreciate the additional analyses performed to study the combined role of NREM and REM sleep on performance.

We appreciate the Reviewer's positive feedback.

Reviewer #1

R#1 Global comments

The authors have adequately assuaged my concern regarding how the incremental steps that lead to a problem solution are reconciled with the "eureka" feeling that occurs when the solution is realized and have incorporated my suggested citation about the role of REM in semantic restructuring and I have no further suggestions.

Regarding Reviewer 2's comments 2 and 3, I think the authors have sufficiently addressed the concerns. They have controlled for multiple comparisons and moved any analyses that didn't survive the correction to the supplementary information. I also agree with the authors that they do not need to include further citations about REM sleep and creativity "in the broader sense". The articles suggested by the reviewer were focused on memory, not creative problem solving, and the manuscript already includes the citation for a review article that summarizes many of those studies.

For comment 1 from Reviewer 2, I commend the authors for their extensive consideration of how best to exclude outliers. They completed an analysis using MAD to identify outliers as suggested, which ended up excluding even more participants than their original method. They have provided an extensive explanation for the decision to stick with the 15% cut-off and are transparent by providing the data in supplementary figure 8. I think that is the best that can be done here. However, there is one part that is still confusing. In the "side note" the authors included in their response to this comment, it says that 19 participants were excluded for reasons related to the RJT. In the manuscript on p. 8, it says 21 were excluded for "various task-specific reasons". However, in the method section on p. 20, it says 19 were excluded. Is the 21 on p. 8 a typo? If not, why were the other 2 participants excluded?

We appreciate the Reviewer's positive feedback. We apologize for the lack of clarity the Reviewer highlighted. As indicated in the Supplementary Figure 1, the exclusion of participants involves the following sequential steps:

- First, participants in the Wake group who slept during the incubation period (n = 2).
- Then, participants who solved the riddle before the incubation period (n = 25).
- Finally, for various task-specific reasons (n = 19) including participants who solved the riddle during the second RJT (n = 3), for technical issues during the tasks (n = 2), and based on RJT completion criteria (n = 14).

We clarify this part as follows:

p.5: "Several participants (n = 46) were excluded, 25 because they solved the riddle in the first solving phase, two because they fall asleep during the resting period while in the wake group, and 19 for various task-specific reasons (see Methods)."

p.15: "In addition to removing participants who solved the riddle in the first solving phase (n = 25) and those who fall asleep in the Wake group (n = 2), we excluded 19 additional participants for several reasons related to the RJT (Supplementary Figure 1)."
